# An aniline dication-like transition state in the Bamberger rearrangement

**DOI:** 10.3762/bjoc.9.119

**Published:** 2013-06-03

**Authors:** Shinichi Yamabe, Guixiang Zeng, Wei Guan, Shigeyoshi Sakaki

**Affiliations:** 1 Fukui Institute for Fundamental Chemistry, Kyoto University, Takano-Nishihiraki-cho 34-4, Sakyo-ku, Kyoto 606-8103, JAPAN. Phone: +81-075-711-7907

**Keywords:** Bamberger rearrangement, DFT calculations, *N*-phenylhydroxylamine, proton transfer, reactive intermediates, transition states

## Abstract

A Bamberger rearrangement of *N*-phenylhydroxylamine, Ph–N(OH)H, to *p*-aminophenol was investigated by DFT calculations for the first time. The nitrenium ion, C_6_H_5_–NH^+^, suggested and seemingly established as an intermediate was calculated to be absent owing to the high nucleophilicity of the water cluster around it. First, a reaction of the monoprotonated system, Ph–N(OH)H + H_3_O^+^(H_2_O)*_n_* (*n* = 4 and 14) was examined. However, the rate-determining transition states involving proton transfers were calculated to have much larger activation energies than the experimental one. Second, a reaction of the diprotonated system, Ph–N(OH)H + (H_3_O^+^)_2_(H_2_O)_13_, was traced. An activation energy similar to the experimental one was obtained. A new mechanism of the rearrangement including the aniline dication-like transition state was proposed.

## Introduction

**Scheme 1:**
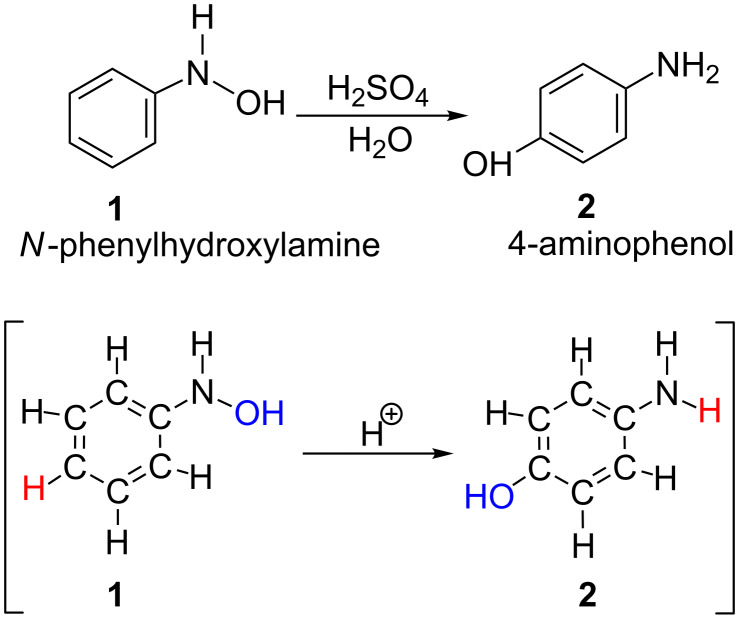
The Bamberger rearrangement. In the square bracket, the apparent exchange of H and OH is shown.

The fundamental Bamberger rearrangement is defined in Scheme 1 [1–2].

**Scheme 2:**
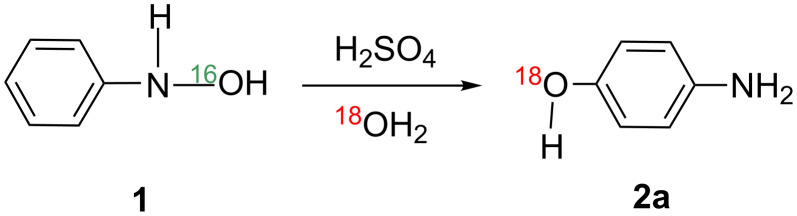
The reaction occurs through the intermolecular rearrangement, on the basis that treatment of **1** in H_2_[^18^O]H_2_SO_4_ provides an [^18^O]-incorporated *p*-aminophenol, **2a**.

In the aqueous sulfuric acid, 4-aminophenol was afforded exclusively by the rearrangement. On the other hand, the 2- and 4-chloro-amino derivatives were afforded when hydrochloric acid was used. In spite of the classic and well-known reaction, the mechanism of the Bamberger rearrangement is still unclear. Heller et al. suggested that an S_N_1 mechanism is more likely, but the S_N_2 one cannot be ruled out [3]. The reaction was proven to occur via the intermolecular rearrangement by the ^18^O exchange in Scheme 2 [4].

**Scheme 3:**
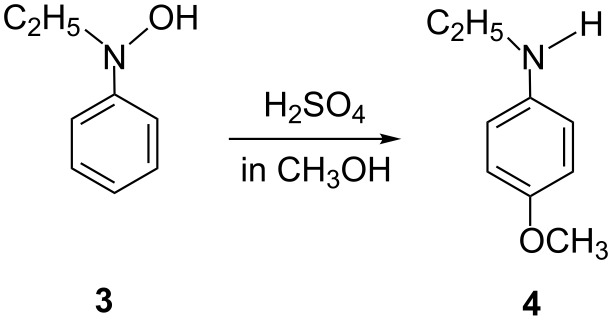
A reaction of *N*-ethyl-*N*-phenylhydroxylamine, which demonstrates that the Bamberger rearrangement does not take the route of the direct [1,5]-OH shift.

The intermolecular nature was also proven by a rearrangement of *N*-ethyl-*N*-phenylhydroxylamine, Et–N(OH)–Ph **3**, in methanol leading to *p*-(ethylamino)anisole **4** (Scheme 3) [5].

**Scheme 4:**
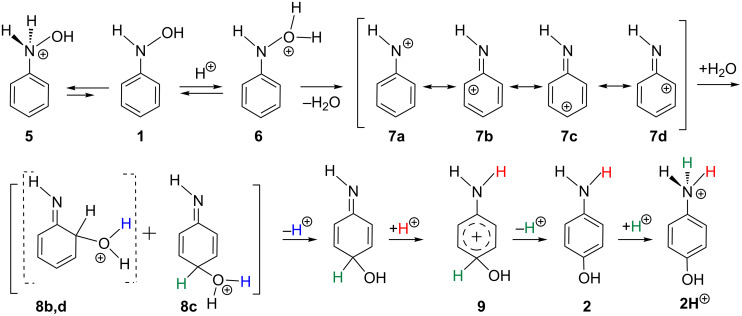
A mechanism involving the nitrenium-ion intermediate **7**. **8a** is equal to **6**.

Through the kinetic measurement, the rearrangement was claimed to occur by an S_N_1 mechanism [6]. Also, it was reported that the elimination of water from Ar–N^+^H_2_OH is rate determining and a diprotonated species, Ar–N^+^H_2_OH_2_^+^, contributes significantly to the observed reaction rate at the acid-catalyst concentration [H_2_SO_4_] > 0.50 mol/L. The activation energy of the rearrangement in Scheme 1 was measured to be 24.8 kcal/mol. The S_N_1 mechanism suggested by Heller et al. [3] involves a nitrenium ion **7** as shown in Scheme 4.

In Scheme 4, a water molecule is taken off from the O-protonated form **6**, which leads to the nitrenium ion **7**. To the para position of the ion H_2_O is added, and the subsequent proton removal and attack give the product **2**H^+^. While the N-protonated species **5** appears to be more favorable than the O-protonated one **6**, the former has been regarded as not being an intermediate for the reaction progress. While the mechanism in Scheme 4 appears to be established, there is a significant question as to "…why in some cases, for example where the nucleophile is water, only the 4-isomer product is formed from phenylhydroxylamine, whereas in other cases, e.g., when chloride ion is present, both 2- and 4-chloro isomers are formed" [7]. This is a natural question in light of the ortho–para orientation onto the cationic phenyl ring.

So far, there have been no theoretical studies of the rearrangement, and in this study DFT calculations were carried out to address the following three unsolved issues:

(a) Is the nitrenium ion **7** a plausible intermediate?

(b) How does the N-protonated species (**5** in Scheme 4) participate in the rearrangement? The reverse route (**5** → **1** + H^+^ needed for the reaction progress) seems to be unlikely.

(c) Why is the para-product afforded exclusively in the H_2_SO_4_ aqueous media?

It will be shown that the size of the hydrogen-bond network of water clusters in the diprotonated system controls the reactivity of the rearrangement.

## Theoretical calculations

The reacting systems were investigated by density functional theory (DFT) calculations. The B3LYP [8–9] method was used to trace the reaction path. The basis sets employed were 6-31G(d) and 6-311+G(d,p), where the latter was adopted for the key (OH transfer) steps. For the Cl-containing model, 6-31(+)G(d) was used where the diffuse sp function is only the chlorine atom.

Transition states (TSs) were sought first by partial optimizations at bond-interchange regions. Second, by the use of Hessian matrices, TS geometries were optimized. They were characterized by vibrational analyses, which checked whether the obtained geometries have single imaginary frequencies (ν^≠^s). From TSs, reaction paths were traced by the intrinsic reaction coordinate (IRC) method [10–11] to obtain the energy-minimized geometries.

Relative energies (Δ*E*s) were obtained by single-point calculations of the B3LYP/6-311+G(d,p) method (SCRF = PCM, solvent = water) [12–14] on the B3LYP/6-31G(d) and B3LYP/6-311+G(d,p) geometries and their zero-point vibrational energies (ZPEs).

All the calculations were carried out by using the Gaussian 09 [15] program package. The computations were performed at the Research Center for Computational Science, Okazaki, Japan.

## Results and Discussion

### The monoprotonated reacting system

First, the possibility of the nitrenium intermediate **7** was examined by the use of a model of **7** + (H_2_O)_18_. Figure S1, Supporting Information File 1 exhibits the assumed initial geometry (a) and B3LYP/6-31G(d) and B3LYP/6-311+G(d,p) optimized ones (b).

**Scheme 5:**
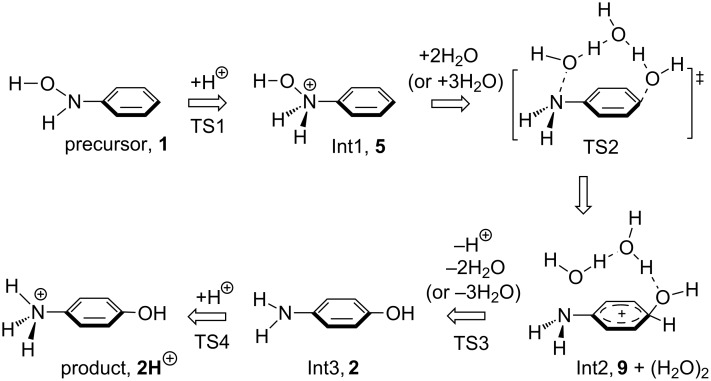
A reaction scheme of the OH rearrangement containing one proton. Int is an intermediate. Species, **1**, **2**, **5**, **2H**^+^ and **9**, are defined in Scheme 4.

By both computational methods, the nitrenium ion disappeared, and the **7** + (H_2_O)_18_ model was converted to a geometry of *o*-OH imine and H_3_O^+^(H_2_O)_16_. The nitrenium ion was calculated to be inevitably subject to the nucleophilic attack of OH_2_. This attack proceeds energetically downhill without the transition state, and the nitrenium ion was absent. Thus, there should be a mechanism other than that in Scheme 4. Second, a reaction model (Scheme 5) involving proton transfers was investigated.

In the scheme, the N-protonated form Int1 (i.e., **5** in Scheme 4) undergoes the transfer via the water dimer (or trimer) at TS2. The transfer pattern is drawn for the rearrangement to the para position (apparently, a [1,5]-OH shift); that to the ortho one would be a [1,3]-OH shift. Figure S2, Supporting Information File 1 shows the three obtained TS2 geometries. Their presence demonstrates that paths involving [1,5]- and [1,3]-shifts with more water molecules than those in Figure S2 should be examined on an equal footing. Figure S3, Supporting Information File 1 shows the path calculated by a model, called here model(I), composed of Ph–NH(OH) and H_3_O^+^(H_2_O)_4_.

The geometric changes expected in Scheme 5 were obtained: Precursor(I) → TS1(I) → Int1(I) → TS2(I, 2H_2_O) → Int2(I), Int2'(I) → TS3(I) → Int3(I) → Product(I). Here, Int2(I) and Int2'(I) are isomers where the positions of water clusters are different. TS4(I) leading to the protonated *p*-aminophenol could not be obtained, probably owing to the limited size of the reaction system. TS2(I, 3H_2_O) and TS2(I, [1,3]-shift) were also obtained and are shown at the end of Figure S3. Here, TS2(I, 2H_2_O), TS2(I, 3H_2_O) and TS2(I, [1,3]-shift) correspond to three TS2s in Figure S2, respectively.

**Figure 1:**
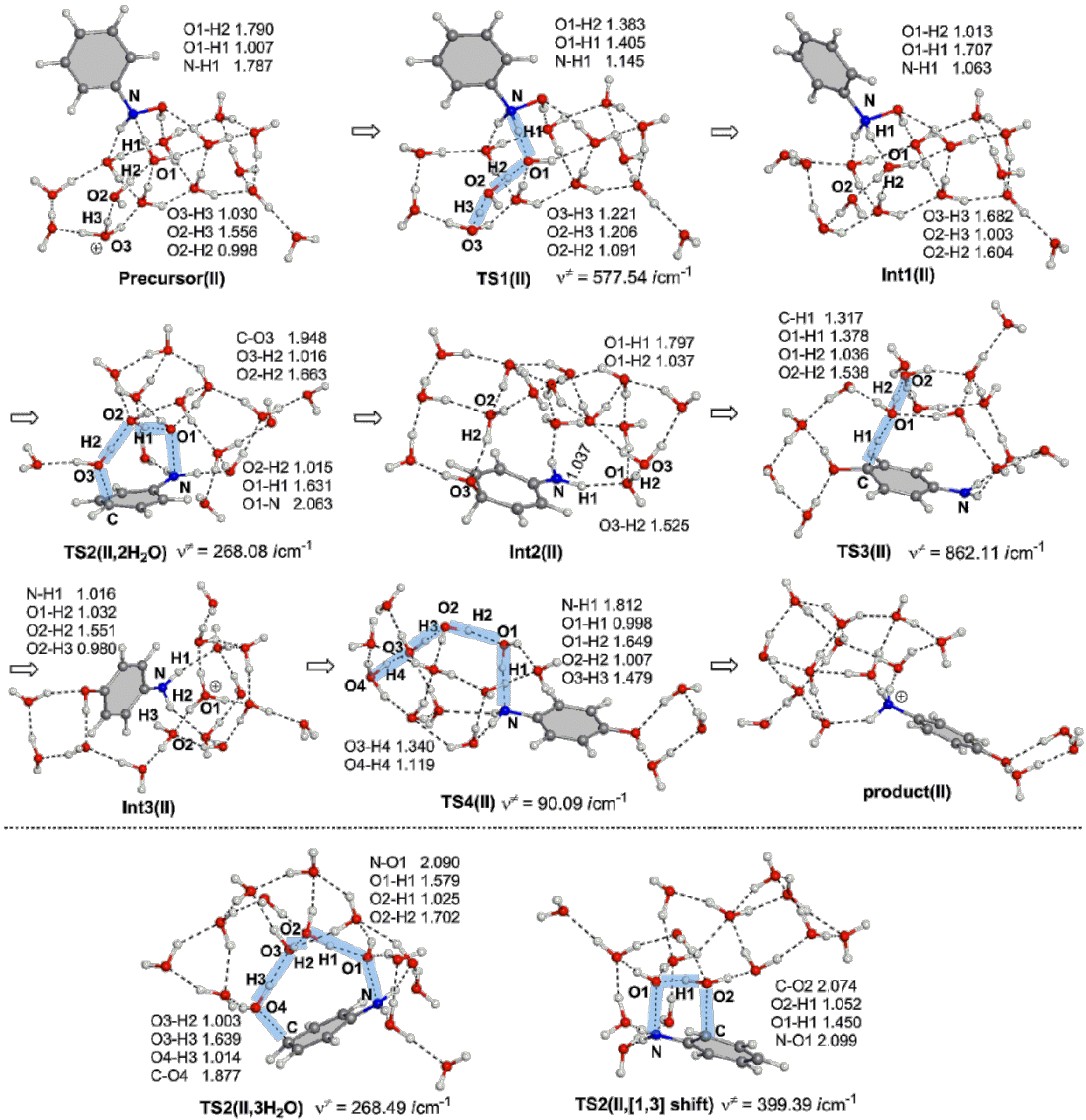
Geometric changes in the reaction of model II, (HO)HN–C_6_H_5_ + H_3_O^+^(H_2_O)_14_ → H_3_N^+^–C_6_H_4_–OH + (H_2_O)_15_.

**Figure 2:**
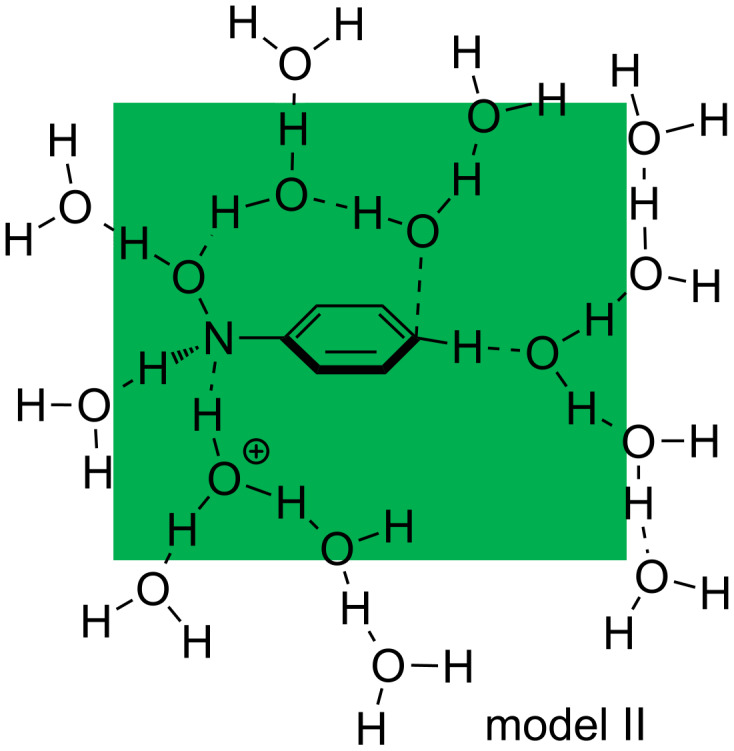
An assumed reaction system composed of Ph–NHOH and H_3_O^+^(H_2_O)_14_. The green area represents the reaction region of TS2. To protons of H_3_O^+^ and H_2_O in the area, catalytic water molecules are linked in the O─H•••OH_2_ hydrogen-bond pattern.

Figure 1 shows the path calculated by a further extended reaction system, Ph–NH(OH) and H_3_O^+^(H_2_O)_14_, called here model(II). This is constructed on the basis of the hydrogen-bond network depicted in Figure 2. Again, the geometric changes expected in Scheme 5 were obtained: Precursor(II) → TS1(II) → Int1(II) → TS2(II, 2H_2_O) → Int2(II) → TS3(II) → Int3(II) → TS4(II) → Product(II) along with TS(II, 3H_2_O) and TS2(II, [1,3]-shift). Thus, the reaction pattern predicted in Scheme 5 holds for the system Ph–NH(OH) and H_3_O^+^(H_2_O)*_n_* (*n* = 4 and 14).

**Figure 3:**
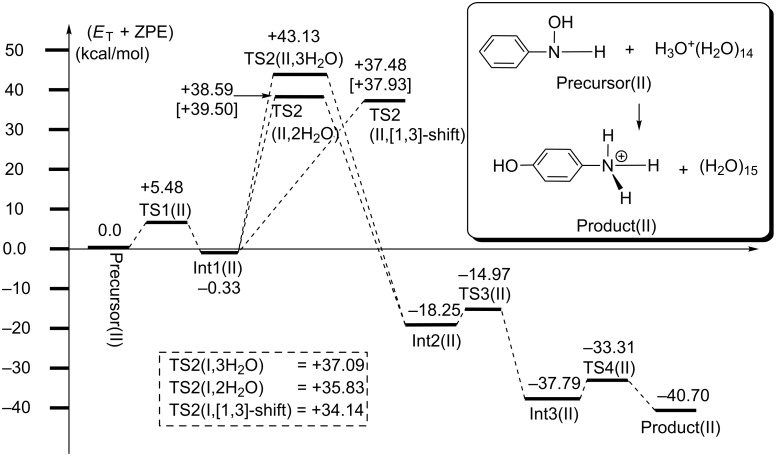
Energy changes (in kcal/mol) of Δ(*E*+ZPE) by B3LYP/6-311+G(d,p) SCRF = PCM//B3LYP/6-31G(d) and by [B3LYP/6-311+G(d,p) SCRF = PCM//B3LYP/6-311+G(d,p) at TS2] of model II. The corresponding geometries are shown in Figure 1. In the broken box, three activation energies of TS2(I, 2H_2_O), TS2(I, 3H_2_O) and TS2(I, [1,3]-shift) of Figure S3 are exhibited.

Figure 3 shows the energy change of the reaction in Figure 1. The reaction was calculated to be very exothermic (= −40.70 kcal/mol at Product(II)). However, the activation energies of the three TS2s, +38.59, +37.48 and +43.13 kcal/mol, of the high-energy steps are much larger than the experimental one, +24.8 kcal/mol [6]. Also, the three activation energies of model I in the broken box are large, +34.14, +35.83 and 37.09 kcal/mol. Thus, although reasonable geometric changes were obtained in Figure 1, these large energies demonstrate that the monoprotonated reaction is unlikely.

### Diprotonated reacting systems

**Scheme 6:**
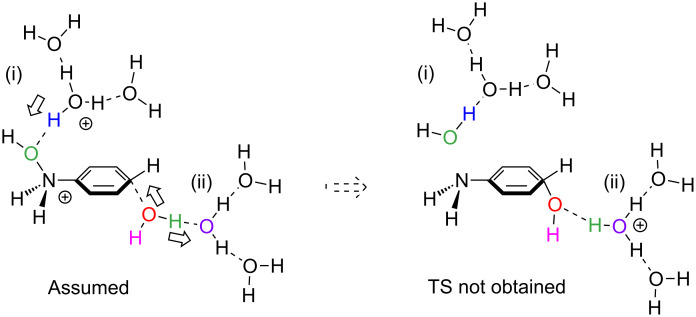
A trans-type bond interchange was assumed. But, the reaction path could not be obtained. The group (i) works as a proton donor and the group (ii) acts as an acceptor.

**Scheme 7:**
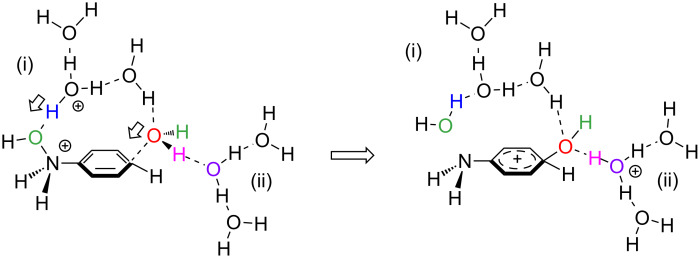
An alternative model for the OH [1,5]-rearrangement in the dication system.

The large activation energies of TS2s in the monoprotonated systems would arise from the poor proton-donating strength to the oxygen of the N–O bond in the model of Scheme 5. In order to enhance the donation, a dication system was constructed at the left of Scheme 6. However, the bond-interchange transition state could not be obtained in spite of many attempts. Attempts including more water molecules also failed. An alternative model was considered and is shown in Scheme 7. This model was constructed in light of the results of preliminary calculations shown in Figure S4 and Figure S5, Supporting Information File 1.

Figure S4a shows that a TS geometry was successfully obtained in a model in which one H_2_O molecule is subtracted from that in Scheme 7. However, when the size of the water cluster is enlarged, the TS structure cannot be obtained, as shown in Figure S4b. On the other hand, a TS geometry following Scheme 7 could be obtained as shown in Figure S5, Supporting Information File 1. These results demonstrate that not H_3_O^+^(H_2_O) (in Figure S4) but H_3_O^+^(H_2_O)_2_ (in Figure S5) should participate in the reaction center.

**Figure 4:**
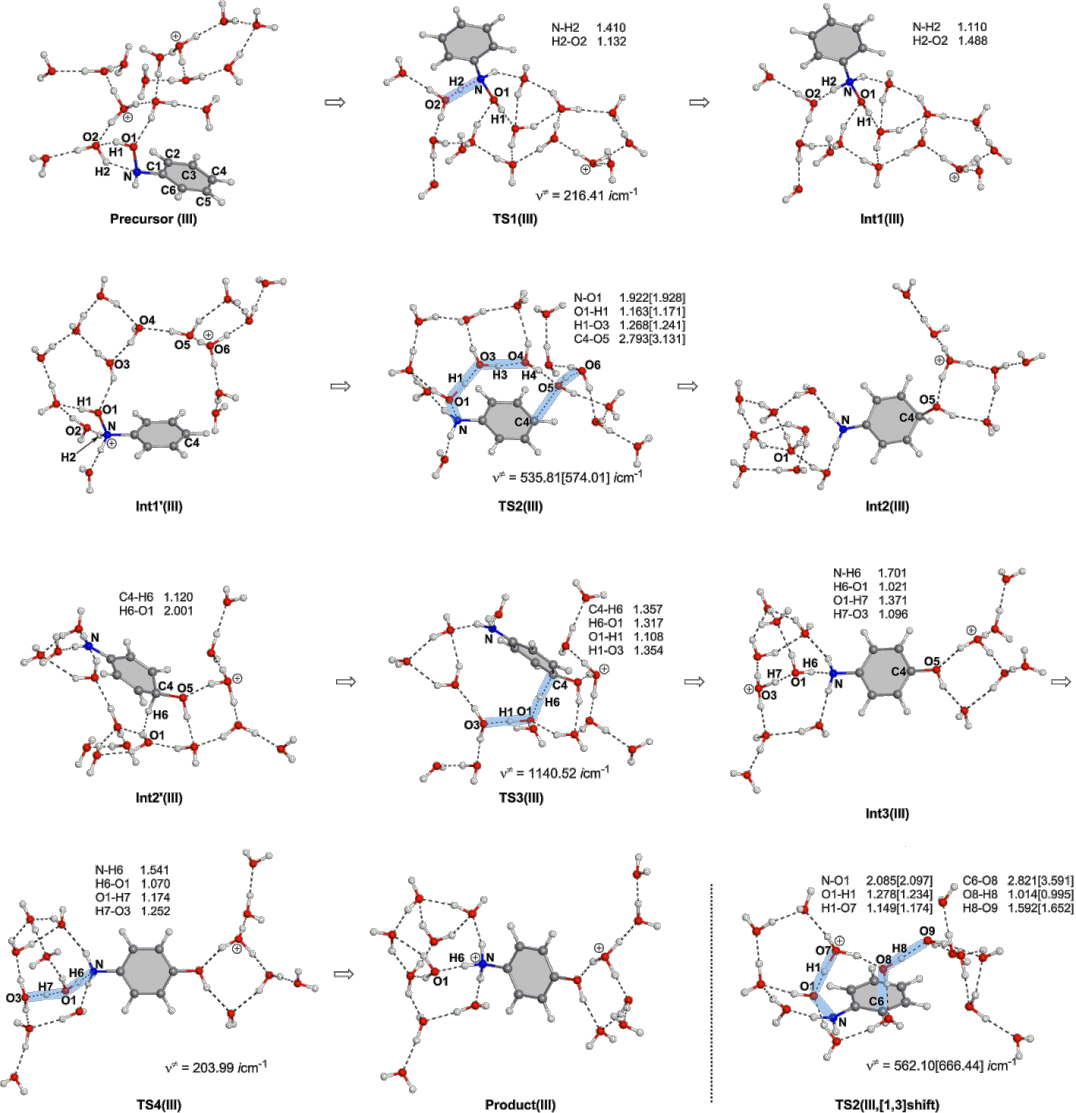
Geometric changes in the reaction of model III, (HO)HN–C_6_H_5_ + (H_3_O^+^)_2_(H_2_O)_13_ → H_3_N^+^–C_6_H_4_–OH + (H_3_O^+^)(H_2_O)_14_.

On the basis of the result in Figure S5, paths in a reaction of (HO)HN–C_6_H_5_ + (H_3_O^+^)_2_(H_2_O)_13_ → H_3_N^+^–C_6_H_4_–OH + (H_3_O^+^)(H_2_O)_14_ were investigated and are shown in Figure 4. This system is called here model III and is isoelectronic with that in Figure 1.

Geometric changes similar to those in Figure 1 were obtained: Precursor(III) → TS1(III) → Int1(III), Int1'(III) → TS2(III) → Int2(III), Int2'(III) → TS3(III) → Int3(III) → TS4(III) → Product(III) along with TS2(III, [1,3]-shift). Geometries of TS2(III) and TS2(III, [1,3]-shift) are like those of the aniline dication and (H_2_O)_16_.

**Figure 5:**
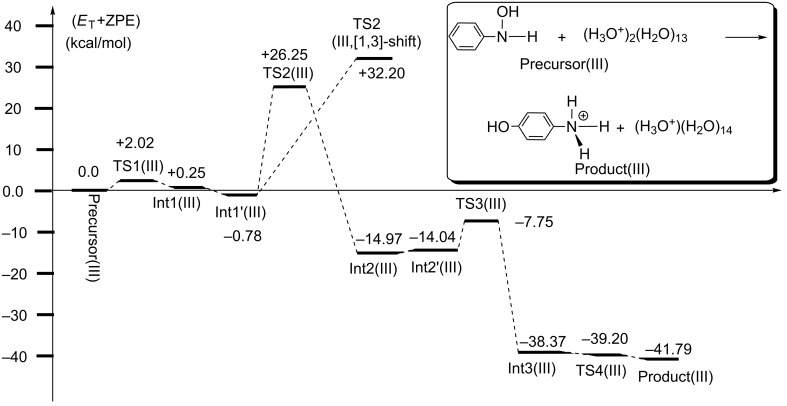
Energy changes (in kcal/mol) of model III. The corresponding geometries are shown in Figure 4. The apparent small reversal of energies of Int3(III) and TS4(III) comes from the splicing method, B3LYP/6-311+G(d,p) SCRF = PCM//B3LYP/6-31G(d), Et(B3LYP/6-31G(d)) of Int3(III) = −1509.9900126 Hartree and Et(B3LYP/6-31G(d)) of TS4(III) = −1509.9895045 Hartree.

Figure 5 shows the energy change of the reaction in Figure 4. The rate-determining step is TS2, and TS2(III,[1,3]-shift) = +32.20 kcal/mol is much larger than TS2(III) = +26.25 kcal/mol. The latter value is close to the experimental one, +24.8 kcal/mol [6], and the superiority of the [1,5]-OH shift over the [1,3]-OH one is clearly indicated. Thus, the dication system may be subject to the Bamberger rearrangement in the para-orientation, which is in line with the experimental suggestion [6].

**Figure 6:**
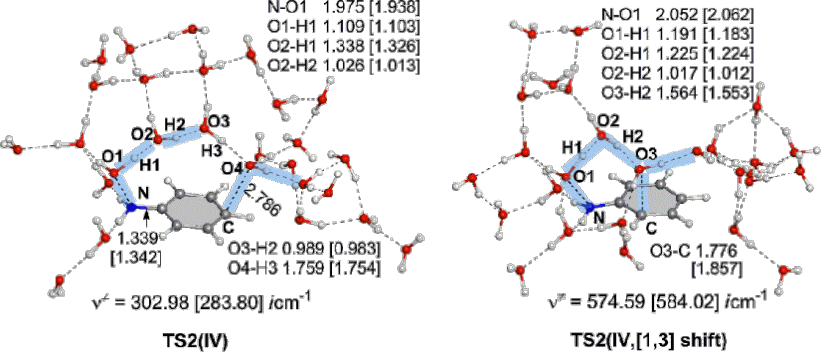
TS2(IV) and TS2(IV, [1,3]-shift) in the reaction (model IV), Ph–NH(OH) + (H_3_O^+^)_2_(H_2_O)_24_ → HO–C_6_H_4_–NH_3_^+^ + H_3_O^+^(H_2_O)_25_.

The reaction pattern exhibited in Scheme 7 was examined further by a large system composed of Ph–NH(OH) + (H_3_O^+^)_2_(H_2_O)_24_ with the molecular formula of the system, C_6_H_61_NO_27_^2+^. This is called here model IV. Geometries of TS2(IV) and TS2(IV, [1,3]-shift) are shown in Figure 6. They are similar to those of TS2(III) and TS2(III, [1,3]-shift) in Figure 4, respectively. Again, the aniline dication-like structures were obtained. The proton-transfer pattern depicted in Scheme 7 was confirmed. As for the activation energies of TS2(IV), Δ(*E*_T_ + ZPE) = +27.58 kcal/mol by B3LYP/6-311+G(d,p) SCRF = PCM//B3LYP/6-31G(d) and {+26.04 by B3LYP/6-311+G(d,p) SCRF = PCM//B3LYP/6-311+G(d,p)} are close to the experimental one (+24.8). These are much smaller than +36.25 kcal/mol and {+35.28} of TS2(IV, [1,3]-shift), respectively. Thus, the calculated results showed that the para-orientation of the rearrangement is superior to the ortho one.

### The monoprotonated reacting system with Cl^−^

**Figure 7:**
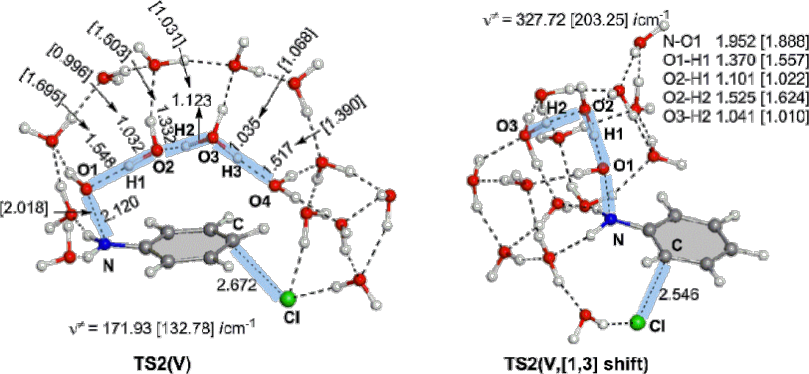
TS2(V) and TS2(V, [1,3]-shift) in the reaction (model V), Ph–NH(OH) + (H_3_O^+^)_2_(H_2_O)_13_ + Cl^−^ → *o*- and *p*-Cl–C_6_H_4_–NH_3_^+^ + (H_2_O)_16_.

In the Introduction, the experimental result that the rearrangement gave the 2- and 4-chloro-amino derivatives in hydrochloric acid was cited [1]. The nucleophile Cl^−^ would be free from the hydrogen-bond constraint depicted in Scheme 7. Then, such less sterically congested trans substitution as that shown in Scheme 6 becomes feasible. By the use of a model (called here model V) of Ph–NH(OH) + (H_3_O^+^)_2_(H_2_O)_13_ + Cl^−^, the trans-type substitution paths were traced. In fact, TS2(V) and TS2(V, [1,3]-shift) geometries were obtained and are shown in Figure 7. Their activation energies were calculated to be +28.52 kca/mol and +29.57 kcal/mol relative to the energy of Int1(V), respectively. These similar values indicate that both 2- and 4-Cl-substituted anilines may be formed almost equally according to the normal ortho- and para-orientation.

## Conclusion

In this work, the Bamberger rearrangement was studied by means of DFT calculations. In the Introduction, three questions (a), (b), and (c) were raised:

1. The nitrenium ion **7** was calculated to be absent. It cannot intervene owing to the high nucleophilicity of the water cluster.
2. The N-protonated substrate (**5** in Scheme 4) is in the reaction route. By the protonation, the N–O bond becomes directed to the π space of the phenyl ring. The direction is fit for the subsequent bond interchange of TS2 in the diprotonated system.
3. Without good nucleophiles such as Cl^−^, a constrained hydrogen-bond network shown in Scheme 7 may give the OH shift via bond interchanges. The ortho-position is too close to the N–O bond and is not fit for the constrained network.

**Scheme 8:**
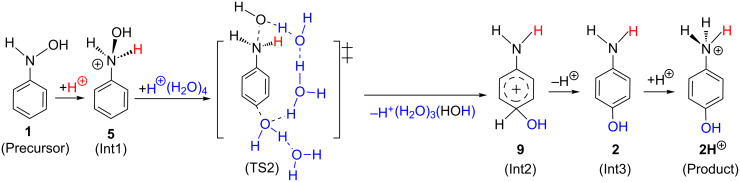
A mechanism of the Bamberger rearrangement based on the present results. **1**, **2**, **2H**^+^, **5** and **9** are defined in Scheme 4. In parentheses, our notations such as (Precursor) and (Int1) are shown. TS2 is regarded as the complex of the aniline di-cation and (H_2_O)_5_ cluster.

On the basis of the calculated results, Scheme 4 may be revised to Scheme 8.

## Supporting Information

File 1 Figures S1–S5, Cartesian coordinates of TS geometries.
