# Supplementary material for: An aniline dication-like transition state in the Bamberger rearrangement
Source: Beilstein J Org Chem. 2013 Jun 3;9:1073–82. doi: 10.3762/bjoc.9.119 (PMC3678851; doi:10.3762/bjoc.9.119)
Supplement: File 1 — Figures S1–S5, Cartesian coordinates of TS geometries. [file Beilstein_J_Org_Chem-09-1073-s001.pdf]

Supporting Information

for

An aniline dication-like transition state in the Bamberger rearrangement

Shinichi Yamabe*, Guixiang Zeng, Wei Guan and Shigeyoshi Sakaki

Address: Fukui Institute for Fundamental Chemistry, Kyoto University, Takano-Nishihiraki-cho 34-4, Sakyo-ku, Kyoto 606-8103, JAPAN. Phone: +81-075-711-7907.

E-mail: yamabes@fukui.kyoto-u.ac.jp.

* Corresponding author

Figures S1–S5, Cartesian coordinates of TS geometries

I. **Figure S1:** (a) An assumed model of Ph–NH^+ (nitrenium ion) and $(H_2O)_{18}$. (b) The result of geometry optimizations. The Ph–$NH^+(H_2O)_{18}$ initial geometry is converted to that of *o*-HO–C_6H_5=N–H and $H_3O^+(H_2O)_{16}$.

II. **Figure S2:** Three transition-state geometries of the OH rearrangement through proton transfers in minimal models of Ph–$NH_2(OH)^+$ and $(H_2O)_2$ [or $(H_2O)_3$]. Distances are in Å. Along blue zones, formation or scission of covalent bonds occurs. Values without and with square brackets are of B3LYP/6-31G(d) and B3LYP/6-311+G(d,p), respectively.

III. **Figure S3:** Geometric changes in the reaction I, Ph–NH(OH) + $H_3O^+(H_2O)_4$ → HO–C_6H_4–NH_3^+ + $(H_2O)_5$.

IV. **Figure S4a:** A TS geometry of conversion, (HO)H_2N^+–C_6H_5 + $H_3O^+(H_2O)_5$ → H_2N–C_6H_5–OH^+ + $H_3O^+(H_2O)_5$.

V. **Figure S4b:** A failure in the TS optimization based on the geometry of Figure S4a.

VI. **Figure S5:** A TS geometry following Scheme 7.

VII. Cartesian coordinates of TS geometries.

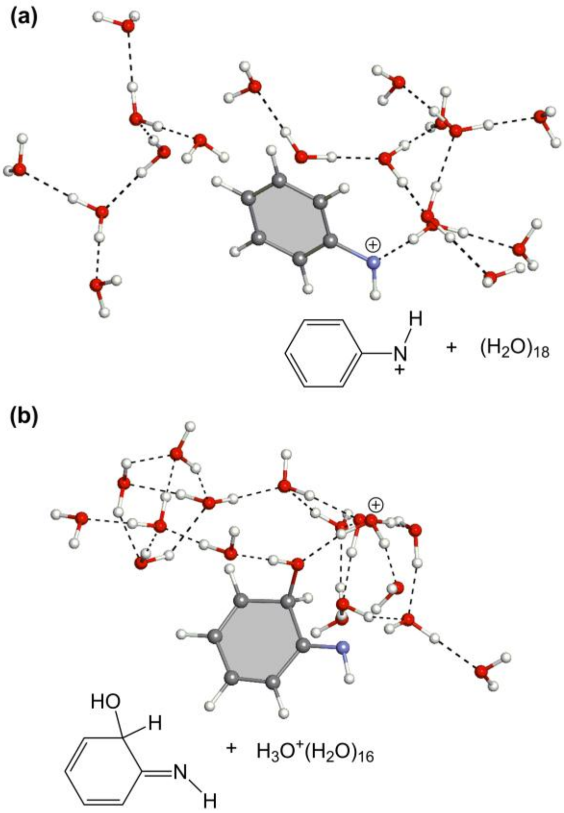

Figure S1: (a) An assumed model of Ph–NH$^+$ (nitrenium ion) and $(H_2O)_{18}$. (b) The result of geometry optimizations. The Ph–NH$^+(H_2O)_{18}$ initial geometry is converted to that of *o*-HO–C_6H_5=N–H and $H_3O^+(H_2O)_{16}$.

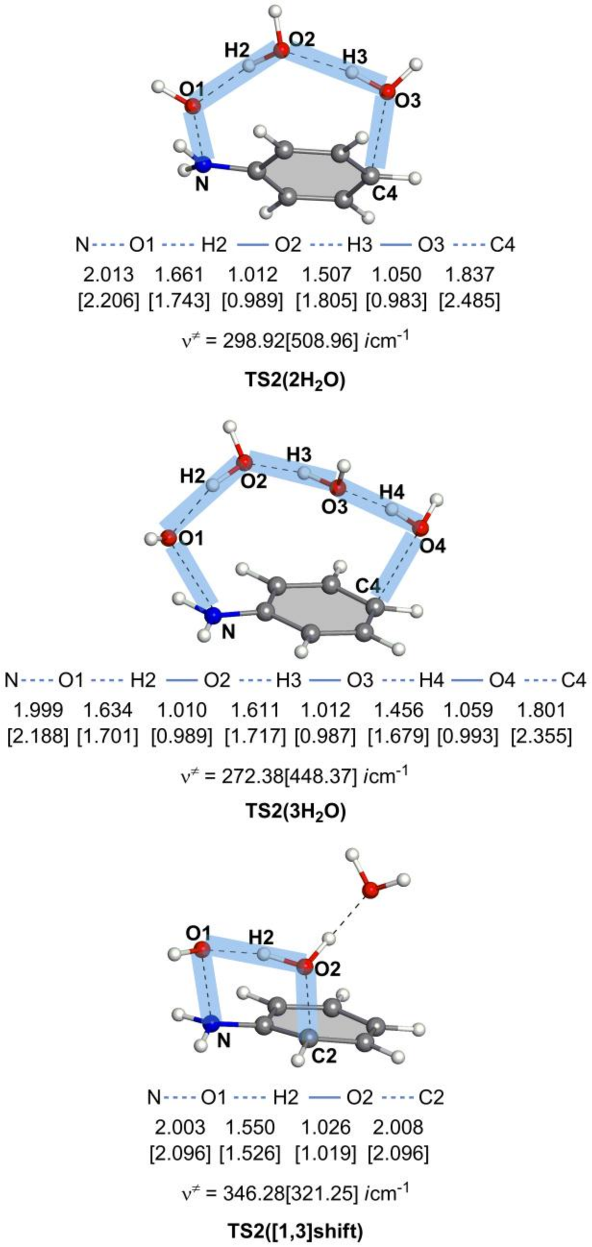

Figure S2: Three transition-state geometries of the OH rearrangement through proton transfers in minimal models of Ph–$NH_2(OH)^+$ and $(H_2O)_2$ [or $(H_2O)_3$]. Distances are in Å. Along blue zones, formation or scission of covalent bonds occurs. Values without and with square brackets are of B3LYP/6-31G(d) and B3LYP/6-311+G(d,p), respectively.

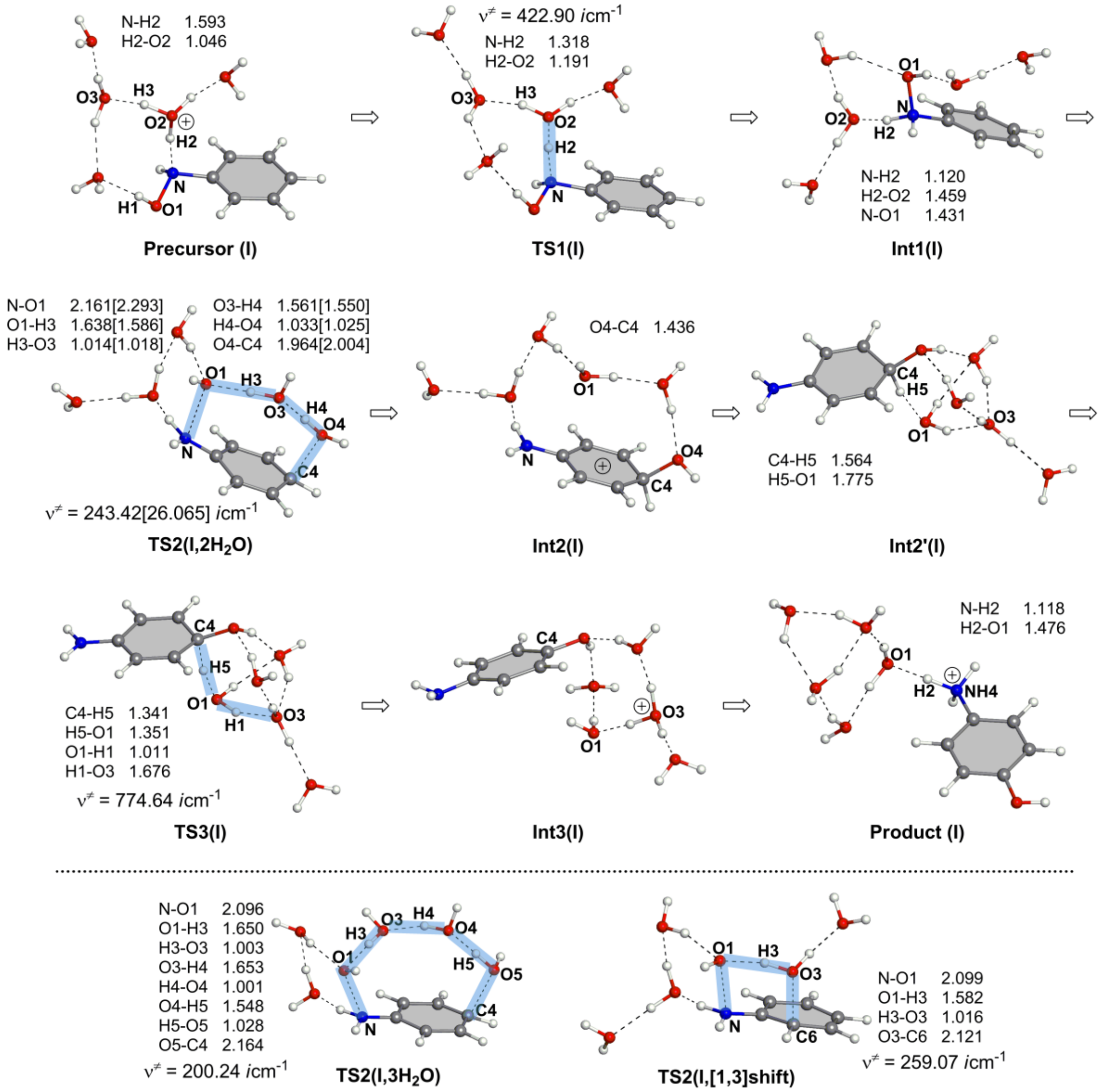

Figure S3: Geometric changes in the reaction I, Ph–NH(OH) + $H_3O^+(H_2O)_4$ → $HO–C_6H_4–NH_3^+$ + $(H_2O)_5$.

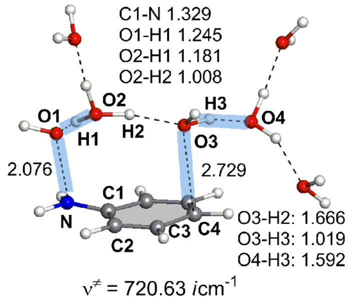

Figure S4a: A TS geometry of conversion, $(HO)H_2N^+–C_6H_5$ + $H_3O^+(H_2O)_5$ → $H_2N–C_6H_5–OH^+$ + $H_3O^+(H_2O)_5$.

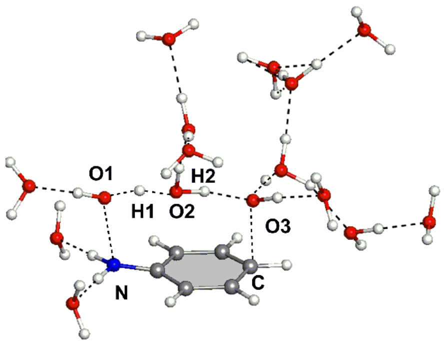

Figure S4b: A failure in the TS optimization based on the geometry of Figure S4a.

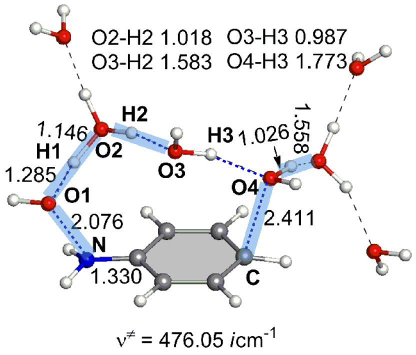

Figure S5: A TS geometry following Scheme 7.

==

Cartesian coordinates of TS geometries

==

[1] TS geometries in Figure 1

== TS1(II) of the proton transfer TS by B3LYP/6-31G(d)==

Stoichiometry C6H38NO16(1+)

bamb1ts0.log

Standard orientation:

Center Number	Atomic Number	Atomic Type	Coordinates (Angstroms) X	Y	Z
1	6	0	-2.702446	-1.762134	-0.218795
2	7	0	-1.472075	-0.980455	-0.191638
3	6	0	-2.805717	-2.863379	-1.066757
4	6	0	-3.773625	-1.328739	0.561397
5	6	0	-4.015595	-3.553411	-1.123314
6	6	0	-4.982697	-2.023127	0.481261
7	6	0	-5.104239	-3.133723	-0.354365
8	1	0	-1.343429	-0.241717	-1.057660
9	1	0	-1.951652	-3.182749	-1.653565
10	1	0	-3.680054	-0.461428	1.211525
11	1	0	-4.108237	-4.418707	-1.772739
12	1	0	-5.826242	-1.694876	1.080863
13	1	0	-6.045147	-3.673165	-0.407074
14	8	0	-0.302065	-1.814921	-0.237094
15	1	0	0.280436	-1.507468	0.534339
16	1	0	-1.413873	-0.396767	0.677024
17	1	0	3.421932	-3.477795	1.784065
18	8	0	3.489762	-2.539299	1.549731
19	1	0	3.643402	-2.497457	0.568973
20	8	0	-0.961401	0.823105	-1.892217
21	1	0	-0.295989	0.538216	-2.540408
22	1	0	-0.279748	1.774694	-0.771254
23	8	0	1.629315	-0.701054	-2.035385
24	1	0	0.937428	-1.201119	-1.553076
25	1	0	2.427948	-1.268204	-1.975176
26	8	0	0.005872	2.371310	-0.002123
27	1	0	0.985258	2.273151	0.017588
28	1	0	-1.063743	3.698225	-0.389455
29	8	0	-1.894003	4.121642	-0.738270
30	1	0	-2.278550	3.394077	-1.641073
31	1	0	-2.571103	4.096903	0.017096
32	8	0	2.363753	1.023226	-0.066606
33	1	0	3.352272	1.015566	-0.071864
34	1	0	2.108186	0.509114	-0.880800
35	8	0	3.980522	-1.962963	-1.081523
36	1	0	4.588584	-2.515437	-1.597560
37	1	0	4.476760	-1.114249	-0.875196
38	8	0	5.005008	0.321809	-0.197762
39	1	0	5.266877	0.064080	0.729264
40	1	0	5.759723	0.831103	-0.581402
41	8	0	7.190601	1.691647	-1.165222
42	1	0	7.396565	2.532600	-0.728639
43	1	0	7.281591	1.873115	-2.113256
44	8	0	1.304166	-0.813508	1.596609

45	1	0	2.006063	-1.467198	1.820464
46	1	0	1.774949	-0.105482	1.067572
47	8	0	-0.919162	0.756343	1.940116
48	1	0	-0.545808	1.457844	1.332088
49	1	0	-0.138681	0.277549	2.290472
50	8	0	-3.649294	3.962036	1.196093
51	1	0	-3.601838	3.140988	1.749381
52	1	0	-3.667793	4.705860	1.815495
53	8	0	-3.389553	1.649273	2.624247
54	1	0	-3.684062	1.579973	3.544288
55	1	0	-2.455720	1.335320	2.605186
56	8	0	-2.577777	2.578972	-2.479450
57	1	0	-2.472166	2.954987	-3.367841
58	1	0	-1.884009	1.747345	-2.349054
59	8	0	5.373982	-0.635107	2.281024
60	1	0	4.780106	-1.416711	2.229046
61	1	0	6.232391	-0.970000	2.579924

SCF Done: E(RB+HF-LYP) = -1509.64047955 A.U. after   2 cycles

Harmonic frequencies (cm**-1), IR intensities (KM/Mole)

	1	2	3
	A	A	A
Frequencies --	-577.5370	19.7441	23.2186
Red. masses --	1.3924	5.2709	5.2703
Frc consts --	0.2736	0.0012	0.0017
IR Inten --	2027.0993	0.1539	0.7893

Zero-point correction= 0.513787 (a.u.)
Thermal correction to Energy= 0.555178
Thermal correction to Enthalpy= 0.556122
Thermal correction to Gibbs Free Energy= 0.437091
Sum of electronic and zero-point Energies= -1509.126693
Sum of electronic and thermal Energies= -1509.085301
Sum of electronic and thermal Enthalpies= -1509.084357
Sum of electronic and thermal Free Energies= -1509.203388

	E (Thermal)	CV	S
	KCal/Mol	Cal/Mol-Kelvin	Cal/Mol-Kelvin
Total	348.380	146.735	250.522

== TS2(II,2H2O) by B3LYP/6-31G(d)==

Stoichiometry   C6H38NO16(1+)
bamb25a4.chk
Standard orientation:

Center Number	Atomic Number	Atomic Type	Coordinates (Angstroms) X	Y	Z
1	6	0	-1.341980	0.485621	1.861835
2	7	0	-0.206736	-0.144845	2.106358
3	6	0	-1.315405	1.821093	1.315337
4	6	0	-2.597013	-0.220117	1.961297
5	6	0	-2.436557	2.353692	0.766026
6	6	0	-3.714269	0.314521	1.402496
7	6	0	-3.631816	1.550520	0.654069

8	1	0	0.725439	0.376512	2.002133
9	1	0	-0.390128	2.383173	1.349698
10	1	0	-2.606726	-1.184833	2.461138
11	1	0	-2.421249	3.353907	0.347418
12	1	0	-4.668370	-0.200019	1.449133
13	1	0	-4.559788	2.063777	0.428526
14	8	0	0.376909	-1.112390	0.379790
15	1	0	0.230997	-2.060656	0.580481
16	1	0	-0.239587	-1.083518	2.549427
17	1	0	-0.793523	-0.713591	-0.683834
18	8	0	-1.350787	-0.540462	-1.514921
19	1	0	-0.675605	-0.032948	-2.075081
20	1	0	-2.698248	0.407988	-1.291591
21	8	0	-3.533407	0.980010	-1.205513
22	1	0	-4.312930	0.378440	-1.317372
23	8	0	1.962617	1.353212	1.705430
24	1	0	1.721098	1.837431	0.876844
25	1	0	2.799596	0.903040	1.439262
26	8	0	1.385833	2.578632	-0.716974
27	1	0	2.339031	2.736496	-0.973227
28	1	0	0.938023	3.450846	-0.673339
29	8	0	4.141136	0.382380	0.298119
30	1	0	3.672964	-0.269333	-0.287247
31	1	0	4.978275	-0.036675	0.603275
32	8	0	-0.716263	-2.756212	2.939558
33	1	0	-0.388035	-3.236982	3.713773
34	1	0	-0.538824	-3.335710	2.161694
35	8	0	6.535665	-0.749952	1.160066
36	1	0	7.308786	-0.377128	0.708666
37	1	0	6.705551	-0.598901	2.102797
38	8	0	2.434204	-1.036781	-1.230652
39	1	0	2.460496	-1.983407	-1.484504
40	1	0	1.708669	-1.021128	-0.527698
41	8	0	-0.195601	-3.966987	0.507005
42	1	0	-0.806879	-3.825819	-0.246814
43	1	0	0.656543	-4.124723	0.047757
44	8	0	4.005127	2.613002	-1.256932
45	1	0	4.210017	1.826280	-0.686456
46	1	0	4.148665	2.303819	-2.164426
47	8	0	-0.181826	4.894922	-0.414318
48	1	0	0.212248	5.606184	0.115152
49	1	0	-0.421541	5.316729	-1.254488
50	8	0	0.703887	0.680253	-2.646859
51	1	0	1.396193	0.040984	-2.359311
52	1	0	0.851377	1.440138	-2.034967
53	1	0	-1.203010	-2.156929	-1.976705
54	8	0	-1.012489	-3.140377	-2.054250
55	1	0	-1.557059	-3.469388	-2.784925
56	8	0	-5.861798	-0.478381	-1.238861
57	1	0	-6.580385	-0.047234	-1.728049
58	1	0	-5.905964	-1.410999	-1.500896
59	8	0	1.632139	-3.720036	-1.612937
60	1	0	0.757246	-3.497836	-2.017474
61	1	0	2.013244	-4.422721	-2.161650

SCF Done: E(RB+HF-LYP) = -1509.57395703 A.U. after 1 cycles
Convg =   0.5973D-08   -V/T = 2.0088

S**2 = 0.0000
Range of M.O.s used for correlation: 1 421

Harmonic frequencies (cm**-1), IR intensities (KM/Mole)

	1	2	3
	A	A	A
Frequencies --	-268.0831	20.5478	25.0174
Red. masses --	7.5352	5.0802	4.5520
Frc consts --	0.3191	0.0013	0.0017
IR Inten --	454.2135	1.3421	3.7414

Zero-point correction= 0.514093 (a.u.)
Thermal correction to Energy= 0.558492
Thermal correction to Enthalpy= 0.559436
Thermal correction to Gibbs Free Energy= 0.435218
Sum of electronic and zero-point Energies= -1509.059864
Sum of electronic and thermal Energies= -1509.015465
Sum of electronic and thermal Enthalpies= -1509.014521
Sum of electronic and thermal Free Energies= -1509.138739

	E (Thermal)	CV	S
	KCal/Mol	Cal/Mol-Kelvin	Cal/Mol-Kelvin
Total	350.459	154.203	261.440

==TS2(II,2H2O) by B3LYP/6-311+G(d,p)==

Stoichiometry C6H38NO16(1+) bamb25a4.log
Standard orientation:

Center Number	Atomic Number	Atomic Type	Coordinates (Angstroms) X	Y	Z
1	6	0	-1.288131	0.551466	1.922020
2	7	0	-0.187947	-0.134294	2.117592
3	6	0	-1.220138	1.874508	1.344182
4	6	0	-2.575463	-0.071362	2.129014
5	6	0	-2.339938	2.456636	0.858295
6	6	0	-3.688862	0.510509	1.626040
7	6	0	-3.583766	1.722946	0.845007
8	1	0	0.761434	0.310930	1.937134
9	1	0	-0.262119	2.375257	1.297233
10	1	0	-2.608671	-1.016492	2.658950
11	1	0	-2.308549	3.443122	0.414823
12	1	0	-4.664241	0.059889	1.760200
13	1	0	-4.491541	2.281753	0.660944
14	8	0	0.236971	-1.236483	0.318636
15	1	0	0.061702	-2.158321	0.563738
16	1	0	-0.245490	-1.070772	2.545106
17	1	0	-0.907132	-0.694476	-0.697928
18	8	0	-1.477636	-0.384807	-1.472404
19	1	0	-0.810363	0.133415	-1.996427
20	1	0	-2.804276	0.521546	-1.171318
21	8	0	-3.604073	1.121153	-1.010749
22	1	0	-4.428462	0.618719	-1.191046
23	8	0	2.093094	1.185679	1.603565
24	1	0	1.954558	1.830326	0.878247
25	1	0	2.881865	0.684062	1.312494
26	8	0	1.752505	2.807879	-0.680623

27	1	0	2.704939	2.798177	-0.935539
28	1	0	1.474495	3.741023	-0.659026
29	8	0	4.199954	-0.038429	0.177097
30	1	0	3.660501	-0.629581	-0.397197
31	1	0	4.952245	-0.552476	0.522417
32	8	0	-0.800170	-2.796877	2.933679
33	1	0	-0.638828	-3.319417	3.725570
34	1	0	-0.737207	-3.410775	2.173460
35	8	0	6.413510	-1.458593	1.220414
36	1	0	7.256224	-1.533505	0.760462
37	1	0	6.624652	-1.450411	2.159917
38	8	0	2.290993	-1.280231	-1.321785
39	1	0	2.241421	-2.183209	-1.673166
40	1	0	1.559290	-1.255827	-0.637266
41	8	0	-0.563713	-4.108440	0.467290
42	1	0	-1.179721	-3.906430	-0.256490
43	1	0	0.245456	-4.373819	0.001135
44	8	0	4.395372	2.384280	-1.116918
45	1	0	4.490773	1.502409	-0.694581
46	1	0	4.856978	2.341072	-1.959318
47	8	0	0.815050	5.491946	-0.545677
48	1	0	1.269348	6.118693	0.028437
49	1	0	0.630991	5.972720	-1.360252
50	8	0	0.699195	0.839842	-2.512407
51	1	0	1.358853	0.145785	-2.327974
52	1	0	0.984887	1.596385	-1.966341
53	1	0	-1.549646	-2.089935	-2.043709
54	8	0	-1.487823	-3.071508	-2.134274
55	1	0	-2.122031	-3.326687	-2.812043
56	8	0	-6.085596	0.005941	-1.476413
57	1	0	-6.643740	0.462973	-2.115835
58	1	0	-6.410655	-0.900050	-1.438218
59	8	0	1.186671	-3.969220	-1.889118
60	1	0	0.314575	-3.684894	-2.225133
61	1	0	1.506485	-4.646371	-2.494471

SCF Done: E(RB+HF-LYP) = -1510.33577908 A.U. after 1 cycles
Convg = 0.4582D-08 -V/T = 2.0045
S**2 = 0.0000

Harmonic frequencies (cm**-1), IR intensities (KM/Mole)

	1	2	3
	A	A	A
Frequencies --	-275.9464	14.7391	20.1507
Red. masses --	7.2782	5.0814	4.9772
Frc consts --	0.3265	0.0007	0.0012
IR Inten --	400.8325	2.1907	0.1895

Zero-point correction= 0.510103 (a.u.)
Thermal correction to Energy= 0.557274
Thermal correction to Enthalpy= 0.558218
Thermal correction to Gibbs Free Energy= 0.425738
Sum of electronic and zero-point Energies= -1509.825677
Sum of electronic and thermal Energies= -1509.778505
Sum of electronic and thermal Enthalpies= -1509.777561
Sum of electronic and thermal Free Energies= -1509.910041

E (Thermal) CV S

	KCal/Mol	Cal/Mol-Kelvin	Cal/Mol-Kelvin
Total	349.694	159.903	278.827

==TS3(II), proton removal at the para position by B3LYP/6-31G(d)==
Stoichiometry C6H38NO16(1+) bamb21.log
Standard orientation:

Center Number	Atomic Number	Atomic Type	Coordinates (Angstroms) X	Y	Z
1	6	0	0.098006	-1.870473	-1.375090
2	7	0	1.242517	-2.560180	-1.452892
3	6	0	-0.023907	-0.576962	-1.971795
4	6	0	-1.035607	-2.435817	-0.705318
5	6	0	-1.169566	0.148200	-1.808755
6	6	0	-2.182651	-1.709458	-0.560848
7	6	0	-2.227829	-0.309194	-0.932782
8	1	0	2.094916	-2.117329	-1.803808
9	1	0	0.809350	-0.158888	-2.526689
10	1	0	-0.969452	-3.455354	-0.337595
11	1	0	-1.250946	1.142730	-2.235914
12	1	0	-3.060098	-2.140264	-0.086101
13	1	0	-1.672097	0.137900	0.174840
14	8	0	-0.966403	0.506548	1.299224
15	1	0	-0.384420	1.327776	1.054737
16	1	0	1.392188	-3.316539	-0.786861
17	8	0	-3.498479	0.278993	-1.125491
18	1	0	-1.604110	0.798553	2.020642
19	1	0	-3.781145	0.789449	-0.325638
20	8	0	3.912014	-1.481700	-1.865639
21	1	0	4.415145	-1.532968	-2.692703
22	1	0	3.928512	-0.522530	-1.588683
23	8	0	1.989065	-3.523232	1.119945
24	1	0	1.960307	-4.205749	1.807870
25	1	0	2.956412	-3.361205	0.939423
26	8	0	-2.779664	1.332699	3.056350
27	1	0	-3.573081	1.409768	2.474703
28	1	0	-3.025220	0.727541	3.771630
29	8	0	4.526145	-2.933210	0.411190
30	1	0	5.110665	-3.678218	0.206509
31	1	0	4.429312	-2.433915	-0.434647
32	8	0	0.602221	2.457446	0.713097
33	1	0	1.445719	2.136193	1.109259
34	1	0	0.797435	2.473207	-0.261739
35	1	0	-4.847156	-0.887987	-1.264265
36	8	0	-5.574685	-1.514829	-1.034172
37	1	0	-6.012712	-1.732813	-1.870945
38	8	0	1.406244	2.359250	-1.900563
39	1	0	2.279979	1.949994	-1.722386
40	1	0	1.614270	3.269370	-2.208544
41	1	0	-6.513718	-0.580919	0.063756
42	8	0	-6.826864	0.076170	0.741977
43	1	0	-7.183018	-0.450073	1.473275
44	1	0	-5.504950	0.960962	1.046254
45	8	0	-4.639731	1.484967	1.058615
46	1	0	-4.896249	2.403391	0.876954
47	8	0	2.131151	4.978602	-2.583238

48	1	0	1.886212	5.312728	-3.459895
49	1	0	1.749537	5.616476	-1.960436
50	8	0	2.859696	1.039781	1.449251
51	1	0	3.478576	1.245265	2.190043
52	1	0	2.357171	0.209610	1.715621
53	8	0	4.630088	1.609543	3.495047
54	1	0	4.297593	2.259843	4.133137
55	1	0	4.860959	0.834056	4.029740
56	8	0	3.778486	1.057362	-1.006227
57	1	0	3.494684	1.015165	-0.034944
58	1	0	4.585746	1.595175	-1.019031
59	8	0	1.323259	-0.984324	2.187261
60	1	0	0.439861	-0.673229	1.899349
61	1	0	1.465176	-1.876216	1.797855

SCF Done: E(RB+HF-LYP) = -1509.64405337 A.U. after 1 cycles

Harmonic frequencies (cm**-1), IR intensities (KM/Mole)

	1	2	3
	A	A	A
Frequencies --	-862.1060	13.3636	15.6807
Red. masses --	1.2969	4.6716	5.1169
Frc consts --	0.5679	0.0005	0.0007
IR Inten --	3921.7959	2.0337	2.5610

Zero-point correction= 0.509753 (a.u.)
Thermal correction to Energy= 0.554931
Thermal correction to Enthalpy= 0.555875
Thermal correction to Gibbs Free Energy= 0.426059
Sum of electronic and zero-point Energies= -1509.134300
Sum of electronic and thermal Energies= -1509.089122
Sum of electronic and thermal Enthalpies= -1509.088178
Sum of electronic and thermal Free Energies= -1509.217994

	E (Thermal)	CV	S
	KCal/Mol	Cal/Mol-Kelvin	Cal/Mol-Kelvin
Total	348.225	156.059	273.222

==TS4(II) proton attach to N(4) by B3LYP/6-31G(d)==

Stoichiometry C6H38NO16(1+) bamb21x1.log
Standard orientation:

Center Number	Atomic Number	Atomic Type	Coordinates (Angstroms) X	Y	Z
1	6	0	1.320549	0.741080	-0.990777
2	7	0	-0.099124	0.657655	-1.049795
3	6	0	1.950970	1.951960	-0.660223
4	6	0	2.118057	-0.401721	-1.165920
5	6	0	3.333068	2.023494	-0.531444
6	6	0	3.509485	-0.337552	-1.011537
7	6	0	4.125328	0.878945	-0.700853
8	1	0	-0.536179	1.402073	-1.588608
9	1	0	1.348142	2.842368	-0.500168
10	1	0	1.651595	-1.333608	-1.477912
11	1	0	3.816236	2.965987	-0.291942

12	1	0	4.118980	-1.224830	-1.157080
13	1	0	-0.672724	1.030341	0.628942
14	8	0	-0.856919	1.206657	1.594191
15	1	0	-2.459037	0.926563	1.869398
16	1	0	-0.416362	-0.244657	-1.421014
17	8	0	5.478338	1.002262	-0.558516
18	1	0	-0.270759	0.568623	2.038433
19	1	0	5.937211	0.136359	-0.785304
20	8	0	-2.894982	1.316695	-2.127781
21	1	0	-3.497989	1.671324	-1.434646
22	1	0	-3.191717	1.710096	-2.963064
23	8	0	-0.799699	-2.129807	-1.816924
24	1	0	-0.803849	-2.617956	-0.969696
25	1	0	-1.668002	-2.291348	-2.220695
26	8	0	-3.415079	0.609062	1.875970
27	1	0	-4.157352	1.249340	0.767459
28	1	0	-3.327730	-0.339460	1.630864
29	8	0	-3.679063	-1.288185	-1.790795
30	1	0	-3.315900	-1.535841	-0.904214
31	1	0	-3.257549	-0.416770	-2.017852
32	8	0	-4.728515	1.588579	-0.049621
33	1	0	-5.450356	0.529363	-0.441361
34	1	0	-5.290841	2.343029	0.273634
35	1	0	6.317173	1.388833	1.017206
36	8	0	6.921187	1.384254	1.794709
37	1	0	7.450841	2.190785	1.701893
38	8	0	-3.211851	-1.961531	0.824715
39	1	0	-2.378325	-2.461626	1.016216
40	1	0	-3.979257	-2.497967	1.092187
41	1	0	7.915686	-0.010585	1.483225
42	8	0	8.349613	-0.840546	1.152512
43	1	0	8.333622	-1.452966	1.902775
44	1	0	7.358932	-1.268836	-0.138835
45	8	0	6.729973	-1.301916	-0.916370
46	1	0	7.301891	-1.329043	-1.698763
47	8	0	1.016259	-1.088082	1.743983
48	1	0	1.475086	-0.753731	0.941533
49	1	0	1.722907	-1.250018	2.389536
50	8	0	-0.731420	-3.018146	0.908967
51	1	0	-0.070682	-2.369418	1.269613
52	1	0	-0.427373	-3.897858	1.179578
53	8	0	-6.029354	-2.210575	1.284567
54	1	0	-6.204932	-1.796378	2.146324
55	1	0	-6.692887	-2.915740	1.200215
56	8	0	-5.870974	-0.430816	-0.834079
57	1	0	-5.061404	-0.856516	-1.347731
58	1	0	-6.059519	-1.036663	-0.071707
59	8	0	-6.324949	3.534751	0.967498
60	1	0	-5.979043	3.968256	1.763398
61	1	0	-6.649948	4.256414	0.407047

SCF Done: E(RB+HF-LYP) = -1509.67303834   A.U. after   1 cycles

Harmonic frequencies (cm**-1), IR intensities (KM/Mole)

	1	2	3
	A	A	A
Frequencies --	-90.0931	12.3334	13.9417

Red. masses -- 2.9166 5.5349 5.4682
Frc consts -- 0.0139 0.0005 0.0006
IR Inten -- 118.2761 1.1431 0.4039

Zero-point correction= 0.512522 (a.u.)
Thermal correction to Energy= 0.557026
Thermal correction to Enthalpy= 0.557970
Thermal correction to Gibbs Free Energy= 0.430170
Sum of electronic and zero-point Energies= -1509.160516
Sum of electronic and thermal Energies= -1509.116012
Sum of electronic and thermal Enthalpies= -1509.115068
Sum of electronic and thermal Free Energies= -1509.242868

	E (Thermal)	CV	S
	KCal/Mol	Cal/Mol-Kelvin	Cal/Mol-Kelvin
Total	349.539	154.357	268.978

==TS2(II,3H2O), water-trimer participating TS in the OH[1,5]shift
by B3LYP/6-31G(d) == bamb29aw.log

Stoichiometry C6H38NO16(1+)
Standard orientation:

Center Number	Atomic Number	Atomic Type	Coordinates (Angstroms) X	Y	Z
1	6	0	-1.815121	-0.538789	-1.114528
2	7	0	-2.532604	0.088660	-0.199906
3	6	0	-1.232367	0.212373	-2.201178
4	6	0	-1.575629	-1.960031	-1.023621
5	6	0	-0.385416	-0.398040	-3.069984
6	6	0	-0.768488	-2.574550	-1.924389
7	6	0	-0.022560	-1.794089	-2.895731
8	1	0	-2.867055	1.079636	-0.397368
9	1	0	-1.503953	1.260688	-2.292472
10	1	0	-2.092635	-2.523096	-0.253118
11	1	0	0.040442	0.147767	-3.906670
12	1	0	-0.617485	-3.648538	-1.897204
13	1	0	0.348688	-2.319702	-3.768905
14	8	0	-1.396946	1.057741	1.263325
15	1	0	0.159731	1.256016	1.084242
16	1	0	-2.964625	-0.480621	0.568368
17	1	0	1.323216	-0.423785	0.566191
18	8	0	1.177293	1.162961	1.164975
19	1	0	1.297607	0.845650	2.093625
20	8	0	1.406402	-1.417365	0.457427
21	1	0	1.559108	-1.720514	-1.146095
22	1	0	2.245692	-1.618105	0.935816
23	1	0	-1.351613	0.329120	1.914743
24	8	0	1.698997	-1.812255	-2.146832
25	1	0	2.200361	-0.985106	-2.394930
26	8	0	-3.161090	2.581474	-0.904334
27	1	0	-2.919468	3.106715	-0.094222
28	1	0	-4.100577	2.790440	-1.103802
29	8	0	-2.385904	3.509114	1.510197
30	1	0	-2.004924	2.591839	1.615854
31	1	0	-3.109749	3.556007	2.152648

32	8	0	-3.245625	-1.541644	1.885613
33	1	0	-3.556307	-2.456218	1.712201
34	1	0	-2.379125	-1.632563	2.336229
35	8	0	-5.828242	3.181029	-1.556260
36	1	0	-6.328181	3.734916	-0.937069
37	1	0	-5.858908	3.665238	-2.395887
38	8	0	-3.918999	-4.168888	1.168216
39	1	0	-3.751732	-4.840959	1.847447
40	1	0	-4.844902	-4.303431	0.911947
41	8	0	1.821162	-0.047251	3.570563
42	1	0	2.614961	-0.485992	3.178494
43	1	0	2.160136	0.588168	4.220039
44	8	0	3.716911	-1.190106	1.950223
45	1	0	4.433735	-1.799877	2.182611
46	1	0	4.110431	-0.491255	1.348917
47	8	0	-0.538020	-1.427204	2.597734
48	1	0	0.073935	-1.088408	3.279099
49	1	0	0.048695	-1.618808	1.834547
50	8	0	3.122857	0.475781	-2.291528
51	1	0	4.100620	0.542797	-2.498008
52	1	0	2.731767	1.298730	-2.623848
53	8	0	5.723512	0.925992	-2.202251
54	1	0	5.642480	0.929208	-1.222237
55	1	0	6.430410	0.297723	-2.413248
56	8	0	4.432185	0.746459	0.242784
57	1	0	3.724009	0.664527	-0.432678
58	1	0	4.182399	1.568991	0.755674
59	8	0	3.320181	2.693688	1.679674
60	1	0	2.410097	2.405163	1.419272
61	1	0	3.381047	3.639575	1.479487

SCF Done: E(RB+HF-LYP) = -1509.56760624 A.U. after 3 cycles
Convg = 0.1972D-08 -V/T = 2.0088
S**2 = 0.0000

Harmonic frequencies (cm**-1), IR intensities (KM/Mole)

	1	2	3
	A	A	A
Frequencies --	-268.4893	12.1199	18.5406
Red. masses --	7.3127	5.3073	5.2871
Frc consts --	0.3106	0.0005	0.0011
IR Inten --	455.0921	1.0672	0.7265
Atom AN	X Y Z	X Y Z	X Y Z

Zero-point correction= 0.514579 (a.u.)
Thermal correction to Energy= 0.558936
Thermal correction to Enthalpy= 0.559880
Thermal correction to Gibbs Free Energy= 0.433719
Sum of electronic and zero-point Energies= -1509.053027
Sum of electronic and thermal Energies= -1509.008670
Sum of electronic and thermal Enthalpies= -1509.007726
Sum of electronic and thermal Free Energies= -1509.133888

	E (Thermal)	CV	S
	KCal/Mol	Cal/Mol-Kelvin	Cal/Mol-Kelvin
Total	350.738	153.952	265.530

==TS2(II,3H2O) by B3LYP/6-311+G(d,p)== bamb29aw.high.log

Stoichiometry C6H38NO16(1+)

Standard orientation:

Center Number	Atomic Number	Atomic Type	Coordinates (Angstroms) X	Y	Z
1	6	0	-1.688371	-0.671935	1.275189
2	7	0	-2.558135	-0.340076	0.351293
3	6	0	-1.107779	-1.995821	1.275693
4	6	0	-1.289852	0.286487	2.281377
5	6	0	-0.139975	-2.303996	2.174427
6	6	0	-0.363212	-0.054030	3.208479
7	6	0	0.305590	-1.324955	3.129044
8	1	0	-2.969509	-1.089235	-0.264999
9	1	0	-1.472121	-2.709151	0.544958
10	1	0	-1.780689	1.252221	2.288744
11	1	0	0.310579	-3.288278	2.187258
12	1	0	-0.080977	0.635981	3.993204
13	1	0	0.859091	-1.666562	3.991476
14	8	0	-1.716478	0.318072	-1.590904
15	1	0	-0.186819	0.548050	-1.564903
16	1	0	-2.933049	0.630245	0.347699
17	1	0	1.347067	1.147471	0.059954
18	8	0	0.823992	0.700508	-1.547623
19	1	0	0.981429	1.561473	-1.975280
20	8	0	1.665083	1.506995	0.926490
21	1	0	1.979416	0.145363	1.938758
22	1	0	2.462973	2.016658	0.694346
23	1	0	-2.180432	1.167403	-1.602597
24	8	0	2.146002	-0.691245	2.447389
25	1	0	2.549717	-1.315665	1.792010
26	8	0	-3.371136	-2.493655	-1.027653
27	1	0	-3.080513	-2.405229	-1.962750
28	1	0	-4.225686	-2.959801	-1.013448
29	8	0	-2.179459	-1.678768	-3.305878
30	1	0	-2.019466	-0.830156	-2.818311
31	1	0	-2.501584	-1.452290	-4.182624
32	8	0	-2.962490	2.375414	0.306137
33	1	0	-3.686289	3.015754	0.427815
34	1	0	-2.116847	2.872096	0.267582
35	8	0	-5.821884	-3.892864	-0.903915
36	1	0	-6.691363	-3.586274	-1.181352
37	1	0	-5.866656	-4.854756	-0.899831
38	8	0	-5.114841	4.162057	0.649043
39	1	0	-5.265753	4.719538	1.419477
40	1	0	-5.617099	4.558670	-0.070350
41	8	0	1.571833	3.410610	-1.973181
42	1	0	2.453157	3.326576	-1.555303
43	1	0	1.684411	3.977615	-2.743188
44	8	0	3.849458	2.714167	-0.483072
45	1	0	4.558485	3.305446	-0.208240
46	1	0	4.270417	1.853540	-0.727489
47	8	0	-0.415722	3.489521	0.289587
48	1	0	0.051384	3.711603	-0.530871
49	1	0	0.216857	2.920511	0.758568

50	8	0	3.300092	-2.097723	0.461340
51	1	0	4.187449	-2.493176	0.378558
52	1	0	2.926355	-2.063489	-0.439672
53	8	0	5.966476	-2.506009	-0.203834
54	1	0	6.224558	-1.692239	-0.655559
55	1	0	6.765042	-3.026950	-0.075541
56	8	0	4.681373	0.158359	-0.959016
57	1	0	4.370510	-0.309352	-0.167365
58	1	0	4.129091	-0.245155	-1.656302
59	8	0	2.549033	-1.258770	-2.130256
60	1	0	1.828797	-0.584559	-2.027511
61	1	0	2.355954	-1.761226	-2.928472

SCF Done: E(RB+HF-LYP) = -1510.34075409 A.U. after 1 cycles

Harmonic frequencies (cm**-1), IR intensities (KM/Mole)

	1	2	3
	A	A	A
Frequencies --	-191.2037	18.2667	21.1863
Red. masses --	7.0403	5.0500	5.0525
Frc consts --	0.1516	0.0010	0.0013
IR Inten --	320.3965	1.8815	0.3667

Zero-point correction= 0.509478 (a.u.)
Thermal correction to Energy= 0.557069
Thermal correction to Enthalpy= 0.558013
Thermal correction to Gibbs Free Energy= 0.423910
Sum of electronic and zero-point Energies= -1509.831276
Sum of electronic and thermal Energies= -1509.783685
Sum of electronic and thermal Enthalpies= -1509.782741
Sum of electronic and thermal Free Energies= -1509.916844

	E (Thermal)	CV	S
	KCal/Mol	Cal/Mol-Kelvin	Cal/Mol-Kelvin
Total	349.566	160.574	282.244

==TS2(II, [1,3]shift). OH shift TS to the ortho carbon
by B3LYP/6-31G(d)==

Stoichiometry C6H38NO16(1+) bamb07a.log
Standard orientation:

Center Number	Atomic Number	Atomic Type	Coordinates (Angstroms) X	Y	Z
1	6	0	1.696752	-2.201979	-0.148568
2	7	0	2.616094	-1.420447	0.392452
3	6	0	0.799457	-2.974732	0.665439
4	6	0	1.498837	-2.125058	-1.587866
5	6	0	-0.130194	-3.761371	0.052014
6	6	0	0.575764	-3.024464	-2.181131
7	6	0	-0.220271	-3.804667	-1.378738
8	1	0	2.804325	-1.464343	1.432195
9	1	0	0.882411	-2.915900	1.746412
10	1	0	2.287270	-1.672804	-2.177177
11	1	0	-0.805293	-4.371663	0.642725
12	1	0	0.492085	-3.065675	-3.261524

13	1	0	-0.936183	-4.483411	-1.833711
14	8	0	1.698400	0.463349	0.523476
15	1	0	2.370427	1.136223	0.256145
16	1	0	3.342580	-0.988322	-0.229812
17	1	0	0.998906	0.120652	-0.699736
18	8	0	0.582862	-0.263464	-1.587425
19	1	0	-0.398354	-0.445064	-1.358879
20	8	0	-1.839887	-0.666269	-0.736934
21	1	0	-1.969248	0.081451	-0.117379
22	1	0	-2.595651	-0.570170	-1.381486
23	8	0	3.077060	-1.606253	3.051061
24	1	0	2.190825	-1.520698	3.503325
25	1	0	3.457219	-2.435855	3.376849
26	8	0	4.311982	-0.345232	-1.451330
27	1	0	5.271658	-0.465898	-1.394280
28	1	0	4.141389	0.634818	-1.362019
29	1	0	0.669440	0.725399	1.797195
30	8	0	-0.051281	0.770162	2.496493
31	1	0	-0.885901	0.677042	1.993368
32	1	0	0.244839	-0.626935	3.451118
33	8	0	0.554705	-1.462442	3.895608
34	1	0	0.354474	-1.345395	4.836688
35	8	0	3.396105	2.091304	-0.986674
36	1	0	2.710589	2.227906	-1.694347
37	1	0	3.825011	2.961962	-0.818581
38	8	0	1.414128	2.073220	-2.837346
39	1	0	1.708046	1.922497	-3.748386
40	1	0	0.991844	1.226417	-2.559729
41	8	0	4.513834	4.582985	-0.480081
42	1	0	5.172073	4.674352	0.225494
43	1	0	4.856220	5.116093	-1.213592
44	8	0	-4.013974	-0.157144	-2.254028
45	1	0	-4.753725	-0.505880	-1.720647
46	1	0	-4.057912	0.812888	-2.104871
47	8	0	-3.979616	2.427528	-1.182783
48	1	0	-3.216236	2.272886	-0.575328
49	1	0	-3.816503	3.274303	-1.625268
50	8	0	-6.012899	-0.920039	-0.264407
51	1	0	-6.957559	-0.840803	-0.474421
52	1	0	-5.912087	-1.807340	0.115731
53	8	0	-2.397604	1.580214	0.923237
54	1	0	-3.322433	1.427645	1.267264
55	1	0	-2.022988	2.349406	1.435131
56	8	0	-5.062994	1.383799	1.207000
57	1	0	-5.109743	1.982655	0.436510
58	1	0	-5.397159	0.535492	0.853545
59	8	0	-1.094698	3.313882	2.532639
60	1	0	-0.511350	2.558135	2.774210
61	1	0	-0.507037	3.979876	2.145154

SCF Done: E(RB+HF-LYP) = -1509.56573844 A.U. after 1 cycles
Convg = 0.3265D-08 -V/T = 2.0088
S**2 = 0.0000

Harmonic frequencies (cm**-1), IR intensities (KM/Mole)
1 2 3
A A A

Frequencies --	-394.9011	11.4868	13.7008
Red. masses --	6.4318	5.1136	4.9548
Frc consts --	0.5910	0.0004	0.0005
IR Inten --	71.2319	1.0722	0.0747

Zero-point correction= 0.512076 (a.u.)
Thermal correction to Energy= 0.557058
Thermal correction to Enthalpy= 0.558002
Thermal correction to Gibbs Free Energy= 0.429086
Sum of electronic and zero-point Energies= -1509.053662
Sum of electronic and thermal Energies= -1509.008680
Sum of electronic and thermal Enthalpies= -1509.007736
Sum of electronic and thermal Free Energies= -1509.136653

	E (Thermal)	CV	S
	KCal/Mol	Cal/Mol-Kelvin	Cal/Mol-Kelvin
Total	349.559	154.843	271.328

==TS2(II,[1,3]shift) by B3LYP/6-311+G(d,p)==

Stoichiometry C6H38NO16(1+) bamb07a.high.log
Standard orientation:

Center Number	Atomic Number	Atomic Type	Coordinates (Angstroms) X	Y	Z
1	6	0	1.341052	-2.226258	-0.407272
2	7	0	2.359822	-1.887644	0.351547
3	6	0	0.191267	-2.895046	0.141327
4	6	0	1.341327	-1.815653	-1.802761
5	6	0	-0.814820	-3.264253	-0.696556
6	6	0	0.309634	-2.276249	-2.640663
7	6	0	-0.745192	-2.969919	-2.095841
8	1	0	2.345916	-2.142098	1.365793
9	1	0	0.182860	-3.127712	1.199017
10	1	0	2.255794	-1.409151	-2.212393
11	1	0	-1.676294	-3.797262	-0.315071
12	1	0	0.344355	-2.055925	-3.699312
13	1	0	-1.551012	-3.310001	-2.735835
14	8	0	2.055513	0.245759	0.801342
15	1	0	2.905794	0.715541	0.717143
16	1	0	3.254451	-1.569069	-0.085628
17	1	0	1.373145	0.329319	-0.405701
18	8	0	0.830796	0.242066	-1.319600
19	1	0	-0.121257	0.255397	-1.044189
20	8	0	-1.617591	0.280040	-0.108747
21	1	0	-2.057680	1.163896	-0.076043
22	1	0	-2.335239	-0.310922	-0.425500
23	8	0	1.985687	-2.712964	2.930855
24	1	0	1.387683	-2.167579	3.499556
25	1	0	2.537505	-3.241539	3.514654
26	8	0	4.544076	-1.019737	-0.997775
27	1	0	5.414628	-1.402631	-1.140238
28	1	0	4.653579	-0.041393	-0.871961
29	1	0	0.744048	0.681104	1.864535
30	8	0	-0.146274	0.777019	2.284201
31	1	0	-0.759744	0.457511	1.592629

32	1	0	-0.019219	-0.393317	3.601142
33	8	0	0.168089	-1.169958	4.177488
34	1	0	0.106189	-0.871422	5.089337
35	8	0	4.347406	1.576478	-0.505869
36	1	0	3.791608	1.968197	-1.212546
37	1	0	5.040622	2.222293	-0.276837
38	8	0	2.509323	2.173688	-2.479218
39	1	0	2.152442	3.007464	-2.799289
40	1	0	1.747949	1.614692	-2.234383
41	8	0	6.369658	3.405256	0.173115
42	1	0	6.363317	4.002049	0.928516
43	1	0	7.125620	3.659527	-0.366122
44	8	0	-3.864150	-0.869041	-1.240596
45	1	0	-4.614495	-1.012127	-0.643505
46	1	0	-4.081790	-0.055941	-1.731719
47	8	0	-4.302606	1.838013	-2.178079
48	1	0	-3.662615	2.356957	-1.653801
49	1	0	-4.358185	2.250230	-3.046053
50	8	0	-6.120471	-0.940528	0.714900
51	1	0	-7.000595	-1.226650	0.441791
52	1	0	-5.977548	-1.339661	1.581377
53	8	0	-2.932033	2.665197	0.083927
54	1	0	-3.800319	2.514685	0.511181
55	1	0	-2.365407	3.159127	0.712357
56	8	0	-5.585442	1.980785	0.461362
57	1	0	-5.656714	2.020621	-0.503317
58	1	0	-5.836825	1.073764	0.689845
59	8	0	-1.048590	3.437752	1.930739
60	1	0	-0.657166	2.591321	2.225675
61	1	0	-1.000883	4.049886	2.670322

SCF Done: E(RB+HF-LYP) = -1510.33948623 A.U. after 2 cycles
Convg = 0.1995D-08 -V/T = 2.0045
S**2 = 0.0000

Harmonic frequencies (cm**-1), IR intensities (KM/Mole)

	1	2	3
	A	A	A
Frequencies --	-399.3957	11.5050	14.1378
Red. masses --	4.9558	5.3189	5.1515
Frc consts --	0.4658	0.0004	0.0006
IR Inten --	23.1384	0.5404	1.2129

Zero-point correction= 0.507905 (a.u.)
Thermal correction to Energy= 0.555416
Thermal correction to Enthalpy= 0.556360
Thermal correction to Gibbs Free Energy= 0.421733
Sum of electronic and zero-point Energies= -1509.831581
Sum of electronic and thermal Energies= -1509.784070
Sum of electronic and thermal Enthalpies= -1509.783126
Sum of electronic and thermal Free Energies= -1509.917754

	E (Thermal)	CV	S
	KCal/Mol	Cal/Mol-Kelvin	Cal/Mol-Kelvin
Total	348.529	160.560	283.347

[2] TS geometries in Figure 3

==TS1(III) proton attach to N(2) by B3LYP/6-31G(d)==

Stoichiometry C6H39NO16(2+) bamb03c.log
Standard orientation:

Center Number	Atomic Number	Atomic Type	Coordinates (Angstroms) X	Y	Z
1	6	0	2.494410	-1.976423	0.186106
2	7	0	1.902531	-1.010062	-0.720193
3	6	0	3.786950	-1.756024	0.662553
4	6	0	1.772198	-3.113194	0.561753
5	6	0	4.366693	-2.695280	1.518677
6	6	0	2.356881	-4.042533	1.418438
7	6	0	3.654532	-3.833928	1.896782
8	1	0	2.747889	0.011132	-1.200412
9	1	0	4.348494	-0.879680	0.354608
10	1	0	0.762434	-3.259858	0.188914
11	1	0	5.376493	-2.537080	1.884788
12	1	0	1.805260	-4.931045	1.710161
13	1	0	4.110331	-4.563065	2.559834
14	8	0	0.795279	-0.354414	-0.023847
15	1	0	0.292970	0.123978	-0.747748
16	1	0	1.431605	-1.500164	-1.493228
17	8	0	-5.079862	-1.517816	-2.993153
18	1	0	-5.252919	-1.150856	-3.876971
19	1	0	-5.261430	-2.470030	-3.075162
20	8	0	3.328707	0.894018	-1.605124
21	1	0	-0.552453	2.364865	0.851322
22	1	0	3.181053	1.746108	-0.999756
23	8	0	-1.389218	2.450203	0.308749
24	1	0	-1.712768	3.354954	0.443993
25	1	0	-2.471052	1.081241	0.673094
26	8	0	-0.056222	-1.506597	-2.898850
27	1	0	0.141690	-1.945610	-3.741838
28	1	0	-0.012175	-0.546591	-3.075827
29	1	0	-2.798775	-0.306523	-0.015084
30	8	0	-3.002061	0.250014	0.772900
31	1	0	-4.371700	0.489917	0.909713
32	1	0	4.308785	0.700366	-1.608413
33	8	0	5.953042	0.355413	-1.382239
34	1	0	6.450660	-0.128046	-2.060830
35	8	0	-0.644121	0.992465	-1.887068
36	1	0	-1.510220	0.524913	-1.902182
37	1	0	-0.820021	1.801323	-1.354921
38	8	0	-2.577643	-0.935732	-1.716384
39	1	0	-3.375822	-1.147997	-2.245224
40	1	0	-1.844436	-1.492830	-2.054360
41	8	0	3.041248	2.882455	-0.090055
42	1	0	2.789691	3.729268	-0.533751
43	1	0	2.328086	2.686620	0.564442
44	8	0	2.261751	5.265167	-1.234958
45	1	0	2.645427	6.038374	-0.791520
46	1	0	2.438274	5.411434	-2.177597
47	8	0	0.973551	1.950444	1.472836

48	1	0	1.049315	1.018245	1.161440
49	1	0	1.037699	1.939367	2.463235
50	8	0	1.038506	2.007895	4.199115
51	1	0	1.323853	1.242786	4.722103
52	1	0	1.460556	2.772126	4.621755
53	8	0	-5.432219	0.675295	1.002848
54	1	0	-5.779455	0.398360	1.910451
55	1	0	-5.942190	0.154459	0.288296
56	8	0	-6.521809	-0.037976	3.277351
57	1	0	-6.947443	0.635646	3.830425
58	1	0	-6.176131	-0.707770	3.887160
59	8	0	-6.672386	-0.722703	-0.792866
60	1	0	-7.571720	-0.446109	-1.027180
61	1	0	-6.200716	-0.908351	-1.633037
62	1	0	6.530823	1.085814	-1.109238

SCF Done: E(RB+HF-LYP) = -1509.92985118 A.U. after 1 cycles

Harmonic frequencies (cm**-1), IR intensities (KM/Mole)

	1	2	3
	A	A	A
Frequencies --	-216.4096	8.7660	11.0892
Red. masses --	2.5923	5.2138	5.0406
Frc consts --	0.0715	0.0002	0.0004
IR Inten --	664.6407	1.3563	0.0247

Zero-point correction= 0.519699 (a.u.)
Thermal correction to Energy= 0.566992
Thermal correction to Enthalpy= 0.567936
Thermal correction to Gibbs Free Energy= 0.428589
Sum of electronic and zero-point Energies= -1509.410152
Sum of electronic and thermal Energies= -1509.362859
Sum of electronic and thermal Enthalpies= -1509.361915
Sum of electronic and thermal Free Energies= -1509.501262

	E (Thermal)	CV	S
	KCal/Mol	Cal/Mol-Kelvin	Cal/Mol-Kelvin
Total	355.793	158.109	293.281

==TS2(III), OH shift to the para carbon, by B3LYP/6-31G(d)==

Stoichiometry C6H39NO16(2+) bamb23k3.log

Standard orientation:

Center Number	Atomic Number	Atomic Type	Coordinates (Angstroms) X	Y	Z
1	6	0	0.903505	-2.070771	0.595286
2	7	0	2.215337	-2.355000	0.703207
3	6	0	0.344107	-1.014705	1.383853
4	6	0	0.092332	-2.824358	-0.312035
5	6	0	-1.002974	-0.750541	1.290607
6	6	0	-1.249226	-2.540991	-0.387777
7	6	0	-1.802145	-1.499605	0.395160
8	1	0	2.739885	-1.974620	1.545582
9	1	0	0.995466	-0.456483	2.049386
10	1	0	0.551669	-3.613193	-0.900210

11	1	0	-1.457282	0.036524	1.888873
12	1	0	-1.893008	-3.107948	-1.051656
13	1	0	-2.871281	-1.305062	0.330295
14	8	0	3.317217	-1.453871	-0.587629
15	1	0	2.738014	-0.461079	-0.764940
16	1	0	2.556270	-3.244518	0.274018
17	1	0	1.392663	0.800252	-1.464128
18	8	0	2.172096	0.672719	-0.798555
19	1	0	2.820535	1.373583	-1.059198
20	8	0	0.334096	1.253242	-2.484609
21	1	0	-0.587492	1.030701	-2.165519
22	1	0	0.394607	2.224876	-2.522585
23	8	0	3.463590	-1.166594	2.752992
24	1	0	4.278238	-0.781090	2.346577
25	1	0	3.726145	-1.588570	3.585791
26	8	0	5.303586	-0.360226	0.964049
27	1	0	4.117119	-1.157470	-0.063512
28	1	0	5.380782	0.624250	0.771282
29	8	0	2.935291	-4.557229	-0.753329
30	1	0	3.160264	-5.475819	-0.535835
31	1	0	3.510319	-4.306100	-1.493477
32	1	0	6.210847	-0.702673	0.912939
33	8	0	5.575343	2.192356	0.330995
34	1	0	5.826950	2.867732	0.977888
35	1	0	4.933494	2.608661	-0.282248
36	8	0	-2.046099	0.485308	-1.555031
37	1	0	-2.569150	1.017052	-0.882099
38	1	0	-2.716840	0.251717	-2.226557
39	8	0	-4.541626	-0.032556	-2.914454
40	1	0	-5.002632	0.820535	-2.969452
41	1	0	-4.782467	-0.504002	-3.728349
42	8	0	-3.722905	1.598291	0.182797
43	1	0	-4.317366	0.810936	0.189803
44	1	0	-3.402488	1.683950	1.109356
45	8	0	3.746603	2.752495	-1.687921
46	1	0	4.244177	2.568396	-2.501233
47	1	0	3.021252	3.368221	-1.953231
48	8	0	1.428528	4.039539	-2.533740
49	1	0	1.054981	4.759409	-1.998219
50	1	0	1.425330	4.380278	-3.444262
51	8	0	-2.752623	1.616926	2.814090
52	1	0	-3.438374	1.296730	3.419856
53	1	0	-2.547513	2.530324	3.129567
54	8	0	-4.925658	-0.845022	-0.192596
55	1	0	-5.840850	-1.117958	0.046711
56	1	0	-4.932981	-0.758680	-1.166335
57	8	0	-7.576184	-1.589446	0.151437
58	1	0	-7.834920	-2.494682	0.382046
59	1	0	-8.237979	-1.023638	0.577336
60	8	0	-2.307137	4.204326	3.671622
61	1	0	-3.002550	4.839806	3.441426
62	1	0	-2.097736	4.388419	4.600375

SCF Done: E(RB+HF-LYP) = -1509.87795140 A.U. after 1 cycles

Harmonic frequencies (cm**-1), IR intensities (KM/Mole)

1 2 3

	A	A	A
Frequencies --	-535.8060	13.0799	15.2259
Red. masses --	2.1026	5.0936	5.2152
Frc consts --	0.3556	0.0005	0.0007
IR Inten --	1858.1060	0.2290	0.5410

Zero-point correction= 0.516893 (a.u.)
Thermal correction to Energy= 0.565777
Thermal correction to Enthalpy= 0.566721
Thermal correction to Gibbs Free Energy= 0.426925
Sum of electronic and zero-point Energies= -1509.361059
Sum of electronic and thermal Energies= -1509.312174
Sum of electronic and thermal Enthalpies= -1509.311230
Sum of electronic and thermal Free Energies= -1509.451027

	E (Thermal)	CV	S
	KCal/Mol	Cal/Mol-Kelvin	Cal/Mol-Kelvin
Total	355.030	163.486	294.226

==TS2(III) by B3LYP/6-311+G(d,p)==
Stoichiometry C6H39NO16(2+) bamb23k3.high.log

Standard orientation:

Center Number	Atomic Number	Atomic Type	Coordinates (Angstroms) X	Y	Z
1	6	0	0.793580	-2.168752	0.333102
2	7	0	2.092152	-2.496441	0.434579
3	6	0	0.219323	-1.248924	1.264742
4	6	0	0.005859	-2.747112	-0.710052
5	6	0	-1.117972	-0.948871	1.170276
6	6	0	-1.326240	-2.429210	-0.785785
7	6	0	-1.890288	-1.526689	0.140269
8	1	0	2.605467	-2.271799	1.330120
9	1	0	0.847659	-0.815419	2.034384
10	1	0	0.473426	-3.432556	-1.407404
11	1	0	-1.581243	-0.263698	1.872423
12	1	0	-1.950389	-2.861236	-1.557373
13	1	0	-2.945420	-1.281739	0.054070
14	8	0	3.258587	-1.419433	-0.658756
15	1	0	2.767927	-0.366999	-0.807386
16	1	0	2.430213	-3.323219	-0.102603
17	1	0	1.525354	1.060111	-1.442572
18	8	0	2.338484	0.794771	-0.881982
19	1	0	3.027468	1.465271	-1.076039
20	8	0	0.416488	1.696978	-2.284907
21	1	0	-0.536889	1.514256	-2.076733
22	1	0	0.531495	2.651827	-2.380196
23	8	0	3.426355	-1.729672	2.629381
24	1	0	4.248533	-1.278937	2.345668
25	1	0	3.574862	-2.120929	3.497087
26	8	0	5.314404	-0.583689	1.028935
27	1	0	4.057120	-1.243052	-0.099956
28	1	0	5.348235	0.406607	0.987080
29	8	0	2.916122	-4.627851	-1.111651
30	1	0	2.945885	-5.575043	-0.930385

31	1	0	3.523519	-4.469834	-1.844496
32	1	0	6.225604	-0.885698	0.931713
33	8	0	5.426861	2.089887	0.822247
34	1	0	5.880978	2.711626	1.399834
35	1	0	4.979561	2.600829	0.125829
36	8	0	-2.080076	1.024460	-1.664876
37	1	0	-2.540585	1.311353	-0.839124
38	1	0	-2.784648	0.926997	-2.324086
39	8	0	-4.625522	0.662081	-3.148741
40	1	0	-5.125705	1.486744	-3.194417
41	1	0	-4.742438	0.245180	-4.011179
42	8	0	-3.625159	1.521166	0.497722
43	1	0	-4.292004	0.826569	0.338205
44	1	0	-3.415177	1.499132	1.449043
45	8	0	4.031486	2.906975	-1.517988
46	1	0	4.657065	2.854753	-2.251020
47	1	0	3.356571	3.564646	-1.784258
48	8	0	1.802193	4.453762	-2.370323
49	1	0	1.480579	5.168245	-1.805336
50	1	0	1.829768	4.829183	-3.260224
51	8	0	-2.854677	1.166065	3.214318
52	1	0	-3.514813	0.738639	3.771116
53	1	0	-2.611423	1.983280	3.695281
54	8	0	-5.024233	-0.660394	-0.539714
55	1	0	-5.903729	-1.023052	-0.320477
56	1	0	-5.070775	-0.368495	-1.463544
57	8	0	-7.581814	-1.651055	0.062838
58	1	0	-7.906399	-2.554287	-0.017528
59	1	0	-8.316735	-1.133096	0.408454
60	8	0	-2.277093	3.530518	4.586603
61	1	0	-2.822817	4.314830	4.461226
62	1	0	-1.905973	3.604893	5.472585

SCF Done: E(RB+HF-LYP) = -1510.65249649 A.U. after 1 cycles

Harmonic frequencies (cm**-1), IR intensities (KM/Mole)

	1	2	3
	A	A	A
Frequencies --	-574.0128	12.1427	13.8697
Red. masses --	1.7653	5.2088	5.1192
Frc consts --	0.3427	0.0005	0.0006
IR Inten --	2526.1712	0.3159	0.2612

Zero-point correction= 0.514772 (a.u.)
Thermal correction to Energy= 0.565397
Thermal correction to Enthalpy= 0.566341
Thermal correction to Gibbs Free Energy= 0.421380
Sum of electronic and zero-point Energies= -1510.137725
Sum of electronic and thermal Energies= -1510.087099
Sum of electronic and thermal Enthalpies= -1510.086155
Sum of electronic and thermal Free Energies= -1510.231116

	E (Thermal)	CV	S
	KCal/Mol	Cal/Mol-Kelvin	Cal/Mol-Kelvin
Total	354.792	166.957	305.097

==TS3(III) proton removal TS at the para carbon by B3LYP/6-31G(d)==

Stoichiometry   C6H39NO16(2+)   bamb23w.log

Standard orientation:

Center Number	Atomic Number	Atomic Type	Coordinates (Angstroms) X	Y	Z
1	6	0	1.852291	-2.190447	0.417228
2	7	0	3.109752	-2.537746	0.135951
3	6	0	1.018570	-1.587780	-0.581858
4	6	0	1.300606	-2.431074	1.718991
5	6	0	-0.253834	-1.191812	-0.277214
6	6	0	0.033695	-2.015055	2.015786
7	6	0	-0.734949	-1.221121	1.082870
8	1	0	3.523240	-2.278584	-0.760074
9	1	0	1.401278	-1.490958	-1.592172
10	1	0	1.906906	-2.958088	2.449358
11	1	0	-0.899923	-0.783458	-1.050313
12	1	0	-0.395740	-2.206210	2.995157
13	1	0	-0.308238	0.014963	1.445507
14	8	0	-0.118904	1.260470	1.828290
15	1	0	0.661543	1.762175	1.223220
16	1	0	3.679448	-3.020926	0.835130
17	1	0	0.252555	1.199057	2.751126
18	8	0	1.687698	2.420613	0.634817
19	1	0	2.081792	1.998133	-0.175029
20	1	0	1.397337	3.341484	0.374040
21	8	0	0.949480	0.890292	4.301201
22	1	0	0.599485	1.306406	5.104762
23	8	0	3.984731	-1.138300	-2.299767
24	1	0	3.967180	-1.505082	-3.197743
25	1	0	4.779134	-0.559061	-2.269087
26	1	0	1.906867	1.042695	4.338010
27	8	0	2.873147	1.444480	-1.606774
28	1	0	3.802836	1.729408	-1.653459
29	8	0	4.320370	-4.026150	2.304445
30	1	0	5.087774	-3.823356	2.861375
31	1	0	4.307956	-4.994036	2.242326
32	1	0	2.930350	0.499582	-1.846147
33	8	0	0.780587	4.878021	0.067232
34	1	0	1.094297	5.311586	-0.762525
35	1	0	1.006910	5.502864	0.772368
36	8	0	-2.145181	-1.199822	1.278138
37	1	0	-3.299879	-1.407261	0.084196
38	1	0	-2.415976	-0.347069	1.694013
39	8	0	-2.815244	1.403403	1.872302
40	1	0	-3.175617	1.750595	2.703814
41	1	0	-1.862537	1.674992	1.839396
42	8	0	-4.043609	-1.245189	-0.574180
43	1	0	-4.323969	-0.197081	-0.463331
44	1	0	-3.683681	-1.413337	-1.525501
45	8	0	5.754067	1.024623	-2.147850
46	1	0	6.107550	1.399326	-2.971361
47	1	0	6.487185	1.081931	-1.513625
48	8	0	1.656852	6.137133	-2.238765
49	1	0	2.145849	6.971073	-2.160514
50	1	0	0.950523	6.325099	-2.876213

51	8	0	-3.044614	-1.642157	-2.904863
52	1	0	-3.390689	-1.051976	-3.592538
53	1	0	-3.204142	-2.565383	-3.237044
54	8	0	-4.558679	1.154712	-0.288534
55	1	0	-5.486189	1.362276	0.002920
56	1	0	-3.966407	1.462698	0.435485
57	8	0	-7.004351	1.792663	0.685200
58	1	0	-7.445922	2.572090	0.312957
59	1	0	-7.705494	1.130681	0.789790
60	8	0	-3.534162	-4.169060	-3.736703
61	1	0	-4.423678	-4.553599	-3.712941
62	1	0	-3.093951	-4.581555	-4.495314

SCF Done: E(RB+HF-LYP) = -1509.93033501 A.U. after 1 cycles
Convg = 0.2661D-08 -V/T = 2.0088
S**2 = 0.0000

Harmonic frequencies (cm**-1), IR intensities (KM/Mole)

	1	2	3
	A	A	A
Frequencies --	-1140.5160	5.1992	7.6611
Red. masses --	1.1656	5.5010	5.1588
Frc consts --	0.8933	0.0001	0.0002
IR Inten --	6475.1396	0.1317	0.2780

Zero-point correction= 0.511659 (a.u.)
Thermal correction to Energy= 0.562008
Thermal correction to Enthalpy= 0.562952
Thermal correction to Gibbs Free Energy= 0.414274
Sum of electronic and zero-point Energies= -1509.418676
Sum of electronic and thermal Energies= -1509.368327
Sum of electronic and thermal Enthalpies= -1509.367383
Sum of electronic and thermal Free Energies= -1509.516061

	E (Thermal)	CV	S
	KCal/Mol	Cal/Mol-Kelvin	Cal/Mol-Kelvin
Total	352.665	164.988	312.918

==TS4(III), proton attach to N(4) by B3LYP/6-31G(d)==

Stoichiometry C6H39NO16(2+) bamb2yy.log

Standard orientation:

Center Number	Atomic Number	Atomic Type	Coordinates (Angstroms) X	Y	Z
1	6	0	-0.652137	0.042377	-0.283150
2	7	0	-2.056067	0.291522	-0.413208
3	6	0	-0.160558	-1.263355	-0.392234
4	6	0	0.239522	1.084429	-0.004050
5	6	0	1.199761	-1.520164	-0.228306
6	6	0	1.599968	0.831763	0.164769
7	6	0	2.078764	-0.472806	0.051692
8	1	0	-2.488832	-0.349680	-1.097882
9	1	0	-0.845465	-2.075126	-0.620064
10	1	0	-0.131297	2.103452	0.074456

11	1	0	1.573597	-2.536945	-0.323304
12	1	0	2.288558	1.642646	0.382355
13	1	0	-2.909339	0.006074	0.837463
14	8	0	-3.621227	-0.228155	1.600489
15	1	0	-3.650871	0.502339	2.258068
16	1	0	-2.258624	1.260334	-0.678729
17	1	0	-4.681186	-0.286672	1.098439
18	8	0	-5.816430	-0.250456	0.571139
19	1	0	-5.898627	0.632962	0.083860
20	1	0	-5.937101	-0.969193	-0.106021
21	8	0	-5.999376	-2.094959	-1.425115
22	1	0	-6.385710	-2.981529	-1.192997
23	8	0	-3.256336	-1.788087	-2.015412
24	1	0	-4.205626	-1.994010	-1.871870
25	1	0	-3.103957	-1.913520	-2.963405
26	1	0	-6.586390	-1.749557	-2.117522
27	8	0	-5.865346	2.092261	-0.616103
28	1	0	-5.951780	2.767506	0.083343
29	8	0	-3.218762	3.029525	-0.823726
30	1	0	-3.538672	3.492952	-0.015427
31	1	0	-3.028534	3.724444	-1.473154
32	1	0	-4.980279	2.300013	-0.986269
33	8	0	-4.179058	1.913766	3.398239
34	1	0	-4.985834	1.614782	3.852367
35	1	0	-3.545753	2.103626	4.111121
36	8	0	3.435461	-0.692142	0.219661
37	1	0	4.849665	0.258736	0.121745
38	1	0	3.648900	-1.649563	0.211616
39	8	0	4.639671	-3.232370	0.384011
40	1	0	4.445047	-3.749123	1.184110
41	1	0	4.630085	-3.876196	-0.344278
42	8	0	5.810957	0.531882	0.138006
43	1	0	6.371994	-0.359662	0.310142
44	1	0	6.078927	0.948111	-0.783057
45	8	0	-7.264792	-4.433276	-0.979463
46	1	0	-7.868748	-4.580312	-0.235877
47	1	0	-6.925725	-5.309058	-1.218521
48	8	0	-4.717404	3.821569	1.362824
49	1	0	-4.928814	4.714981	1.675897
50	1	0	-4.532186	3.284367	2.162838
51	8	0	6.427501	1.537983	-2.100973
52	1	0	7.065432	1.035819	-2.630590
53	1	0	6.761433	2.474268	-2.073153
54	8	0	7.021953	-1.628594	0.434678
55	1	0	7.592468	-1.747332	1.239407
56	1	0	6.334659	-2.325287	0.475310
57	8	0	8.477637	-2.138823	2.660810
58	1	0	9.321343	-2.598605	2.526469
59	1	0	8.663084	-1.461300	3.329563
60	8	0	7.267814	4.101410	-2.021940
61	1	0	8.171361	4.372190	-1.797970
62	1	0	6.995236	4.686676	-2.745338

SCF Done: E(RB+HF-LYP) = -1509.98950453   A.U. after   1 cycles

Harmonic frequencies (cm**-1), IR intensities (KM/Mole)

	1	2	3
	A	A	A
Frequencies --	-203.9868	3.1781	8.0316
Red. masses --	2.5588	5.3640	5.2665
Frc consts --	0.0627	0.0000	0.0002
IR Inten --	1038.9452	0.5267	1.2425

Zero-point correction= 0.516844 (a.u.)
Thermal correction to Energy= 0.565580
Thermal correction to Enthalpy= 0.566525
Thermal correction to Gibbs Free Energy= 0.420974
Sum of electronic and zero-point Energies= -1509.472661
Sum of electronic and thermal Energies= -1509.423924
Sum of electronic and thermal Enthalpies= -1509.422980
Sum of electronic and thermal Free Energies= -1509.568531

	E (Thermal)	CV	S
	KCal/Mol	Cal/Mol-Kelvin	Cal/Mol-Kelvin
Total	354.907	161.827	306.338

==TS(III,[1.3]shift), OH rearrangement to the ortho carbon by B3LYP/6-31G(d)==

Stoichiometry C6H39NO16(2+) bamb23k5.log

Standard orientation:

Center Number	Atomic Number	Atomic Type	Coordinates (Angstroms) X	Y	Z
1	6	0	-0.974403	-0.141830	-1.641828
2	7	0	-2.143943	-0.743317	-1.841947
3	6	0	-0.871862	1.279152	-1.869628
4	6	0	0.185472	-0.915280	-1.267502
5	6	0	0.362036	1.867557	-1.789504
6	6	0	1.418166	-0.296848	-1.226928
7	6	0	1.506966	1.086494	-1.472004
8	1	0	-2.918866	-0.196157	-2.316755
9	1	0	-1.768875	1.833374	-2.128705
10	1	0	0.081907	-1.974807	-1.050792
11	1	0	0.477261	2.930754	-1.972084
12	1	0	2.307756	-0.870060	-0.969695
13	1	0	2.472096	1.587693	-1.401344
14	8	0	-3.422357	-1.006360	-0.216086
15	1	0	-2.934416	-0.311013	0.738600
16	1	0	-2.202061	-1.780091	-1.824951
17	8	0	-2.393823	0.288243	1.556065
18	1	0	-1.492008	-0.239922	1.712821
19	1	0	-2.933386	0.379017	2.375692
20	8	0	-0.277638	-0.978434	1.514657
21	1	0	0.596910	-0.653108	1.912596
22	1	0	-0.272303	-1.960506	1.443726
23	8	0	-3.970949	0.942759	-2.834210
24	1	0	-4.739086	0.967780	-2.211097
25	1	0	-4.326722	0.990595	-3.735237
26	8	0	-5.674192	0.468784	-0.801172
27	1	0	-4.275335	-0.566657	-0.479300

28	1	0	-5.801863	1.027547	0.020844
29	8	0	-2.586508	-3.373447	-1.024416
30	1	0	-3.129537	-4.081317	-1.407460
31	1	0	-3.184796	-2.784322	-0.502879
32	1	0	-6.529067	0.031928	-0.946242
33	8	0	-6.059526	1.691571	1.518644
34	1	0	-6.439762	2.575938	1.625736
35	1	0	-5.436006	1.559241	2.261007
36	8	0	1.928032	-0.010570	2.504903
37	1	0	2.563269	-0.650753	2.883111
38	1	0	2.495403	0.459398	1.849699
39	8	0	4.408424	-1.313642	2.583560
40	1	0	4.647730	-0.418865	2.258627
41	1	0	5.082678	-1.562386	3.234624
42	8	0	4.011012	0.768024	0.863541
43	1	0	4.162405	-0.001954	0.261364
44	1	0	4.238016	1.579329	0.351511
45	8	0	-4.179622	0.965754	3.575880
46	1	0	-4.600551	0.334950	4.184800
47	1	0	-3.818848	1.665778	4.146628
48	8	0	-0.337456	-3.550171	0.610673
49	1	0	0.022043	-4.393636	0.923746
50	1	0	-1.164214	-3.755545	0.126244
51	8	0	4.267498	2.859006	-0.904819
52	1	0	5.076661	2.865767	-1.438719
53	1	0	4.193384	3.776689	-0.542631
54	8	0	4.147342	-1.710232	-0.212496
55	1	0	4.942842	-2.092162	-0.649124
56	1	0	4.235955	-1.959368	0.731060
57	8	0	6.456741	-2.918709	-1.162017
58	1	0	6.444828	-3.824198	-1.506905
59	1	0	7.232288	-2.503877	-1.568490
60	8	0	4.078365	5.400169	0.123905
61	1	0	4.525661	5.584011	0.964429
62	1	0	4.255215	6.176692	-0.429371

SCF Done: E(RB+HF-LYP) = -1509.87384415 A.U. after 1 cycles

Harmonic frequencies (cm**-1), IR intensities (KM/Mole)

	1	2	3
	A	A	A
Frequencies --	-562.1037	13.6473	17.0134
Red. masses --	1.6808	5.1658	5.1371
Frc consts --	0.3129	0.0006	0.0009
IR Inten --	1141.1798	1.6016	0.2564

Zero-point correction= 0.517832 (a.u.)
Thermal correction to Energy= 0.565391
Thermal correction to Enthalpy= 0.566336
Thermal correction to Gibbs Free Energy= 0.431292
Sum of electronic and zero-point Energies= -1509.356013
Sum of electronic and thermal Energies= -1509.308453
Sum of electronic and thermal Enthalpies= -1509.307509
Sum of electronic and thermal Free Energies= -1509.442552

E (Thermal) CV S

	KCal/Mol	Cal/Mol-Kelvin	Cal/Mol-Kelvin
Total	354.788	161.302	284.224

==TS2(III, [1,3]shift) by B3LYP/6-311+G(d,p)==

Stoichiometry C6H39NO16(2+) bamb23k5.high.log

Standard orientation:

Center Number	Atomic Number	Atomic Type	Coordinates (Angstroms) X	Y	Z
1	6	0	-1.071333	-0.356891	-1.642080
2	7	0	-2.224642	-1.003504	-1.743269
3	6	0	-1.012007	1.029320	-2.032555
4	6	0	0.106240	-1.041311	-1.173947
5	6	0	0.194286	1.672737	-1.992156
6	6	0	1.303927	-0.372637	-1.155551
7	6	0	1.349687	0.981893	-1.553825
8	1	0	-3.028211	-0.547138	-2.256305
9	1	0	-1.920437	1.519258	-2.363583
10	1	0	0.036493	-2.071432	-0.838104
11	1	0	0.275124	2.710134	-2.290904
12	1	0	2.208386	-0.873483	-0.822322
13	1	0	2.291917	1.523888	-1.513517
14	8	0	-3.430111	-1.070829	-0.028350
15	1	0	-2.822338	-0.410172	0.818187
16	1	0	-2.265903	-2.028730	-1.599401
17	8	0	-2.161374	0.189160	1.581006
18	1	0	-1.382750	-0.409940	1.968936
19	1	0	-2.664152	0.632008	2.292320
20	8	0	-0.322806	-1.257353	2.385047
21	1	0	0.586823	-0.862977	2.471949
22	1	0	-0.250119	-2.133096	1.964273
23	8	0	-4.207333	0.395727	-2.876679
24	1	0	-4.929959	0.562151	-2.233502
25	1	0	-4.575228	0.438476	-3.766310
26	8	0	-5.718782	0.450603	-0.613418
27	1	0	-4.266212	-0.599980	-0.252754
28	1	0	-5.690251	1.209838	0.021387
29	8	0	-2.690961	-3.596135	-0.730749
30	1	0	-3.152758	-4.373372	-1.068913
31	1	0	-3.326514	-3.076738	-0.206146
32	1	0	-6.607195	0.079014	-0.556369
33	8	0	-5.428261	2.408354	1.201142
34	1	0	-5.962511	3.201364	1.315777
35	1	0	-4.954852	2.251745	2.034948
36	8	0	2.070626	-0.153840	2.629550
37	1	0	2.786103	-0.670504	3.030932
38	1	0	2.540352	0.396316	1.973120
39	8	0	4.795096	-1.094561	2.688045
40	1	0	4.989458	-0.186944	2.406391
41	1	0	5.462652	-1.332314	3.341115
42	8	0	3.940173	0.929064	0.835774
43	1	0	4.157334	0.151654	0.281796
44	1	0	4.070841	1.718158	0.278897
45	8	0	-3.743000	1.635641	3.456891

46	1	0	-4.168785	1.145895	4.173508
47	1	0	-3.256586	2.349712	3.890611
48	8	0	-0.261066	-3.618950	0.794777
49	1	0	0.216733	-4.407129	1.076916
50	1	0	-1.099622	-3.921434	0.408469
51	8	0	4.017259	2.959896	-1.144179
52	1	0	4.794078	2.934638	-1.714184
53	1	0	3.926535	3.899613	-0.880029
54	8	0	4.231040	-1.624561	-0.135568
55	1	0	4.886604	-2.049920	-0.722402
56	1	0	4.474880	-1.872834	0.770804
57	8	0	6.104263	-2.845616	-1.833110
58	1	0	6.073671	-3.761675	-2.129490
59	1	0	7.008425	-2.549194	-1.984093
60	8	0	3.798326	5.622171	-0.372207
61	1	0	4.188668	5.951983	0.444953
62	1	0	3.752284	6.378829	-0.967162

SCF Done: E(RB+HF-LYP) = -1510.64622108   A.U. after   1 cycles

Harmonic frequencies (cm**-1), IR intensities (KM/Mole)

	1	2	3
	A	A	A
Frequencies --	-666.4421	13.3399	15.2345
Red. masses --	1.3068	5.2442	5.0167
Frc consts --	0.3420	0.0005	0.0007
IR Inten --	2169.6657	0.6350	0.5709

Zero-point correction= 0.515370 (a.u.)
Thermal correction to Energy= 0.564870
Thermal correction to Enthalpy= 0.565815
Thermal correction to Gibbs Free Energy= 0.425234
Sum of electronic and zero-point Energies= -1510.130851
Sum of electronic and thermal Energies= -1510.081351
Sum of electronic and thermal Enthalpies= -1510.080407
Sum of electronic and thermal Free Energies= -1510.220987

	E (Thermal)	CV	S
	KCal/Mol	Cal/Mol-Kelvin	Cal/Mol-Kelvin
Total	354.462	165.527	295.876

[3] TS geometries in Figure 5

==TS2(IV) by B3LYP/6-31G(d)==

Stoichiometry   C6H61NO27(2+) bamb239.log

Standard orientation:

Center Number	Atomic Number	Atomic Type	Coordinates (Angstroms) X	Y	Z
1	6	0	1.586088	2.426018	-1.007164
2	7	0	2.918037	2.560525	-1.058720
3	6	0	0.947842	1.370856	-1.739354
4	6	0	0.816014	3.352139	-0.228474

5	6	0	-0.425893	1.292426	-1.742135
6	6	0	-0.551700	3.247854	-0.242185
7	6	0	-1.179463	2.222746	-0.991326
8	1	0	3.443042	2.025158	-1.812837
9	1	0	1.567044	0.674999	-2.296088
10	1	0	1.337726	4.124693	0.328580
11	1	0	-0.932222	0.511745	-2.304827
12	1	0	-1.163717	3.944979	0.319758
13	1	0	-2.264925	2.188674	-1.018709
14	8	0	3.879751	1.664573	0.416337
15	1	0	3.114151	0.902366	0.668959
16	1	0	3.344110	3.442824	-0.655995
17	1	0	1.446973	-0.069319	1.400528
18	8	0	2.217863	-0.090273	0.723246
19	1	0	2.669511	-0.955191	0.919346
20	8	0	0.286907	-0.381249	2.412322
21	1	0	-0.556426	-0.093582	1.981675
22	1	0	0.284898	-1.367965	2.371649
23	8	0	4.088795	1.007941	-2.898195
24	1	0	4.765358	0.540093	-2.329427
25	1	0	4.557822	1.354360	-3.672420
26	8	0	5.587075	0.099508	-0.934337
27	1	0	4.596060	1.121570	-0.048562
28	1	0	5.504981	-0.883653	-0.688054
29	8	0	3.788041	4.652101	0.341671
30	1	0	4.355635	5.435444	0.129346
31	1	0	4.261036	4.159109	1.031003
32	1	0	6.546024	0.330156	-0.873095
33	8	0	5.416083	-2.419728	-0.247860
34	1	0	5.378902	-3.059740	-0.993094
35	1	0	4.607894	-2.585389	0.288620
36	8	0	-1.937249	0.530008	1.088146
37	1	0	-2.382697	-0.032666	0.416266
38	1	0	-2.694980	0.842386	1.641025
39	8	0	-4.380971	1.320596	2.048173
40	1	0	-4.995965	0.579702	1.788078
41	1	0	-4.749621	1.717316	2.870004
42	8	0	-3.692156	-0.508550	-0.850003
43	1	0	-4.001997	0.441968	-0.831454
44	1	0	-3.313582	-0.662314	-1.750242
45	8	0	3.235566	-2.499911	1.456064
46	1	0	3.585635	-2.478975	2.370034
47	1	0	2.321173	-2.896939	1.559433
48	8	0	0.734810	-3.109195	2.118131
49	1	0	0.061654	-3.663750	1.636135
50	1	0	0.845949	-3.521181	3.012182
51	8	0	-2.192849	-1.308700	-2.996791
52	1	0	-2.648315	-1.699192	-3.767697
53	1	0	-2.061022	-2.069728	-2.384499
54	8	0	-4.441404	2.073261	-0.563739
55	1	0	-5.400957	2.088821	-0.773584
56	1	0	-4.420830	2.041810	0.426288
57	8	0	-7.187610	1.504423	-0.706283
58	1	0	-7.877605	2.063208	-0.268094
59	1	0	-7.597297	1.231625	-1.541747
60	8	0	-2.603673	-3.185881	-0.997280
61	1	0	-3.093945	-2.471409	-0.546775

62	1	0	-3.304902	-3.697030	-1.482797
63	8	0	5.461686	6.771345	-0.053258
64	1	0	5.671746	7.148879	-0.921150
65	1	0	5.315246	7.535953	0.525087
66	8	0	8.228468	0.875959	-0.777519
67	1	0	8.861487	0.521652	-1.421111
68	1	0	8.664007	0.766928	0.082012
69	8	0	5.287965	-4.337679	-2.298175
70	1	0	5.995209	-4.321699	-2.961466
71	1	0	5.328771	-5.231583	-1.923969
72	8	0	-5.980689	-0.524415	0.887466
73	1	0	-6.554209	0.075212	0.362984
74	1	0	-5.335615	-0.857612	0.233585
75	8	0	-5.587241	2.323574	4.359340
76	1	0	-5.090840	2.931966	4.928204
77	1	0	-5.863165	1.605249	4.949761
78	8	0	4.000515	-2.824503	4.238084
79	1	0	4.663084	-3.529681	4.324202
80	1	0	4.323280	-2.101838	4.800412
81	8	0	-1.176569	-4.581455	0.924255
82	1	0	-1.687555	-4.109848	0.223479
83	1	0	-0.889239	-5.413422	0.521404
84	8	0	1.452219	-4.087125	4.556687
85	1	0	2.294750	-3.602600	4.662575
86	1	0	0.910318	-3.849740	5.324020
87	8	0	-3.691972	-2.903110	-4.955689
88	1	0	-4.449779	-2.478309	-5.389978
89	1	0	-3.182963	-3.308361	-5.676691
90	8	0	-4.492279	-4.270586	-2.593055
91	1	0	-4.261372	-3.972398	-3.495980
92	1	0	-4.697145	-5.214897	-2.658837
93	8	0	-9.267075	2.952748	0.303161
94	1	0	-9.785652	2.597617	1.041147
95	1	0	-9.220589	3.908220	0.459077

SCF Done: E(RB+HF-LYP) = -2350.64975739 A.U. after 1 cycles

Harmonic frequencies (cm**-1), IR intensities (KM/Mole)

	1	2	3
	A	A	A
Frequencies --	-302.9751	9.5177	12.6460
Red. masses --	4.3482	5.0311	5.1509
Frc consts --	0.2352	0.0003	0.0005
IR Inten --	639.0152	1.1029	0.3881

Zero-point correction= 0.797619 (a.u.)
Thermal correction to Energy= 0.875684
Thermal correction to Enthalpy= 0.876628
Thermal correction to Gibbs Free Energy= 0.664667
Sum of electronic and zero-point Energies= -2349.852139
Sum of electronic and thermal Energies= -2349.774074
Sum of electronic and thermal Enthalpies= -2349.773129
Sum of electronic and thermal Free Energies= -2349.985090

E (Thermal)	CV	S
KCal/Mol	Cal/Mol-Kelvin	Cal/Mol-Kelvin

Total 549.500 257.178 446.110

==TS2(IV) by B3LYP/6-311+G(d,p)==

Stoichiometry C6H61NO27(2+) bamb239.high.log

Standard orientation:

Center Number	Atomic Number	Atomic Type	Coordinates (Angstroms) X	Y	Z
1	6	0	1.144178	2.483729	-0.978468
2	7	0	2.449063	2.790377	-1.060152
3	6	0	0.650832	1.291740	-1.594418
4	6	0	0.263215	3.370403	-0.285710
5	6	0	-0.698647	1.034572	-1.566607
6	6	0	-1.079222	3.091009	-0.269679
7	6	0	-1.564452	1.930711	-0.907246
8	1	0	3.039689	2.295558	-1.783322
9	1	0	1.350894	0.623128	-2.081209
10	1	0	0.674337	4.254615	0.186950
11	1	0	-1.103770	0.144398	-2.035814
12	1	0	-1.775068	3.753520	0.228589
13	1	0	-2.632897	1.750648	-0.904732
14	8	0	3.505097	2.163999	0.432263
15	1	0	2.962185	1.265970	0.772669
16	1	0	2.741396	3.766553	-0.794776
17	1	0	1.484766	-0.061080	1.509155
18	8	0	2.377298	0.112222	1.063468
19	1	0	2.958418	-0.627836	1.351121
20	8	0	0.224540	-0.703165	2.209135
21	1	0	-0.691578	-0.461881	1.947295
22	1	0	0.328278	-1.666793	2.101793
23	8	0	4.008370	1.427756	-2.755787
24	1	0	4.786513	1.176607	-2.201276
25	1	0	4.304218	1.568906	-3.660256
26	8	0	5.651016	1.015366	-0.701762
27	1	0	4.355872	1.808758	0.033068
28	1	0	5.728335	0.060440	-0.412660
29	8	0	3.079134	5.281249	-0.253207
30	1	0	3.137808	6.128718	-0.744813
31	1	0	3.649557	5.359890	0.518698
32	1	0	6.532269	1.426597	-0.592318
33	8	0	5.768641	-1.490044	0.092882
34	1	0	6.280483	-2.206497	-0.320911
35	1	0	5.070429	-1.888885	0.644771
36	8	0	-2.236471	0.107801	1.343432
37	1	0	-2.765531	-0.423923	0.715815
38	1	0	-2.915367	0.534454	1.904218
39	8	0	-4.594681	1.220175	2.356924
40	1	0	-5.240633	0.488113	2.232578
41	1	0	-4.789259	1.649615	3.209345
42	8	0	-4.064768	-0.878293	-0.554938
43	1	0	-4.361675	0.044177	-0.702908
44	1	0	-3.657103	-1.199309	-1.382617
45	8	0	3.672566	-2.183354	1.822217
46	1	0	3.932855	-2.332926	2.745692

47	1	0	2.840784	-2.707175	1.705173
48	8	0	1.210532	-3.346808	1.798590
49	1	0	0.929422	-3.909306	1.044947
50	1	0	1.231411	-3.909211	2.597957
51	8	0	-2.511901	-1.870909	-2.668039
52	1	0	-2.839619	-2.113921	-3.546764
53	1	0	-2.237435	-2.716049	-2.262906
54	8	0	-4.856049	1.791546	-0.384097
55	1	0	-5.815732	1.778433	-0.542190
56	1	0	-4.774109	1.818512	0.591299
57	8	0	-7.678234	1.082056	-0.422630
58	1	0	-8.377141	1.624004	-0.002759
59	1	0	-8.085034	0.716337	-1.215440
60	8	0	-2.055003	-4.490976	-1.575074
61	1	0	-2.758893	-4.674502	-0.942076
62	1	0	-2.294029	-4.980925	-2.394271
63	8	0	3.195012	7.654727	-1.621591
64	1	0	3.905692	7.974688	-2.187767
65	1	0	2.627760	8.413179	-1.444796
66	8	0	8.117859	2.285293	-0.417165
67	1	0	8.824084	2.258367	-1.071934
68	1	0	8.551227	2.415280	0.433403
69	8	0	7.354406	-3.494479	-1.104083
70	1	0	8.195897	-3.804109	-0.753054
71	1	0	7.130783	-4.089266	-1.827293
72	8	0	-6.238121	-0.756441	1.380068
73	1	0	-6.862465	-0.268697	0.817414
74	1	0	-5.611809	-1.159106	0.757256
75	8	0	-5.079104	2.439412	4.863802
76	1	0	-5.737922	3.111988	5.065359
77	1	0	-4.914333	1.972986	5.690109
78	8	0	4.142193	-2.916989	4.668481
79	1	0	4.965388	-3.377366	4.873878
80	1	0	4.069610	-2.211182	5.322801
81	8	0	0.475589	-4.838414	-0.394104
82	1	0	-0.391145	-4.753523	-0.836811
83	1	0	0.764413	-5.746000	-0.527459
84	8	0	1.817794	-4.619030	4.173443
85	1	0	2.566072	-4.122496	4.539893
86	1	0	1.347569	-5.015356	4.912948
87	8	0	-3.525713	-3.010825	-5.306685
88	1	0	-4.464335	-2.872612	-5.484739
89	1	0	-3.068207	-2.743985	-6.113560
90	8	0	-2.804360	-5.458865	-3.988631
91	1	0	-3.085875	-4.756893	-4.598141
92	1	0	-2.863018	-6.299465	-4.451871
93	8	0	-9.725493	2.549033	0.760152
94	1	0	-10.393867	2.165359	1.337882
95	1	0	-10.040616	3.428508	0.526704

SCF Done: E(RB3LYP) = -2351.90788907   A.U. after   1 cycles

Harmonic frequencies (cm**-1), IR intensities (KM/Mole)

	1	2	3
	A	A	A
Frequencies --	-283.8006	8.0140	9.9818

Red. masses -- 4.8120 5.0262 5.4204
Frc consts -- 0.2284 0.0002 0.0003
IR Inten -- 664.5706 0.1532 0.8086

Zero-point correction= 0.792490 (a.u.)
Thermal correction to Energy= 0.874934
Thermal correction to Enthalpy= 0.875878
Thermal correction to Gibbs Free Energy= 0.651737
Sum of electronic and zero-point Energies= -2351.115399
Sum of electronic and thermal Energies= -2351.032956
Sum of electronic and thermal Enthalpies= -2351.032011
Sum of electronic and thermal Free Energies= -2351.256152

	E (Thermal)	CV	S
	KCal/Mol	Cal/Mol-Kelvin	Cal/Mol-Kelvin
Total	549.029	266.007	471.743

==TS2(IV, [1,3]OH shift), OH rearrangement to the ortho carbon by B3LYP/6-31G(d)==

Stoichiometry C6H61NO27(2+) bamb238.log

Standard orientation:

Center Number	Atomic Number	Atomic Type	Coordinates (Angstroms) X	Y	Z
1	6	0	0.550732	0.013724	-1.711572
2	7	0	1.822115	0.261001	-2.028630
3	6	0	0.118666	-1.354081	-1.574670
4	6	0	-0.387986	1.100566	-1.584500
5	6	0	-1.217297	-1.612417	-1.411361
6	6	0	-1.732908	0.806033	-1.463009
7	6	0	-2.142028	-0.533866	-1.376535
8	1	0	2.431894	-0.561721	-2.333397
9	1	0	0.860316	-2.143416	-1.647180
10	1	0	-0.041486	2.129206	-1.633089
11	1	0	-1.588905	-2.628190	-1.307623
12	1	0	-2.473467	1.600732	-1.397401
13	1	0	-3.199644	-0.760115	-1.272109
14	8	0	3.151952	0.635699	-0.511688
15	1	0	2.559281	0.320148	0.472639
16	1	0	2.111594	1.233493	-2.282655
17	8	0	1.889842	0.018795	1.453830
18	1	0	1.202569	0.768439	1.492576
19	1	0	2.469203	0.011275	2.268191
20	8	0	0.147168	1.831149	1.040012
21	1	0	-0.760092	1.829167	1.467201
22	1	0	0.331811	2.721543	0.656988
23	8	0	3.175123	-1.978384	-2.557479
24	1	0	3.810135	-1.991166	-1.790935
25	1	0	3.737838	-2.112636	-3.346918
26	8	0	4.917559	-1.355146	-0.652190
27	1	0	3.883905	-0.048162	-0.620324
28	1	0	5.139267	-1.605720	0.295900
29	8	0	2.790710	2.823456	-2.009330
30	1	0	3.458501	3.266013	-2.589094

31	1	0	3.279553	2.354880	-1.298102
32	1	0	5.769406	-1.293887	-1.153168
33	8	0	5.504368	-1.710555	1.901976
34	1	0	5.415773	-2.569197	2.354583
35	1	0	4.929008	-1.097269	2.420261
36	8	0	-2.212931	1.603133	2.224325
37	1	0	-2.807503	2.375072	2.316053
38	1	0	-2.800296	0.967689	1.755653
39	8	0	-4.591736	3.038590	1.881169
40	1	0	-4.873743	2.100890	1.855944
41	1	0	-5.270258	3.512228	2.413468
42	8	0	-4.356137	0.462878	0.882999
43	1	0	-4.448573	1.047165	0.092990
44	1	0	-4.688805	-0.424237	0.604917
45	8	0	3.704740	-0.214809	3.393180
46	1	0	4.038522	0.591959	3.855956
47	1	0	3.524888	-0.884892	4.103967
48	8	0	0.660941	3.953130	-0.580444
49	1	0	0.580025	4.929115	-0.508057
50	1	0	1.498471	3.781068	-1.057672
51	8	0	-5.039944	-1.865014	-0.360780
52	1	0	-5.900286	-2.323275	-0.304841
53	1	0	-4.386354	-2.603018	-0.364844
54	8	0	-4.360766	2.588991	-0.849364
55	1	0	-5.185452	2.837261	-1.320440
56	1	0	-4.416636	3.052212	0.017460
57	8	0	-6.765473	3.439293	-2.048987
58	1	0	-6.688628	4.022095	-2.819675
59	1	0	-7.438386	2.787616	-2.297649
60	8	0	-3.360289	-4.062346	-0.639528
61	1	0	-3.567057	-4.591000	0.155076
62	1	0	-3.886002	-4.516379	-1.330171
63	8	0	-6.984193	-3.953254	-0.153927
64	1	0	-6.503231	-4.480234	0.525781
65	1	0	-7.921847	-3.975329	0.092291
66	8	0	-6.608274	4.211632	3.402423
67	1	0	-6.405976	4.442425	4.321847
68	1	0	-7.087702	4.977394	3.051465
69	8	0	-5.141744	-5.712236	0.890733
70	1	0	-5.234673	-6.115642	-0.000338
71	1	0	-5.181573	-6.436452	1.534309
72	8	0	-5.479236	-5.547216	-1.787807
73	1	0	-6.214137	-4.945454	-1.527116
74	1	0	-5.759540	-6.010347	-2.591620
75	8	0	3.551532	-2.176182	5.248627
76	1	0	2.708328	-2.589151	5.487042
77	1	0	4.101224	-2.889914	4.863089
78	8	0	4.605044	2.038048	4.709222
79	1	0	4.096502	2.277384	5.499542
80	1	0	5.528301	2.017761	5.005133
81	8	0	5.373016	-3.862953	3.868789
82	1	0	5.279684	-4.819551	3.731070
83	1	0	6.249471	-3.751016	4.272774
84	8	0	6.993396	-1.499813	-2.385492
85	1	0	6.559381	-1.695021	-3.238994
86	1	0	7.684958	-0.848333	-2.571939
87	8	0	5.283672	-2.436101	-4.483084

88	1	0	5.470610	-3.389519	-4.505152
89	1	0	5.283006	-2.159806	-5.414213
90	8	0	4.675472	4.037739	-3.612162
91	1	0	4.326782	4.446792	-4.419799
92	1	0	5.184754	4.742210	-3.181306
93	8	0	0.587422	6.750472	-0.453503
94	1	0	-0.190292	7.166060	-0.856820
95	1	0	0.635840	7.132013	0.436615

SCF Done: E(RB+HF-LYP) = -2350.63201116 A.U. after 1 cycles

Harmonic frequencies (cm**-1), IR intensities (KM/Mole)

	1	2	3
	A	A	A
Frequencies --	-574.5941	8.8945	10.6866
Red. masses --	1.5968	5.3569	5.2755
Frc consts --	0.3106	0.0002	0.0004
IR Inten --	1807.2488	0.2401	1.1820

Zero-point correction= 0.795444 (a.u.)
Thermal correction to Energy= 0.874542
Thermal correction to Enthalpy= 0.875487
Thermal correction to Gibbs Free Energy= 0.661067
Sum of electronic and zero-point Energies= -2349.836568
Sum of electronic and thermal Energies= -2349.757469
Sum of electronic and thermal Enthalpies= -2349.756525
Sum of electronic and thermal Free Energies= -2349.970944

	E (Thermal)	CV	S
	KCal/Mol	Cal/Mol-Kelvin	Cal/Mol-Kelvin
Total	548.784	260.564	451.284

==TS2(IV, [1,3]OH shift) by B3LYP/6-311+G(d,p)==

Stoichiometry C6H61NO27(2+) bamb238.high.log

Standard orientation:

Center Number	Atomic Number	Atomic Type	Coordinates (Angstroms) X	Y	Z
1	6	0	-0.601322	-0.172791	-1.745436
2	7	0	-1.862383	-0.497779	-2.014769
3	6	0	-0.230306	1.217611	-1.717880
4	6	0	0.383044	-1.201888	-1.542518
5	6	0	1.088985	1.548319	-1.564683
6	6	0	1.704577	-0.842506	-1.424440
7	6	0	2.053668	0.520215	-1.435463
8	1	0	-2.512806	0.256866	-2.380970
9	1	0	-1.003144	1.965989	-1.847855
10	1	0	0.081519	-2.243434	-1.502234
11	1	0	1.411419	2.583350	-1.537209
12	1	0	2.480230	-1.592224	-1.301076
13	1	0	3.097190	0.798395	-1.331303
14	8	0	-3.149129	-0.777679	-0.427468
15	1	0	-2.535811	-0.490024	0.542468

16	1	0	-2.121830	-1.494569	-2.182494
17	8	0	-1.847212	-0.210645	1.514820
18	1	0	-1.157403	-0.941841	1.631347
19	1	0	-2.368047	-0.052192	2.342036
20	8	0	-0.090362	-2.056521	1.455181
21	1	0	0.843958	-1.986840	1.771413
22	1	0	-0.215450	-2.883160	0.952603
23	8	0	-3.376283	1.590891	-2.731500
24	1	0	-3.973398	1.693647	-1.953290
25	1	0	-3.937640	1.727432	-3.512296
26	8	0	-4.989813	1.216855	-0.615236
27	1	0	-3.871190	-0.101671	-0.531895
28	1	0	-5.127589	1.583135	0.294418
29	8	0	-2.794351	-3.089645	-1.886736
30	1	0	-3.345539	-3.646196	-2.476188
31	1	0	-3.375189	-2.693856	-1.216899
32	1	0	-5.861614	1.098563	-1.041999
33	8	0	-5.283811	1.879331	1.961353
34	1	0	-5.352168	2.717455	2.440703
35	1	0	-4.721588	1.299888	2.513439
36	8	0	2.421142	-1.673672	2.318021
37	1	0	3.060082	-2.394050	2.440493
38	1	0	2.959022	-0.990295	1.873876
39	8	0	4.979324	-2.934228	1.995442
40	1	0	5.263322	-2.008506	2.034072
41	1	0	5.608672	-3.442729	2.539471
42	8	0	4.447378	-0.286947	0.940530
43	1	0	4.565515	-0.865489	0.162333
44	1	0	4.641482	0.621751	0.640496
45	8	0	-3.489260	0.385820	3.559730
46	1	0	-3.843611	-0.342850	4.108083
47	1	0	-3.263577	1.118727	4.171229
48	8	0	-0.537048	-4.101468	-0.411415
49	1	0	-0.343673	-5.050622	-0.303305
50	1	0	-1.382840	-4.036002	-0.883122
51	8	0	4.810804	2.162667	-0.359093
52	1	0	5.631259	2.678490	-0.316165
53	1	0	4.120213	2.844158	-0.473871
54	8	0	4.495406	-2.484991	-0.772370
55	1	0	5.188655	-2.764088	-1.395369
56	1	0	4.666510	-2.962064	0.060159
57	8	0	6.525965	-3.302566	-2.597010
58	1	0	6.495604	-4.044792	-3.209391
59	1	0	7.382894	-2.883487	-2.727497
60	8	0	3.003569	4.267954	-0.907564
61	1	0	3.086053	4.858546	-0.144496
62	1	0	3.524451	4.723831	-1.591969
63	8	0	6.650782	4.442655	-0.253361
64	1	0	6.154755	5.031884	0.345905
65	1	0	7.572671	4.475014	0.024867
66	8	0	6.824081	-4.316786	3.615503
67	1	0	6.712134	-4.449820	4.562701
68	1	0	7.495413	-4.946992	3.334017
69	8	0	4.675482	6.303029	0.523837
70	1	0	4.751700	6.647647	-0.381930
71	1	0	4.627069	7.063284	1.113487
72	8	0	5.106763	5.772443	-2.181645

73	1	0	5.874412	5.266500	-1.855911
74	1	0	5.338537	6.122038	-3.048430
75	8	0	-3.264834	2.649106	5.083657
76	1	0	-2.585030	3.091676	5.600210
77	1	0	-3.887123	3.328017	4.774820
78	8	0	-4.475668	-1.737343	5.077713
79	1	0	-3.945650	-2.196723	5.738092
80	1	0	-5.391015	-1.821036	5.365824
81	8	0	-5.378202	4.197248	3.867493
82	1	0	-5.297362	5.111239	3.568740
83	1	0	-6.200870	4.163473	4.371981
84	8	0	-7.061854	1.042522	-2.409864
85	1	0	-6.757654	1.333602	-3.283220
86	1	0	-7.948469	0.684816	-2.514548
87	8	0	-5.530459	2.080861	-4.688406
88	1	0	-5.687121	3.023552	-4.828972
89	1	0	-5.567814	1.683019	-5.567190
90	8	0	-4.343903	-4.676233	-3.556394
91	1	0	-4.048032	-4.989453	-4.418280
92	1	0	-4.943547	-5.352745	-3.222240
93	8	0	-0.060548	-6.866646	-0.097776
94	1	0	0.653685	-7.346798	-0.530248
95	1	0	-0.213471	-7.318842	0.738944

SCF Done: E(RB3LYP) = -2351.89500025   A.U. after   18 cycles
Convg =   0.5481D-08   -V/T = 2.0044

Harmonic frequencies (cm**-1), IR intensities (KM/Mole)

	1	2	3
	A	A	A
Frequencies --	-584.0190	-25.7542	7.3645
Red. masses --	1.4527	8.1832	5.2773
Frc consts --	0.2919	0.0032	0.0002
IR Inten --	2182.0026	10.3731	1.1850

Zero-point correction= 0.790228 (a.u.)
Thermal correction to Energy= 0.872568
Thermal correction to Enthalpy= 0.873512
Thermal correction to Gibbs Free Energy= 0.650347
Sum of electronic and zero-point Energies= -2351.104772
Sum of electronic and thermal Energies= -2351.022432
Sum of electronic and thermal Enthalpies= -2351.021488
Sum of electronic and thermal Free Energies= -2351.244654

	E (Thermal)	CV	S
	KCal/Mol	Cal/Mol-Kelvin	Cal/Mol-Kelvin
Total	547.545	267.057	469.691

[4] TS geometries in Figure 6

== TS2(V), para Cl- addition TS by B3LYP/6-31G(d)==

Stoichiometry   C6H39ClNO16(1+)   bambclp.log

Standard orientation:

Center Number	Atomic Number	Atomic Type	Coordinates (Angstroms) X	Y	Z
1	6	0	-1.724023	-2.168872	-0.026533
2	7	0	-3.013851	-2.346309	0.225939
3	6	0	-1.310566	-1.617983	-1.288601
4	6	0	-0.764231	-2.397995	1.019543
5	6	0	-0.008652	-1.242038	-1.460894
6	6	0	0.528346	-2.001853	0.834388
7	6	0	0.911360	-1.368430	-0.382946
8	1	0	-3.705305	-2.231022	-0.550969
9	1	0	-2.060709	-1.474057	-2.060482
10	1	0	-1.112559	-2.816047	1.959678
11	1	0	0.335421	-0.792288	-2.385806
12	1	0	1.262938	-2.106859	1.623467
13	1	0	1.813359	-0.783215	-0.390151
14	8	0	-3.792296	-0.706508	1.322716
15	1	0	-2.637144	0.310085	1.149357
16	1	0	-3.307600	-2.817636	1.107226
17	1	0	-0.888862	1.343062	1.658488
18	8	0	-1.896814	0.973470	0.869131
19	1	0	-2.336046	1.811676	0.601398
20	8	0	-0.028361	1.830240	2.191705
21	1	0	0.864441	1.351848	1.978903
22	1	0	0.043555	2.744181	1.792161
23	8	0	-4.585700	-1.774633	-1.935344
24	1	0	-5.068278	-0.958359	-1.646779
25	1	0	-5.239604	-2.360548	-2.344759
26	8	0	-5.519842	0.444399	-0.733613
27	1	0	-4.510211	-0.365301	0.746322
28	1	0	-5.104954	1.279311	-1.093037
29	8	0	-3.637219	-2.863874	2.810358
30	1	0	-4.402103	-3.313605	3.199305
31	1	0	-3.919716	-1.935586	2.607714
32	1	0	-6.457764	0.655863	-0.609499
33	8	0	-4.463987	2.721188	-1.677835
34	1	0	-4.135416	2.720601	-2.588749
35	1	0	-3.749850	3.117623	-1.132179
36	8	0	2.157587	0.732515	1.481812
37	1	0	2.484451	1.100585	0.618475
38	1	0	2.965839	0.530537	2.008876
39	8	0	4.659041	0.031875	2.370119
40	1	0	4.995184	-0.160576	3.258529
41	1	0	4.866746	-0.756891	1.809507
42	8	0	3.130717	1.658042	-0.880988
43	1	0	4.106919	1.577444	-0.665221
44	1	0	2.979558	1.061154	-1.660120
45	8	0	-2.649440	3.584149	0.239243
46	1	0	-3.161698	4.053848	0.915870
47	1	0	-1.726689	3.939432	0.306529
48	8	0	-0.031988	4.158709	0.827528
49	1	0	0.651794	4.098350	0.076114
50	1	0	0.176689	4.972206	1.313773
51	8	0	2.690568	-0.129155	-2.907331
52	1	0	2.870025	-1.043204	-2.584604
53	1	0	3.160972	-0.055092	-3.751824

54	8	0	5.622290	1.137443	-0.066737
55	1	0	5.718515	0.179819	-0.248587
56	1	0	5.516426	1.138607	0.904686
57	8	0	5.309795	-1.669770	0.337119
58	1	0	4.556243	-2.083388	-0.157003
59	1	0	6.003992	-2.347727	0.344363
60	8	0	1.687822	3.916594	-1.117144
61	1	0	2.288788	3.123026	-1.060109
62	1	0	2.255384	4.664954	-1.352518
63	17	0	2.923144	-2.854300	-1.323269

SCF Done: E(RB+HF-LYP) = -1970.40530053   A.U. after   1 cycles

Harmonic frequencies (cm**-1), IR intensities (KM/Mole)

	1	2	3
	A	A	A
Frequencies --	-171.9309	20.6354	22.9775
Red. masses --	5.3129	5.7769	5.5756
Frc consts --	0.0925	0.0014	0.0017
IR Inten --	328.6494	0.7137	0.3602

Zero-point correction= 0.524193 (a.u.)
Thermal correction to Energy= 0.569869
Thermal correction to Enthalpy= 0.570814
Thermal correction to Gibbs Free Energy= 0.443487
Sum of electronic and zero-point Energies= -1969.881107
Sum of electronic and thermal Energies= -1969.835431
Sum of electronic and thermal Enthalpies= -1969.834487
Sum of electronic and thermal Free Energies= -1969.961813

	E (Thermal)	CV	S
	KCal/Mol	Cal/Mol-Kelvin	Cal/Mol-Kelvin
Total	357.598	159.803	267.981

==TS2(V) by B3LYP/6-311+G(d,p)==

Stoichiometry   C6H39ClNO16(1+) bambclp.high.log

Standard orientation:

Center Number	Atomic Number	Atomic Type	Coordinates (Angstroms) X	Y	Z
1	6	0	-1.601264	-2.125856	-0.083243
2	7	0	-2.894872	-2.403666	0.092552
3	6	0	-1.181487	-1.435554	-1.262574
4	6	0	-0.671633	-2.403163	0.967136
5	6	0	0.109691	-1.001190	-1.360136
6	6	0	0.618576	-1.967326	0.851865
7	6	0	1.011341	-1.228963	-0.291488
8	1	0	-3.541325	-2.345476	-0.719527
9	1	0	-1.909840	-1.241868	-2.041312
10	1	0	-1.024035	-2.918261	1.853653
11	1	0	0.455148	-0.457734	-2.229688
12	1	0	1.339515	-2.135026	1.641754
13	1	0	1.942679	-0.691892	-0.267477

14	8	0	-3.818696	-0.927837	1.113353
15	1	0	-2.734989	0.363369	1.290079
16	1	0	-3.178865	-2.995990	0.888348
17	1	0	-0.816906	1.571395	1.901421
18	8	0	-2.135269	1.158379	1.309692
19	1	0	-2.616843	1.922286	0.956640
20	8	0	0.020493	2.076131	2.227323
21	1	0	0.960172	1.619559	2.006138
22	1	0	0.006629	2.977006	1.806986
23	8	0	-4.508154	-1.964682	-2.086466
24	1	0	-5.071982	-1.187362	-1.887561
25	1	0	-4.965258	-2.514167	-2.730082
26	8	0	-5.620622	0.260465	-0.996400
27	1	0	-4.513744	-0.622536	0.500889
28	1	0	-5.194672	1.106126	-1.267255
29	8	0	-3.650762	-3.153654	2.612172
30	1	0	-4.239666	-3.662305	3.177094
31	1	0	-4.025851	-2.259156	2.500132
32	1	0	-6.547070	0.461384	-0.827497
33	8	0	-4.334138	2.539327	-1.740952
34	1	0	-4.352820	2.970753	-2.599999
35	1	0	-3.731406	3.048784	-1.168842
36	8	0	2.246870	1.195184	1.695492
37	1	0	2.641640	1.387859	0.814526
38	1	0	2.704032	0.416346	2.088811
39	8	0	3.504415	-1.050471	2.564696
40	1	0	3.883261	-1.147396	3.444034
41	1	0	4.173078	-1.386684	1.929853
42	8	0	3.326147	1.781493	-0.765657
43	1	0	4.283495	1.640869	-0.588359
44	1	0	3.140501	1.223876	-1.558280
45	8	0	-2.805129	3.778735	0.272440
46	1	0	-3.348356	4.402926	0.766785
47	1	0	-1.885123	4.104209	0.342147
48	8	0	-0.082289	4.395700	0.862271
49	1	0	0.637134	4.329554	0.172552
50	1	0	0.102487	5.202618	1.357607
51	8	0	2.844389	0.098807	-2.880188
52	1	0	2.993852	-0.834669	-2.606391
53	1	0	3.292927	0.203497	-3.725739
54	8	0	5.818365	0.848313	-0.226648
55	1	0	5.759797	-0.097594	0.009070
56	1	0	6.688221	1.163609	0.033067
57	8	0	5.187835	-1.824990	0.482374
58	1	0	4.531742	-2.151605	-0.188922
59	1	0	5.846738	-2.525623	0.553305
60	8	0	1.899752	4.108195	-0.859579
61	1	0	2.497753	3.324424	-0.864396
62	1	0	2.158715	4.676010	-1.590673
63	17	0	3.003902	-2.687995	-1.495383

SCF Done: E(RB+HF-LYP) = -1971.20633072   A.U. after   1 cycles

Harmonic frequencies (cm**-1), IR intensities (KM/Mole)

1	2	3
A	A	A

Frequencies -- -132.7803 18.3802 23.4875
Red. masses -- 6.3288 6.5274 6.3339
Frc consts -- 0.0657 0.0013 0.0021
IR Inten -- 488.2318 0.1110 1.8621

Zero-point correction= 0.521579 (a.u.)
Thermal correction to Energy= 0.570260
Thermal correction to Enthalpy= 0.571204
Thermal correction to Gibbs Free Energy= 0.435200
Sum of electronic and zero-point Energies= -1970.684751
Sum of electronic and thermal Energies= -1970.636071
Sum of electronic and thermal Enthalpies= -1970.635127
Sum of electronic and thermal Free Energies= -1970.771131

	E (Thermal)	CV	S
	KCal/Mol	Cal/Mol-Kelvin	Cal/Mol-Kelvin
Total	357.843	165.210	286.244

==TS2(V, [1,3]OH shift) by B3LYP/6-31G(d)==

Stoichiometry C6H39ClNO16(1+) bambclo.log

Standard orientation:

Center Number	Atomic Number	Atomic Type	Coordinates (Angstroms) X	Y	Z
1	6	0	1.900344	-1.458485	-0.491098
2	7	0	1.600737	-0.157259	-0.394938
3	6	0	0.875244	-2.447187	-0.379834
4	6	0	3.264665	-1.850391	-0.711175
5	6	0	1.194333	-3.765924	-0.555675
6	6	0	3.540409	-3.212848	-0.959905
7	6	0	2.532285	-4.146498	-0.872529
8	1	0	0.717933	0.132871	0.057962
9	1	0	-0.138698	-2.126961	-0.157149
10	1	0	3.995810	-1.091512	-0.959290
11	1	0	0.432811	-4.534908	-0.469158
12	1	0	4.556249	-3.511833	-1.191976
13	1	0	2.751994	-5.195038	-1.050134
14	8	0	1.099573	0.611013	-2.118992
15	1	0	-0.233132	0.880570	-2.288460
16	1	0	2.368932	0.535829	-0.213888
17	1	0	-1.544209	2.149434	-1.266955
18	8	0	-1.287693	1.173747	-2.411188
19	1	0	-1.360332	1.572181	-3.293905
20	8	0	-1.755766	2.595134	-0.349804
21	1	0	-2.804795	2.357809	-0.179543
22	1	0	-1.212492	2.019514	0.328737
23	8	0	3.297738	1.892457	0.252330
24	1	0	3.198211	2.487457	-0.530476
25	1	0	4.258099	1.610023	0.333928
26	8	0	2.178191	3.227491	-1.782734
27	1	0	1.548887	1.489828	-2.100874
28	1	0	1.542948	3.765409	-1.222908
29	8	0	5.672311	0.757839	0.321309
30	1	0	5.395803	-0.025863	0.858240

31	1	0	6.472380	1.098074	0.750507
32	1	0	2.548009	3.830573	-2.445277
33	8	0	-0.388762	1.080228	1.165529
34	1	0	0.261262	1.617620	1.693288
35	1	0	-0.975949	0.539375	1.792171
36	8	0	-4.093486	1.815713	-0.126088
37	1	0	-4.367578	1.490922	0.765967
38	1	0	-4.004567	0.996396	-0.679493
39	8	0	-3.378381	-0.395646	-1.582808
40	1	0	-2.624053	0.031018	-2.063784
41	1	0	-2.984966	-1.049956	-0.962744
42	8	0	-4.688589	0.458928	2.197025
43	1	0	-5.290455	0.813793	2.870237
44	1	0	-5.107675	-0.368087	1.848307
45	8	0	0.564750	4.459790	-0.065231
46	1	0	0.858349	4.090018	0.796401
47	1	0	-0.370630	4.204938	-0.147164
48	8	0	-2.018259	-0.404700	2.563094
49	1	0	-2.142047	-1.182877	1.981246
50	1	0	-2.918157	-0.020739	2.636044
51	8	0	-5.397073	-1.891593	0.975835
52	1	0	-5.728088	-1.729294	0.036052
53	1	0	-5.993020	-2.542258	1.379394
54	8	0	-5.935135	-1.313214	-1.529650
55	1	0	-6.247892	-1.953722	-2.185532
56	1	0	-5.065020	-0.980030	-1.849816
57	8	0	1.507934	2.798774	1.997736
58	1	0	1.806846	2.988628	2.900758
59	1	0	2.308910	2.480373	1.489583
60	8	0	-2.759319	-2.198057	0.486671
61	1	0	-2.454413	-3.110209	0.361638
62	1	0	-3.721901	-2.261919	0.726927
63	17	0	4.133737	-1.555992	1.664278

SCF Done: E(RB+HF-LYP) = -1970.41831162 A.U. after 1 cycles

Harmonic frequencies (cm**-1), IR intensities (KM/Mole)
activities (A**4/AMU), depolarization ratios for plane and unpolarized
incident light, reduced masses (AMU), force constants (mDyne/A),
and normal coordinates:

	1	2	3
	A	A	A
Frequencies --	-327.7209	16.0161	23.9022
Red. masses --	4.4522	6.0065	7.6369
Frc consts --	0.2817	0.0009	0.0026
IR Inten --	2213.5185	2.6707	8.7229

Zero-point correction= 0.526859 (a.u.)
Thermal correction to Energy= 0.569969
Thermal correction to Enthalpy= 0.570913
Thermal correction to Gibbs Free Energy= 0.450185
Sum of electronic and zero-point Energies= -1969.891453
Sum of electronic and thermal Energies= -1969.848343
Sum of electronic and thermal Enthalpies= -1969.847399
Sum of electronic and thermal Free Energies= -1969.968127

	E (Thermal)	CV	S
	KCal/Mol	Cal/Mol-Kelvin	Cal/Mol-Kelvin
Total	357.661	153.911	254.093

==TS2(V, [1,3]OH shift) by B3LYP/6-311+G(d,p)==

Stoichiometry C6H39ClNO16(1+) bambclo.high.log

Standard orientation:

Center Number	Atomic Number	Atomic Type	Coordinates (Angstroms) X	Y	Z
1	6	0	1.698483	-1.560268	-0.549689
2	7	0	1.621713	-0.222844	-0.420619
3	6	0	0.518412	-2.355221	-0.524854
4	6	0	2.976107	-2.170995	-0.726536
5	6	0	0.613185	-3.703633	-0.733607
6	6	0	3.032055	-3.550768	-0.993469
7	6	0	1.877718	-4.299684	-0.990339
8	1	0	0.787874	0.196152	0.006717
9	1	0	-0.437424	-1.877833	-0.342022
10	1	0	3.845223	-1.550021	-0.881854
11	1	0	-0.273252	-4.325849	-0.719920
12	1	0	3.992007	-4.014214	-1.177416
13	1	0	1.927561	-5.364033	-1.187697
14	8	0	1.316110	0.632718	-2.075948
15	1	0	-0.170465	0.855589	-2.482286
16	1	0	2.486327	0.306256	-0.173256
17	1	0	-1.499537	2.202204	-1.582542
18	8	0	-1.155371	1.049003	-2.672711
19	1	0	-1.218431	1.256863	-3.612348
20	8	0	-1.716853	2.712999	-0.739347
21	1	0	-2.708237	2.483687	-0.500271
22	1	0	-1.106483	2.328712	-0.008632
23	8	0	3.654495	1.524102	0.323625
24	1	0	3.797330	2.085365	-0.461490
25	1	0	4.520632	1.116490	0.600695
26	8	0	2.980628	3.035199	-1.877050
27	1	0	1.882792	1.430757	-2.041870
28	1	0	2.521071	3.772043	-1.414277
29	8	0	5.775351	0.117800	0.999834
30	1	0	5.317173	-0.683821	1.348807
31	1	0	6.544830	0.271756	1.556290
32	1	0	3.428990	3.409384	-2.642471
33	8	0	-0.275313	1.513214	0.997804
34	1	0	0.422527	1.999907	1.491126
35	1	0	-0.900658	1.080771	1.640077
36	8	0	-4.066773	1.944199	-0.332678
37	1	0	-4.430479	1.809110	0.564492
38	1	0	-4.008968	1.054170	-0.736945
39	8	0	-3.342937	-0.489074	-1.519446
40	1	0	-2.572944	-0.190310	-2.038030
41	1	0	-3.020297	-1.103870	-0.835390
42	8	0	-4.919650	1.032327	2.186623
43	1	0	-5.485006	1.499608	2.811778
44	1	0	-5.360308	0.181074	1.982799

45	8	0	1.627135	4.692533	-0.194586
46	1	0	1.717773	4.288156	0.688081
47	1	0	1.600179	5.646267	-0.071876
48	8	0	-2.126910	0.246254	2.415103
49	1	0	-2.302147	-0.627709	2.025746
50	1	0	-3.006850	0.638026	2.543801
51	8	0	-5.721890	-1.489925	1.259501
52	1	0	-5.996215	-1.447610	0.309161
53	1	0	-6.347747	-2.072112	1.705245
54	8	0	-5.998041	-1.347856	-1.397543
55	1	0	-6.693801	-1.214206	-2.047753
56	1	0	-5.155213	-1.058236	-1.789273
57	8	0	1.948222	2.861999	1.922506
58	1	0	2.197306	2.973544	2.846772
59	1	0	2.684095	2.364719	1.481754
60	8	0	-3.016999	-2.033041	0.884981
61	1	0	-2.718841	-2.935462	1.045773
62	1	0	-3.975215	-2.016796	1.099326
63	17	0	3.786910	-2.081459	1.741713

SCF Done: E(RB+HF-LYP) = -1971.20372386   A.U. after   1 cycles

Harmonic frequencies (cm**-1), IR intensities (KM/Mole)

	1	2	3
	A	A	A
Frequencies --	-203.2455	13.7956	23.3256
Red. masses --	7.8526	6.2256	6.3003
Frc consts --	0.1911	0.0007	0.0020
IR Inten --	816.5038	2.3190	2.3572

Zero-point correction= 0.526117 (a.u.)
Thermal correction to Energy= 0.572349
Thermal correction to Enthalpy= 0.573293
Thermal correction to Gibbs Free Energy= 0.444048
Sum of electronic and zero-point Energies= -1970.677607
Sum of electronic and thermal Energies= -1970.631375
Sum of electronic and thermal Enthalpies= -1970.630431
Sum of electronic and thermal Free Energies= -1970.759676

	E (Thermal)	CV	S
	KCal/Mol	Cal/Mol-Kelvin	Cal/Mol-Kelvin
Total	359.154	160.951	272.019

[5] TS geometries in Figure S2

==TS2(2H2O) by B3LYP\6-31G(d)==

Stoichiometry   C6H12NO3(1+) bamb04.log

Standard orientation:

Center Number	Atomic Number	Atomic Type	Coordinates (Angstroms) X	Y	Z
1	6	0	-0.703837	-1.109829	0.054970
2	7	0	-2.032676	-1.040556	0.082675

3	6	0	-0.024685	-1.075084	-1.213693
4	6	0	0.044687	-1.016185	1.279402
5	6	0	1.301950	-0.802055	-1.260645
6	6	0	1.372835	-0.743965	1.233833
7	6	0	2.008770	-0.437451	-0.039999
8	1	0	-2.582316	-1.184503	-0.758586
9	1	0	-0.590209	-1.265318	-2.122013
10	1	0	-0.462757	-1.168866	2.228760
11	1	0	1.838883	-0.777469	-2.202325
12	1	0	1.963678	-0.698656	2.142927
13	1	0	3.092898	-0.482375	-0.075826
14	8	0	-2.664121	0.846078	-0.223597
15	1	0	-3.267160	1.074576	0.510133
16	1	0	-2.536826	-1.181135	0.953004
17	1	0	-1.265751	1.708930	0.018319
18	8	0	-0.413976	2.234507	0.165683
19	1	0	-0.552363	3.082574	-0.285057
20	1	0	0.974561	1.701606	-0.073930
21	8	0	1.973673	1.394461	-0.170215
22	1	0	2.453172	1.781576	0.587694

SCF Done: E(RB+HF-LYP) = -515.911680696 A.U. after 1 cycles

Harmonic frequencies (cm**-1), IR intensities (KM/Mole)

	1	2	3
	A	A	A
Frequencies --	-298.9200	40.3529	101.8196
Red. masses --	6.0348	4.0645	3.9931
Frc consts --	0.3177	0.0039	0.0244
IR Inten --	279.4721	2.5154	3.8847

Zero-point correction= 0.182065 (a.u.)
Thermal correction to Energy= 0.193329
Thermal correction to Enthalpy= 0.194273
Thermal correction to Gibbs Free Energy= 0.145587
Sum of electronic and zero-point Energies= -515.729616
Sum of electronic and thermal Energies= -515.718351
Sum of electronic and thermal Enthalpies= -515.717407
Sum of electronic and thermal Free Energies= -515.766094

	E (Thermal)	CV	S
	KCal/Mol	Cal/Mol-Kelvin	Cal/Mol-Kelvin
Total	121.316	43.290	102.469

==TS2(2H2O) by B3LYP/6-311+G(d,p)==

Stoichiometry C6H12NO3(1+) bamb04.high.log

Standard orientation:

Center Number	Atomic Number	Atomic Type	Coordinates (Angstroms) X	Y	Z
1	6	0	-0.628837	-1.077896	0.129637
2	7	0	-1.941036	-1.166144	0.217866
3	6	0	-0.025570	-1.013915	-1.177963
4	6	0	0.181065	-0.986467	1.316895

5	6	0	1.316956	-0.811747	-1.279302
6	6	0	1.524877	-0.794565	1.189349
7	6	0	2.092665	-0.646291	-0.101250
8	1	0	-2.547145	-1.001257	-0.600173
9	1	0	-0.657311	-1.102155	-2.053635
10	1	0	-0.291303	-1.058751	2.289725
11	1	0	1.794845	-0.731079	-2.246288
12	1	0	2.156752	-0.711923	2.063875
13	1	0	3.158749	-0.488502	-0.194123
14	8	0	-2.735208	0.730962	-0.580882
15	1	0	-3.636783	1.007707	-0.351556
16	1	0	-2.403613	-1.202201	1.121701
17	1	0	-1.466692	1.702325	0.115422
18	8	0	-0.662114	2.009361	0.601253
19	1	0	-0.933701	2.768488	1.128441
20	1	0	1.018113	2.020885	-0.058292
21	8	0	1.913017	1.813185	-0.406939
22	1	0	2.502855	2.516701	-0.111813

SCF Done: E(RB+HF-LYP) = -516.117893260 A.U. after 1 cycles

Harmonic frequencies (cm**-1), IR intensities (KM/Mole)

	1	2	3
	A	A	A
Frequencies --	-508.9580	59.1509	72.4870
Red. masses --	1.1710	5.1227	3.7903
Frc consts --	0.1787	0.0106	0.0117
IR Inten --	339.0135	1.3624	0.3880

Zero-point correction= 0.178582 (a.u.)
Thermal correction to Energy= 0.191624
Thermal correction to Enthalpy= 0.192568
Thermal correction to Gibbs Free Energy= 0.139855
Sum of electronic and zero-point Energies= -515.939311
Sum of electronic and thermal Energies= -515.926269
Sum of electronic and thermal Enthalpies= -515.925325
Sum of electronic and thermal Free Energies= -515.978038

	E (Thermal)	CV	S
	KCal/Mol	Cal/Mol-Kelvin	Cal/Mol-Kelvin
Total	120.246	47.563	110.943

==TS2(3H2O) by B3LYP\6-31G(d)==

Stoichiometry C6H14NO4(1+) bamb29bw.log

Standard orientation:

Center Number	Atomic Number	Atomic Type	Coordinates (Angstroms) X	Y	Z
1	6	0	0.479715	-1.269232	0.141254
2	7	0	1.789831	-1.497226	0.020989
3	6	0	-0.037564	-0.705771	1.359245
4	6	0	-0.403156	-1.512426	-0.968369
5	6	0	-1.351583	-0.381984	1.453675

6	6	0	-1.712526	-1.171877	-0.885940
7	6	0	-2.226173	-0.478009	0.291747
8	1	0	2.388025	-1.482159	0.840661
9	1	0	0.630968	-0.569067	2.203595
10	1	0	-0.014005	-2.001000	-1.858412
11	1	0	-1.762690	-0.018206	2.390118
12	1	0	-2.396223	-1.380290	-1.701370
13	1	0	-3.288296	-0.600557	0.487786
14	8	0	3.040427	-0.058115	-0.580153
15	1	0	2.372931	1.147368	0.298147
16	1	0	2.153673	-2.069514	-0.733956
17	8	0	1.763994	1.769892	0.809655
18	1	0	0.575732	2.072068	-0.235629
19	1	0	2.315663	2.503756	1.121671
20	8	0	-0.150842	2.041652	-0.939624
21	1	0	-1.480799	1.597063	-0.547413
22	1	0	-0.115807	2.870780	-1.440654
23	1	0	2.688948	0.129738	-1.471197
24	8	0	-2.436782	1.202359	-0.319859
25	1	0	-2.823586	1.750097	0.389908

SCF Done: E(RB+HF-LYP) = -592.348459833 A.U. after 1 cycles

Harmonic frequencies (cm**-1), IR intensities (KM/Mole)

	1	2	3
	A	A	A
Frequencies --	-272.3784	45.8040	58.9521
Red. masses --	6.3819	5.1166	4.6635
Frc consts --	0.2790	0.0063	0.0095
IR Inten --	381.2780	9.0112	1.6102

Zero-point correction= 0.207716 (a.u.)
Thermal correction to Energy= 0.221271
Thermal correction to Enthalpy= 0.222215
Thermal correction to Gibbs Free Energy= 0.168111
Sum of electronic and zero-point Energies= -592.140744
Sum of electronic and thermal Energies= -592.127189
Sum of electronic and thermal Enthalpies= -592.126245
Sum of electronic and thermal Free Energies= -592.180349

	E (Thermal)	CV	S
	KCal/Mol	Cal/Mol-Kelvin	Cal/Mol-Kelvin
Total	138.850	51.214	113.873

==TS2(3H2O) by B3LYP/6-311+G(d,p)==

Stoichiometry C6H14NO4(1+) bamb29bw.high.log

Standard orientation:

Center Number	Atomic Number	Atomic Type	Coordinates (Angstroms) X	Y	Z
1	6	0	0.542340	-1.300691	-0.086125
2	7	0	1.812359	-1.564849	-0.332473
3	6	0	0.137515	-0.939553	1.247783

4	6	0	-0.433994	-1.404218	-1.139603
5	6	0	-1.184388	-0.741083	1.511337
6	6	0	-1.750370	-1.215361	-0.853060
7	6	0	-2.144269	-0.864389	0.469771
8	1	0	2.575793	-1.442730	0.343692
9	1	0	0.890836	-0.845608	2.020290
10	1	0	-0.107703	-1.659452	-2.141643
11	1	0	-1.513235	-0.490392	2.511428
12	1	0	-2.505768	-1.309239	-1.621639
13	1	0	-3.196612	-0.843334	0.711940
14	8	0	3.238107	0.086550	-0.165760
15	1	0	2.347098	1.411358	0.421751
16	1	0	2.106395	-1.870714	-1.254917
17	8	0	1.639018	2.003194	0.776263
18	1	0	0.401526	2.267907	-0.384370
19	1	0	2.065664	2.687914	1.301108
20	8	0	-0.348852	2.189617	-1.020272
21	1	0	-1.856591	1.762884	-0.417654
22	1	0	-0.250136	2.890759	-1.672159
23	1	0	3.800805	0.166581	-0.950269
24	8	0	-2.700182	1.353781	-0.091216
25	1	0	-3.070311	1.954646	0.567018

SCF Done: E(RB+HF-LYP) = -592.596543771 A.U. after 1 cycles

Harmonic frequencies (cm**-1), IR intensities (KM/Mole)

	1	2	3
	A	A	A
Frequencies --	-448.3654	29.9524	40.5157
Red. masses --	1.2987	4.7737	5.5236
Frc consts --	0.1538	0.0025	0.0053
IR Inten --	280.0179	1.9589	3.0544

Zero-point correction= 0.204064 (a.u.)
Thermal correction to Energy= 0.219714
Thermal correction to Enthalpy= 0.220658
Thermal correction to Gibbs Free Energy= 0.160834
Sum of electronic and zero-point Energies= -592.392480
Sum of electronic and thermal Energies= -592.376830
Sum of electronic and thermal Enthalpies= -592.375886
Sum of electronic and thermal Free Energies= -592.435710

	E (Thermal)	CV	S
	KCal/Mol	Cal/Mol-Kelvin	Cal/Mol-Kelvin
Total	137.872	56.116	125.910

==TS([1,3]OH shift) by B3LYP/6-31G(d)==

Stoichiometry C6H12NO3(1+) bamb07.log

Standard orientation:

Center Number	Atomic Number	Atomic Type	Coordinates (Angstroms) X	Y	Z
1	6	0	-0.607482	-1.054508	0.054060

2	7	0	-1.942977	-1.088833	0.006020
3	6	0	0.181930	-1.394394	-1.086464
4	6	0	0.001491	-0.441452	1.219202
5	6	0	1.541710	-1.303182	-0.995360
6	6	0	1.424685	-0.450661	1.300346
7	6	0	2.162902	-0.851332	0.217269
8	1	0	-2.414444	-1.501387	-0.793999
9	1	0	-0.304476	-1.749939	-1.990237
10	1	0	-0.575470	-0.384189	2.135583
11	1	0	2.165270	-1.591889	-1.834695
12	1	0	1.900837	-0.119409	2.216395
13	1	0	3.247377	-0.847270	0.274624
14	8	0	-2.562845	0.739334	-0.529906
15	1	0	-3.441272	0.895486	-0.129195
16	1	0	-2.483061	-1.070347	0.865039
17	1	0	-1.407663	1.346240	0.305978
18	8	0	-0.553795	1.455288	0.863262
19	1	0	0.138152	1.840328	0.255811
20	8	0	1.330106	2.511511	-0.787800
21	1	0	0.991935	2.912228	-1.604828
22	1	0	1.844507	3.216088	-0.361380

SCF Done: E(RB+HF-LYP) = -515.916100615 A.U. after 1 cycles

Harmonic frequencies (cm**-1), IR intensities (KM/Mole)

	1	2	3
	A	A	A
Frequencies --	-346.2836	36.1678	54.7342
Red. masses --	5.6116	4.5631	4.7984
Frc consts --	0.3965	0.0035	0.0085
IR Inten --	94.9386	3.7514	3.9943

Zero-point correction= 0.181571 (a.u.)
Thermal correction to Energy= 0.193616
Thermal correction to Enthalpy= 0.194560
Thermal correction to Gibbs Free Energy= 0.143552
Sum of electronic and zero-point Energies= -515.734530
Sum of electronic and thermal Energies= -515.722485
Sum of electronic and thermal Enthalpies= -515.721540
Sum of electronic and thermal Free Energies= -515.772549

	E (Thermal)	CV	S
	KCal/Mol	Cal/Mol-Kelvin	Cal/Mol-Kelvin
Total	121.496	44.519	107.356

==TS2([1,3]OH shift) by B3LYP/6-311+G(d,p)==

Stoichiometry C6H12NO3(1+) bamb07.high.log

Standard orientation:

Center Number	Atomic Number	Atomic Type	Coordinates (Angstroms) X	Y	Z
1	6	0	-0.755674	-1.009155	0.077033
2	7	0	-2.077048	-0.898929	0.081561

3	6	0	-0.058239	-1.475418	-1.080437
4	6	0	-0.021128	-0.495416	1.218186
5	6	0	1.298545	-1.577134	-1.030880
6	6	0	1.378193	-0.688970	1.251970
7	6	0	2.016029	-1.204088	0.152886
8	1	0	-2.628667	-1.233559	-0.700890
9	1	0	-0.618028	-1.765916	-1.961780
10	1	0	-0.555973	-0.293873	2.136199
11	1	0	1.847240	-1.957019	-1.883364
12	1	0	1.928676	-0.412226	2.141142
13	1	0	3.089924	-1.348419	0.174621
14	8	0	-2.452087	1.073951	-0.518854
15	1	0	-3.350342	1.391382	-0.335858
16	1	0	-2.582486	-0.710184	0.938384
17	1	0	-1.217477	1.536046	0.250275
18	8	0	-0.352725	1.528723	0.788533
19	1	0	0.375524	1.892425	0.232494
20	8	0	1.712739	2.608563	-0.679447
21	1	0	1.588884	2.930754	-1.579804
22	1	0	2.252299	3.274284	-0.236754

SCF Done: E(RB+HF-LYP) = -516.120150496 A.U. after 1 cycles

Harmonic frequencies (cm**-1), IR intensities (KM/Mole)

	1	2	3
	A	A	A
Frequencies --	-321.2547	36.2945	50.1387
Red. masses --	4.3925	4.4719	4.8943
Frc consts --	0.2671	0.0035	0.0072
IR Inten --	85.6937	3.1464	2.1272

Zero-point correction= 0.180068 (a.u.)
Thermal correction to Energy= 0.192558
Thermal correction to Enthalpy= 0.193502
Thermal correction to Gibbs Free Energy= 0.141432
Sum of electronic and zero-point Energies= -515.940082
Sum of electronic and thermal Energies= -515.927593
Sum of electronic and thermal Enthalpies= -515.926648
Sum of electronic and thermal Free Energies= -515.978718

	E (Thermal)	CV	S
	KCal/Mol	Cal/Mol-Kelvin	Cal/Mol-Kelvin
Total	120.832	45.645	109.590

[6] TS geometries in Figure S3

==TS1(I), proton attach to N(2), by B3LYP/6-31G(d)==

Stoichiometry C6H18NO6(1+) bamb23px.log

Standard orientation:

Center Number	Atomic Number	Atomic Type	Coordinates (Angstroms) X	Y	Z
1	6	0	1.702841	0.454143	0.460657

2	7	0	0.350943	0.911212	0.755990
3	6	0	2.437738	1.032472	-0.570322
4	6	0	2.184009	-0.649523	1.170667
5	6	0	3.685095	0.492295	-0.891451
6	6	0	3.428103	-1.186622	0.829601
7	6	0	4.179023	-0.616947	-0.202730
8	1	0	-0.471407	0.183757	0.027109
9	1	0	2.048750	1.899285	-1.090512
10	1	0	1.609486	-1.077046	1.990116
11	1	0	4.272424	0.945994	-1.683909
12	1	0	3.817458	-2.032212	1.388870
13	1	0	5.151161	-1.027687	-0.456972
14	8	0	0.182234	2.291672	0.465339
15	1	0	-0.713456	2.340563	0.049932
16	1	0	0.164138	0.797994	1.757419
17	8	0	-1.157693	-0.455849	-0.706357
18	1	0	-2.069274	-0.539226	-0.235365
19	1	0	-0.718943	-1.351471	-0.811994
20	8	0	-3.328092	-0.186709	0.590321
21	1	0	-3.444446	0.751326	0.353005
22	1	0	-4.195676	-0.627141	0.409295
23	8	0	0.290198	-2.691832	-0.777535
24	1	0	0.385566	-3.223993	-1.582997
25	1	0	1.195141	-2.457933	-0.508623
26	8	0	-5.813308	-1.207777	0.084669
27	1	0	-5.946541	-2.068361	-0.341804
28	1	0	-6.400965	-1.217325	0.856306
29	8	0	-2.391014	2.264173	-0.644186
30	1	0	-2.322519	1.909690	-1.546721
31	1	0	-2.776955	3.150992	-0.741618

SCF Done: E(RB+HF-LYP) = -745.285590611 A.U. after 1 cycles

Harmonic frequencies (cm**-1), IR intensities (KM/Mole)

	1	2	3
	A	A	A
Frequencies --	-422.9000	15.2920	26.0400
Red. masses --	1.3769	4.8826	4.6648
Frc consts --	0.1451	0.0007	0.0019
IR Inten --	2254.2577	0.0972	0.8006

Zero-point correction= 0.255141 (a.u.)
Thermal correction to Energy= 0.275987
Thermal correction to Enthalpy= 0.276931
Thermal correction to Gibbs Free Energy= 0.202149
Sum of electronic and zero-point Energies= -745.030449
Sum of electronic and thermal Energies= -745.009604
Sum of electronic and thermal Enthalpies= -745.008660
Sum of electronic and thermal Free Energies= -745.083442

	E (Thermal)	CV	S
	KCal/Mol	Cal/Mol-Kelvin	Cal/Mol-Kelvin
Total	173.184	71.056	157.392

==TS2(I, 2H2O), water-dimer relay for the OH shift to the para carbon by B3LYP/6-31G(d)==

Stoichiometry   C6H18NO6(1+) bamb23.log

Standard orientation:

Center Number	Atomic Number	Atomic Type	Coordinates (Angstroms) X	Y	Z
1	6	0	0.379386	-0.833797	0.581942
2	7	0	-0.777055	-0.372956	1.009116
3	6	0	0.529309	-1.103023	-0.832673
4	6	0	1.519830	-0.944348	1.461310
5	6	0	1.766575	-1.312978	-1.347969
6	6	0	2.752987	-1.163037	0.939398
7	6	0	2.929666	-1.200019	-0.496953
8	1	0	-1.665579	-0.373440	0.332388
9	1	0	-0.367239	-1.113095	-1.445743
10	1	0	1.372391	-0.845159	2.533499
11	1	0	1.910326	-1.506134	-2.405201
12	1	0	3.622083	-1.262028	1.581646
13	1	0	3.870822	-1.574953	-0.884135
14	8	0	-0.751825	1.775583	0.777263
15	1	0	-0.924922	2.166170	1.653616
16	1	0	-0.913251	-0.227078	2.004944
17	1	0	0.833774	2.033001	0.457660
18	8	0	1.822255	2.201469	0.304399
19	1	0	1.882339	3.115642	-0.012790
20	1	0	2.764691	1.246803	-0.493817
21	8	0	3.467677	0.636157	-0.940539
22	1	0	4.309533	0.838115	-0.491332
23	8	0	-2.651861	-0.432536	-0.732535
24	1	0	-2.875409	0.533995	-0.889888
25	1	0	-3.493223	-0.873484	-0.454842
26	8	0	-2.952319	2.179630	-0.699870
27	1	0	-2.109013	2.212843	-0.166886
28	1	0	-2.792751	2.712354	-1.493323
29	8	0	-4.961313	-1.591673	0.201085
30	1	0	-5.690632	-0.963533	0.322646
31	1	0	-5.341979	-2.315151	-0.320997

SCF Done: E(RB+HF-LYP) = -745.224180208   A.U. after   1 cycles

Harmonic frequencies (cm**-1), IR

	1	2	3
	A	A	A
Frequencies --	-243.4196	20.9137	31.4155
Red. masses --	6.3227	4.8721	4.6200
Frc consts --	0.2207	0.0013	0.0027
IR Inten --	315.6146	1.3358	2.7542

Zero-point correction= 0.255555 (a.u.)
Thermal correction to Energy= 0.275050
Thermal correction to Enthalpy= 0.275994
Thermal correction to Gibbs Free Energy= 0.206878
Sum of electronic and zero-point Energies= -744.968625

Sum of electronic and thermal Energies= -744.949130
Sum of electronic and thermal Enthalpies= -744.948186
Sum of electronic and thermal Free Energies= -745.017303

	E (Thermal)	CV	S
	KCal/Mol	Cal/Mol-Kelvin	Cal/Mol-Kelvin
Total	172.596	69.722	145.468

==TS2(I, 2H2O) by B3LYP/6-311+G(d,p)==

Stoichiometry C6H18NO6(1+) bamb23.high.log

Standard orientation:

Center Number	Atomic Number	Atomic Type	Coordinates (Angstroms) X	Y	Z
1	6	0	0.354028	-0.858124	0.655827
2	7	0	-0.757215	-0.369538	1.134127
3	6	0	0.423133	-1.174831	-0.758287
4	6	0	1.539910	-0.971446	1.478603
5	6	0	1.624498	-1.416852	-1.328953
6	6	0	2.734080	-1.225261	0.899701
7	6	0	2.827612	-1.321400	-0.538869
8	1	0	-1.664918	-0.323081	0.516519
9	1	0	-0.502010	-1.182519	-1.323160
10	1	0	1.450968	-0.834208	2.550346
11	1	0	1.710642	-1.632952	-2.385621
12	1	0	3.635580	-1.311408	1.492959
13	1	0	3.740060	-1.718292	-0.964152
14	8	0	-0.587791	1.903323	0.885021
15	1	0	-0.827522	2.386364	1.688107
16	1	0	-0.817397	-0.122690	2.115182
17	1	0	0.944027	2.079419	0.513073
18	8	0	1.929518	2.158840	0.269918
19	1	0	2.117352	3.098666	0.178560
20	1	0	2.794612	1.188101	-0.573694
21	8	0	3.386472	0.524187	-1.083312
22	1	0	4.304652	0.716486	-0.844951
23	8	0	-2.738547	-0.344074	-0.507348
24	1	0	-2.854356	0.588447	-0.820197
25	1	0	-3.621453	-0.727763	-0.332273
26	8	0	-2.569298	2.253800	-0.886984
27	1	0	-1.785870	2.300488	-0.281244
28	1	0	-2.449965	2.888202	-1.598639
29	8	0	-5.212471	-1.477299	0.042377
30	1	0	-5.945545	-0.987131	0.430414
31	1	0	-5.610982	-2.162113	-0.505626

SCF Done: E(RB3LYP) = -745.560943498 A.U. after 2 cycles

Harmonic frequencies (cm**-1), IR intensities (KM/Mole)

	1	2	3
	A	A	A
Frequencies --	-260.6535	19.7089	30.0947
Red. masses --	5.4720	4.9775	4.7348

Frc consts -- 0.2190 0.0011 0.0025
IR Inten -- 265.0563 0.9907 1.5912

Zero-point correction= 0.253841 (a.u.)
Thermal correction to Energy= 0.274113
Thermal correction to Enthalpy= 0.275058
Thermal correction to Gibbs Free Energy= 0.203891
Sum of electronic and zero-point Energies= -745.307103
Sum of electronic and thermal Energies= -745.286830
Sum of electronic and thermal Enthalpies= -745.285886
Sum of electronic and thermal Free Energies= -745.357052

	E (Thermal)	CV	S
	KCal/Mol	Cal/Mol-Kelvin	Cal/Mol-Kelvin
Total	172.009	71.247	149.782

==TS3(I), proton removal from the para carbon by B3LYP/6-31G(d)==

Stoichiometry C6H18NO6(1+) bamb23x.log

Standard orientation:

Center Number	Atomic Number	Atomic Type	Coordinates (Angstroms) X	Y	Z
1	6	0	-3.358757	-0.591205	-0.350594
2	7	0	-4.486525	-1.130499	-0.849439
3	6	0	-3.104199	0.805657	-0.492444
4	6	0	-2.406797	-1.403179	0.328657
5	6	0	-1.954944	1.351418	0.011987
6	6	0	-1.251213	-0.853407	0.820899
7	6	0	-0.909024	0.520713	0.561221
8	1	0	-5.185487	-0.562524	-1.306743
9	1	0	-3.849255	1.435451	-0.970314
10	1	0	-2.614090	-2.459666	0.471993
11	1	0	-1.781248	2.421918	-0.056929
12	1	0	-0.527725	-1.464609	1.353380
13	1	0	-0.229145	0.277489	-0.569082
14	8	0	0.653323	0.198319	-1.589362
15	1	0	0.890501	1.142302	-1.715724
16	1	0	-4.699802	-2.110451	-0.728871
17	1	0	1.503677	-0.165399	-1.181356
18	8	0	2.906094	-0.134353	-0.264580
19	1	0	2.691209	-0.519152	0.626794
20	1	0	3.732375	-0.574804	-0.585908
21	8	0	0.040519	1.106409	1.422483
22	1	0	0.616652	1.720007	0.901938
23	8	0	1.810399	2.315200	-0.291783
24	1	0	2.503876	1.612303	-0.213204
25	1	0	2.253423	3.173887	-0.210867
26	8	0	5.183336	-1.431874	-1.020800
27	1	0	5.159551	-2.149974	-1.671292
28	1	0	6.019298	-0.968262	-1.180700
29	8	0	1.826254	-0.898975	2.073406
30	1	0	2.260608	-0.978583	2.936270
31	1	0	1.211464	-0.134227	2.133414

SCF Done: E(RB+HF-LYP) = -745.316104590 A.U. after 1 cycles

Harmonic frequencies (cm**-1), IR intensities (KM/Mole)

	1	2	3
	A	A	A
Frequencies --	-774.6379	28.0848	41.3362
Red. masses --	1.3591	4.9285	4.6691
Frc consts --	0.4805	0.0023	0.0047
IR Inten --	5530.0024	1.1495	3.1113

Zero-point correction= 0.256134 (a.u.)
Thermal correction to Energy= 0.275785
Thermal correction to Enthalpy= 0.276730
Thermal correction to Gibbs Free Energy= 0.208187
Sum of electronic and zero-point Energies= -745.059971
Sum of electronic and thermal Energies= -745.040319
Sum of electronic and thermal Enthalpies= -745.039375
Sum of electronic and thermal Free Energies= -745.107917

	E (Thermal)	CV	S
	KCal/Mol	Cal/Mol-Kelvin	Cal/Mol-Kelvin
Total	173.058	70.890	144.259

==TS2(I, 3H2O) by B3LYP/6-31G(d)==

Stoichiometry C6H18NO6(1+) bamb29ay.log

Standard orientation:

Center Number	Atomic Number	Atomic Type	Coordinates (Angstroms) X	Y	Z
1	6	0	0.195012	-1.200878	0.429668
2	7	0	-1.043082	-1.352409	0.873365
3	6	0	0.404497	-0.963443	-0.979825
4	6	0	1.321758	-1.247987	1.328746
5	6	0	1.671077	-0.811013	-1.454428
6	6	0	2.579653	-1.073600	0.848240
7	6	0	2.790894	-0.791780	-0.554063
8	1	0	-1.848012	-1.428321	0.184700
9	1	0	-0.461628	-0.958329	-1.632313
10	1	0	1.150277	-1.450549	2.382848
11	1	0	1.847542	-0.694954	-2.518888
12	1	0	3.441404	-1.127326	1.504094
13	1	0	3.780992	-0.975071	-0.955489
14	8	0	-2.112246	0.353046	1.456256
15	1	0	-1.388062	1.255292	0.280284
16	1	0	-1.218498	-1.665523	1.822002
17	1	0	0.459386	2.321037	0.047113
18	8	0	-0.947982	1.666524	-0.521170
19	1	0	-1.673320	2.098662	-0.999711
20	8	0	1.334168	2.447419	0.516935
21	1	0	2.543096	1.680671	-0.072999
22	1	0	1.450037	3.397973	0.662308
23	1	0	-1.740471	0.644139	2.308169
24	8	0	3.345336	1.142567	-0.425156

25	1	0	3.513169	1.466715	-1.328287
26	8	0	-2.847349	-1.356582	-1.095180
27	1	0	-3.362545	-0.509603	-0.980347
28	1	0	-3.496433	-2.056860	-1.262307
29	8	0	-3.910822	0.992774	-0.374883
30	1	0	-3.506465	0.870813	0.519804
31	1	0	-4.855071	1.164319	-0.238988

SCF Done: E(RB+HF-LYP) = -745.227804881 A.U. after 1 cycles

Harmonic frequencies (cm**-1), IR intensities (KM/Mole)

	1	2	3
	A	A	A
Frequencies --	-200.2358	37.0090	42.0401
Red. masses --	5.5050	5.3923	4.5774
Frc consts --	0.1300	0.0044	0.0048
IR Inten --	446.2789	6.8784	4.1186

Zero-point correction= 0.257686 (a.u.)
Thermal correction to Energy= 0.276687
Thermal correction to Enthalpy= 0.277631
Thermal correction to Gibbs Free Energy= 0.211438
Sum of electronic and zero-point Energies= -744.970119
Sum of electronic and thermal Energies= -744.951118
Sum of electronic and thermal Enthalpies= -744.950174
Sum of electronic and thermal Free Energies= -745.016367

	E (Thermal)	CV	S
	KCal/Mol	Cal/Mol-Kelvin	Cal/Mol-Kelvin
Total	173.624	69.957	139.316

==TS2(I, 3H2O) by B3LYP/6-311+G(d,p)==

Stoichiometry C6H18NO6(1+) bamb29ay.high.log

Standard orientation:

Center Number	Atomic Number	Atomic Type	Coordinates (Angstroms) X	Y	Z
1	6	0	0.099135	-1.126594	0.528021
2	7	0	-1.138074	-1.177170	0.958274
3	6	0	0.347127	-1.094275	-0.897881
4	6	0	1.210717	-1.104201	1.451948
5	6	0	1.622354	-1.084849	-1.357797
6	6	0	2.478715	-1.087038	0.982949
7	6	0	2.726710	-1.046652	-0.439872
8	1	0	-1.943840	-1.307297	0.284644
9	1	0	-0.506391	-1.124180	-1.564540
10	1	0	1.009760	-1.133402	2.517213
11	1	0	1.823095	-1.115737	-2.421352
12	1	0	3.325239	-1.097047	1.656893
13	1	0	3.696903	-1.375963	-0.786882
14	8	0	-2.170092	0.779284	1.281031
15	1	0	-1.271936	1.579522	0.206376
16	1	0	-1.341913	-1.272499	1.945365

17	1	0	0.815628	2.431088	-0.060698
18	8	0	-0.678049	1.923923	-0.525937
19	1	0	-1.245040	2.377659	-1.158178
20	8	0	1.756194	2.510892	0.254396
21	1	0	2.812820	1.528959	-0.266152
22	1	0	1.925079	3.420636	0.516471
23	1	0	-2.102538	1.060369	2.203429
24	8	0	3.494876	0.831176	-0.562939
25	1	0	3.764817	1.052473	-1.465033
26	8	0	-2.987306	-1.474874	-0.947638
27	1	0	-3.588016	-0.684148	-0.922236
28	1	0	-3.524543	-2.244571	-1.161386
29	8	0	-4.156190	0.843594	-0.483092
30	1	0	-3.614601	0.953363	0.335609
31	1	0	-5.052020	1.140662	-0.298232

SCF Done: E(RB+HF-LYP) = -745.564116896 A.U. after 1 cycles
Convg = 0.8325D-08 -V/T = 2.0047
S**2 = 0.0000

Harmonic frequencies (cm**-1), IR intensities (KM/Mole)

	1	2	3
	A	A	A
Frequencies --	-201.4464	24.8619	34.6272
Red. masses --	5.5240	5.3714	5.4272
Frc consts --	0.1321	0.0020	0.0038
IR Inten --	420.0625	1.1110	3.7823

Zero-point correction= 0.255771 (a.u.)
Thermal correction to Energy= 0.275360
Thermal correction to Enthalpy= 0.276304
Thermal correction to Gibbs Free Energy= 0.207740
Sum of electronic and zero-point Energies= -745.308346
Sum of electronic and thermal Energies= -745.288757
Sum of electronic and thermal Enthalpies= -745.287813
Sum of electronic and thermal Free Energies= -745.356377

	E (Thermal)	CV	S
	KCal/Mol	Cal/Mol-Kelvin	Cal/Mol-Kelvin
Total	172.791	70.848	144.305

==TS2(I,[1,3]OH shift) by B3LYP/6-31G(d)==
Stoichiometry C6H18NO6(1+) bamb07ext.log

Standard orientation:

Center Number	Atomic Number	Atomic Type	Coordinates (Angstroms) X	Y	Z
1	6	0	-0.375046	-0.765105	-0.469818
2	7	0	0.764571	-0.283072	-0.942562
3	6	0	-0.449633	-1.205870	0.896795
4	6	0	-1.571845	-0.721263	-1.288986
5	6	0	-1.627906	-1.714833	1.361055
6	6	0	-2.746335	-1.329425	-0.792791
7	6	0	-2.772932	-1.793697	0.503704

8	1	0	1.688255	-0.323760	-0.314754
9	1	0	0.448980	-1.159481	1.503800
10	1	0	-1.471208	-0.528187	-2.350284
11	1	0	-1.701357	-2.086725	2.377604
12	1	0	-3.619415	-1.402493	-1.431591
13	1	0	-3.680038	-2.250201	0.889373
14	8	0	0.649992	1.805601	-0.766332
15	1	0	1.030637	2.168826	-1.589159
16	1	0	0.848089	-0.134186	-1.943131
17	1	0	-0.918363	1.709134	-0.952826
18	8	0	-1.860628	1.374707	-1.135489
19	1	0	-2.389229	1.508574	-0.307042
20	8	0	-3.454215	1.699267	1.091751
21	1	0	-3.186032	2.347145	1.762465
22	1	0	-4.332307	1.995959	0.803522
23	8	0	2.717194	-0.379263	0.709437
24	1	0	2.868053	0.582940	0.940343
25	1	0	3.582068	-0.739775	0.390099
26	8	0	2.759229	2.258156	0.900171
27	1	0	1.916810	2.284567	0.378836
28	1	0	2.583071	2.725791	1.730129
29	8	0	5.063714	-1.345640	-0.332158
30	1	0	5.794647	-0.709927	-0.383898
31	1	0	5.445237	-2.128159	0.095650

SCF Done: E(RB+HF-LYP) = -745.227287285 A.U. after 1 cycles

Harmonic frequencies (cm**-1), IR intensities (KM/Mole)

	1	2	3
	A	A	A
Frequencies --	-259.0728	19.7993	29.5504
Red. masses --	6.2507	5.0401	4.8562
Frc consts --	0.2472	0.0012	0.0025
IR Inten --	103.5377	2.5350	0.7964

Zero-point correction= 0.255153 (a.u.)
Thermal correction to Energy= 0.275556
Thermal correction to Enthalpy= 0.276500
Thermal correction to Gibbs Free Energy= 0.204651
Sum of electronic and zero-point Energies= -744.972135
Sum of electronic and thermal Energies= -744.951732
Sum of electronic and thermal Enthalpies= -744.950788
Sum of electronic and thermal Free Energies= -745.022636

	E (Thermal)	CV	S
	KCal/Mol	Cal/Mol-Kelvin	Cal/Mol-Kelvin
Total	172.914	71.141	151.218

==TS(I,[1.3]OH shift) by B3LYP/6-311+G(d,p)==

Stoichiometry C6H18NO6(1+) bamb07ext.high.log

Standard orientation:

Center Number	Atomic Number	Atomic Type	Coordinates (Angstroms) X	Y	Z

1	6	0	-0.306067	-0.835695	-0.521640
2	7	0	0.755964	-0.330685	-1.112345
3	6	0	-0.255081	-1.183538	0.874168
4	6	0	-1.554370	-0.956366	-1.254065
5	6	0	-1.350173	-1.752115	1.448138
6	6	0	-2.634409	-1.606955	-0.641037
7	6	0	-2.534755	-1.983493	0.680653
8	1	0	1.705558	-0.258147	-0.583709
9	1	0	0.667071	-1.016954	1.416930
10	1	0	-1.552964	-0.788345	-2.321852
11	1	0	-1.328199	-2.050778	2.488546
12	1	0	-3.540426	-1.786757	-1.204135
13	1	0	-3.372619	-2.479692	1.157285
14	8	0	0.480616	1.837963	-1.041955
15	1	0	0.900642	2.264369	-1.803700
16	1	0	0.742552	-0.189357	-2.115257
17	1	0	-1.061076	1.611771	-1.121843
18	8	0	-1.980606	1.194142	-1.183340
19	1	0	-2.488923	1.423995	-0.376322
20	8	0	-3.559084	1.821050	1.031926
21	1	0	-3.245566	2.310415	1.800830
22	1	0	-4.429511	2.183441	0.830120
23	8	0	2.860264	-0.161501	0.377539
24	1	0	2.799973	0.706538	0.835753
25	1	0	3.797379	-0.390747	0.218853
26	8	0	1.882810	2.152058	1.276056
27	1	0	1.344117	2.264739	0.460329
28	1	0	2.139485	3.024113	1.588763
29	8	0	5.482766	-0.918351	-0.077182
30	1	0	6.177111	-0.436209	-0.538638
31	1	0	5.928643	-1.571520	0.472810

SCF Done: E(RB+HF-LYP) = -745.565637481 A.U. after 1 cycles
Convg = 0.5312D-08 -V/T = 2.0047
S**2 = 0.0000

Harmonic frequencies (cm**-1), IR intensities (KM/Mole)

	1	2	3
	A	A	A
Frequencies --	-241.7999	19.6131	25.4280
Red. masses --	4.9481	4.8343	4.8214
Frc consts --	0.1705	0.0011	0.0018
IR Inten --	73.9198	2.0041	2.2258

Zero-point correction= 0.253634 (a.u.)
Thermal correction to Energy= 0.274971
Thermal correction to Enthalpy= 0.275915
Thermal correction to Gibbs Free Energy= 0.201648
Sum of electronic and zero-point Energies= -745.312003
Sum of electronic and thermal Energies= -745.290666
Sum of electronic and thermal Enthalpies= -745.289722
Sum of electronic and thermal Free Energies= -745.363989

E (Thermal)	CV	S
KCal/Mol	Cal/Mol-Kelvin	Cal/Mol-Kelvin

Total 172.547 73.253 156.308

[7] A TS geometry in Figure S4

== A TS geometry of conversion, (HO)H2N(+)-C6H5 + H3O(+)(H2O)5 ->
H2N-C5H5-OH(+) + H3O(+)(H2O)5.

Stoichiometry C6H21NO7(2+) bamb29b.log

Standard orientation:

Center Number	Atomic Number	Atomic Type	Coordinates (Angstroms) X	Y	Z
1	6	0	1.712285	-1.714826	0.596858
2	7	0	2.852656	-1.423163	1.214299
3	6	0	0.485593	-1.658709	1.350892
4	6	0	1.706044	-1.937294	-0.827861
5	6	0	-0.703385	-1.741652	0.681383
6	6	0	0.502871	-2.046719	-1.469790
7	6	0	-0.702449	-1.879292	-0.734982
8	1	0	2.900556	-1.382367	2.231196
9	1	0	0.523936	-1.508936	2.426426
10	1	0	2.646659	-2.006406	-1.367469
11	1	0	-1.646359	-1.628607	1.202336
12	1	0	0.462488	-2.217440	-2.540240
13	1	0	-1.661335	-1.902462	-1.249562
14	8	0	2.954599	0.631255	1.495623
15	1	0	3.876509	0.954584	1.553990
16	1	0	3.747924	-1.525721	0.736294
17	1	0	2.431017	1.084383	0.460431
18	8	0	1.932342	1.399405	-0.562352
19	1	0	2.164456	2.335247	-0.859803
20	1	0	0.930368	1.288683	-0.599866
21	8	0	-0.630067	0.839102	-0.972964
22	1	0	-0.897831	1.272691	-1.800295
23	1	0	-1.455953	0.853919	-0.376669
24	8	0	-2.765262	0.501145	0.456815
25	1	0	-3.396976	0.021033	-0.119570
26	1	0	-3.261914	1.233682	0.902098
27	8	0	-4.040301	2.570597	1.695928
28	1	0	-4.368211	2.479814	2.603962
29	1	0	-4.659009	3.179632	1.264164
30	8	0	-3.968231	-1.333376	-1.287919
31	1	0	-4.383961	-1.074738	-2.127109
32	1	0	-4.600712	-1.955278	-0.891073
33	8	0	2.425753	3.846131	-1.391269
34	1	0	2.984161	4.051854	-2.157316
35	1	0	2.419177	4.645454	-0.841913

SCF Done: E(RB+HF-LYP) = -821.925402726 A.U. after 1 cycles

Harmonic frequencies (cm**-1), IR intensities (KM/Mole),

	1	2	3
	A	A	A
Frequencies --	-720.6259	15.8323	26.3952

Red. masses -- 1.3323 4.9673 4.9239
Frc consts -- 0.4076 0.0007 0.0020
IR Inten -- 1808.2917 0.7000 0.8466

Zero-point correction= 0.288248 (a.u.)
Thermal correction to Energy= 0.312996
Thermal correction to Enthalpy= 0.313940
Thermal correction to Gibbs Free Energy= 0.230997
Sum of electronic and zero-point Energies= -821.637155
Sum of electronic and thermal Energies= -821.612407
Sum of electronic and thermal Enthalpies= -821.611462
Sum of electronic and thermal Free Energies= -821.694406

	E (Thermal)	CV	S
	KCal/Mol	Cal/Mol-Kelvin	Cal/Mol-Kelvin
Total	196.408	84.076	174.569

[8] A TS geometry in Figure S5.
== A TS geomery following Scheme 8==

Stoichiometry C6H23NO8(2+) bamb29aj.log

Standard orientation:

Center Number	Atomic Number	Atomic Type	Coordinates (Angstroms) X	Y	Z
1	6	0	-1.424652	-1.854349	-0.635173
2	7	0	-2.690995	-1.960362	-1.028379
3	6	0	-0.495547	-1.075905	-1.415253
4	6	0	-1.000720	-2.512724	0.574771
5	6	0	0.814072	-1.024554	-1.033765
6	6	0	0.316331	-2.457664	0.934182
7	6	0	1.241024	-1.715194	0.142432
8	1	0	-2.967917	-1.660607	-1.960284
9	1	0	-0.844805	-0.559790	-2.305329
10	1	0	-1.724224	-3.073308	1.160298
11	1	0	1.544625	-0.444710	-1.587783
12	1	0	0.666469	-2.986081	1.814467
13	1	0	2.305804	-1.798151	0.338331
14	8	0	-3.984570	-0.592316	-0.151771
15	1	0	-3.339113	0.518148	-0.191879
16	1	0	-3.326057	-2.607684	-0.566739
17	1	0	-2.012558	1.332753	0.583611
18	8	0	-2.638118	1.424979	-0.214794
19	1	0	-3.126270	2.304115	-0.144438
20	8	0	-1.106127	0.840953	1.784851
21	1	0	-0.124924	0.827052	1.671012
22	1	0	-1.280716	1.268220	2.637973
23	1	0	-4.793032	-0.518725	-0.697706
24	8	0	1.550238	0.292870	1.441316
25	1	0	2.237038	0.678157	0.782447
26	1	0	2.029658	0.139453	2.273772
27	8	0	3.243687	0.910449	-0.384982
28	1	0	3.982197	0.271535	-0.284692
29	1	0	3.634839	1.810823	-0.521357

30	8	0	4.730629	-1.390849	0.041685
31	1	0	5.399129	-1.478441	0.741061
32	1	0	5.144664	-1.797800	-0.737203
33	8	0	4.243536	3.415507	-0.762785
34	1	0	4.478673	3.726559	-1.650487
35	1	0	4.858093	3.867383	-0.164376
36	8	0	-3.818611	3.764877	0.078104
37	1	0	-4.672969	3.925367	0.508250
38	1	0	-3.699999	4.488826	-0.556447

SCF Done: E(RB+HF-LYP) = -898.366269299 A.U. after 1 cycles

Harmonic frequencies (cm**-1), IR intensities (KM/Mole)

	1	2	3
	A	A	A
Frequencies --	-476.0517	16.6846	21.7526
Red. masses --	1.9223	5.0196	5.1654
Frc consts --	0.2567	0.0008	0.0014
IR Inten --	1686.1797	1.0734	0.5501

Zero-point correction= 0.314099 (a.u.)
Thermal correction to Energy= 0.341383
Thermal correction to Enthalpy= 0.342327
Thermal correction to Gibbs Free Energy= 0.252910
Sum of electronic and zero-point Energies= -898.052170
Sum of electronic and thermal Energies= -898.024887
Sum of electronic and thermal Enthalpies= -898.023943
Sum of electronic and thermal Free Energies= -898.113360

	E (Thermal)	CV	S
	KCal/Mol	Cal/Mol-Kelvin	Cal/Mol-Kelvin
Total	214.221	92.447	188.194
